# C(sp^2^)─H Bond Activation with a Heterometallic Nickel–Aluminium Complex

**DOI:** 10.1002/anie.202512684

**Published:** 2025-07-23

**Authors:** Joseph A. Zurakowski, Benedek Stadler, Marcus W. Drover, Mark R. Crimmin

**Affiliations:** ^1^ Department of Chemistry Molecular Sciences Research Hub Imperial College London 82 Wood Lane, Shepherds Bush London W12 0BZ UK; ^2^ Department of Chemistry Western University 1151 Richmond Street London ON N8K 3G6 Canada; ^3^ Department of Chemistry and Biochemistry The University of Windsor 401 Sunset Avenue Windsor ON N9B 3P4 Canada

**Keywords:** Aluminum, Catalysis, C─H activation, Heterometallic complex, Nickel

## Abstract

Herein we present a ligand‐catalyzed bond‐breaking and making process that occurs with a Ni–Al heterometallic complex. While combinations of Ni pre‐catalysts and Al additives are known for site‐selective C─H functionalization, detailed studies of such systems are rare. Combining [Ni(COD)_2_] and [(BDI)AlH_2_] (**1**; BDI = 2,6‐diisopropylphenyl‐β‐methyldiketiminate) results in facile formation of a Ni–Al heterometallic complex [(*η* ^4^‐COD)Ni{(*μ*‐H)_2_Al(BDI)}] (**2**; COD = 1,5‐cyclooctadiene). Once generated, this species can effect the C(sp^2^)─H activation of 4‐dimethylaminopyridine (DMAP) or pyridine with concomitant H_2_ evolution, a process that is accelerated through addition of PCy_3_. The mechanism was studied through kinetics, kinetic isotope effects (KIEs), isotope labeling studies, and modeling. The reaction is 1^st^ order in PCy_3_ and proceeds with a KIE of 0.9–1.1. Support is provided for a pathway involving the synergistic action of both metals, promoted by reversible coordination of the phosphine. The data suggest that both C─H oxidative addition and H_2_ reductive elimination steps in this system are readily accessible and are not rate limiting. This finding has implications for future catalyst design using combinations of Ni and Al metals and suggests that control of ligand exchange steps may be the most important consideration in determining the rate of reaction.

## Graphical abstract

Despite the prevalence of Ni/Al catalysts for pyridine C─H functionalization, mechanistic details of such systems remain scarce. Herein, we present the discovery of PCy_3_‐catalyzed bond‐breaking and making processes that occur in the coordination sphere of a novel Ni─Al heterometallic complex. The reaction is 1^st^ order in PCy_3_ and proceeds with a low KIE of 0.9–1.1. These data suggest that both C─H activation and H_2_ reductive elimination steps in this system are low energy, are readily accessible, and are not rate limiting.

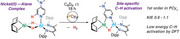

## Introduction

Controlling the reactivity of 1st‐row transition metal complexes, such as those based on nickel, can be challenging.^[^ ^1^ ^]^ As applications of these types of complexes continue to grow, so does our appreciation of the diversity and complexity of the mechanisms by which they can operate. The limited radial extension of 3d orbitals, the possibility of both one‐ (radical) and two‐electron pathways, and the role of non‐innocent ligands are all important considerations that complicate the study of both stoichiometric and catalytic processes.^[^ ^2^, ^3^, ^4^, ^5^ ^]^

**Figure 1:**
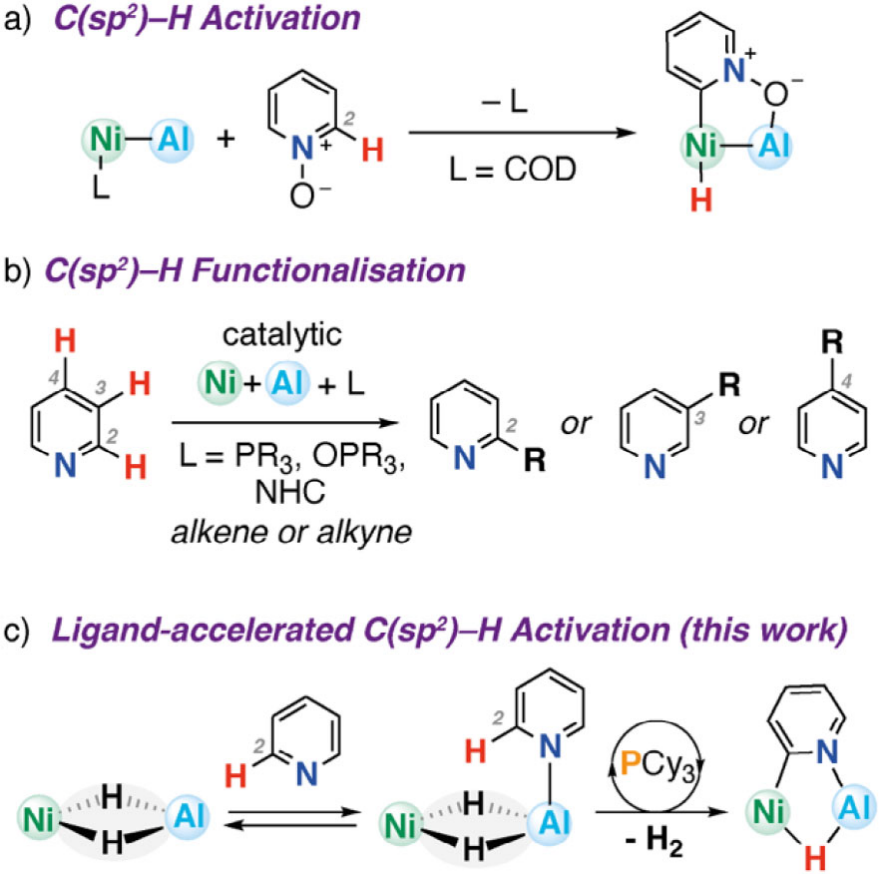
a) Catalytic C(sp^2^)─H functionalization of pyridines catalyzed by Ni/Al mixtures. b) Oxidative addition of pyridine N‐oxide to a heterometallic Ni–Al complex. c) Ligand‐accelerated C(sp^2^)─H activation and H_2_ elimination at a Ni─Al heterometallic complex (this work).

One emerging strategy to control the reactivity of 1st‐row metals is through inclusion of a second main group metal or semi‐metal in the coordination sphere.^[^ ^6^, ^7^, ^8^ ^]^ For example, Lu and coworkers have isolated a Ni─Al heterometallic complex and demonstrated that the two metals work cooperatively in the C(sp^2^)─H bond activation of pyridine N‐oxide (Figure 1a).^[^ ^9^ ^]^ Studies from one of our groups have shown that a related Fe─Al heterometallic complex is capable of activation of the C(sp^2^)─H bonds of both pyridines and alkenes.^[^ ^10^, ^11^ ^]^ Despite their potential, catalytic turnover with these well‐defined systems is still uncommon, and our mechanistic understanding is limited to isolated bond‐breaking steps.^[^ ^12^ ^]^ This is perhaps surprising, as use of Lewis‐acidic additives in Ni‐catalyzed C(sp^2^)─H functionalization is widespread.^[^ ^13^, ^14^, ^15^, ^16^, ^17^, ^18^, ^19^, ^20^ ^]^ In particular, Lewis‐acidic trialkyl aluminum additives in combination with phosphine, phosphine oxide, or *N*‐heterocyclic carbene ligands have been used to control the site‐selectivity, chemoselectivity, and enantioselectivity of the catalytic C─H alkynylation and alkenylation of pyridines (Figure 1b). These systems demonstrate exquisite control, with the potential to access C_2_, C_3_, and C_4_ functionalized products depending on the nature of the catalytic mixture. Related heterometallic complexes based on 2^nd^ and 3^rd^ row transition metals, including Rh–B,^[^ ^21^ ^]^ Ir–B,^[^ ^22^, ^23^, ^24^ ^]^ Rh–Al,^[^ ^25^, ^26^, ^27^ ^]^ Rh–In,^[^ ^28^ ^]^ and Pd–Al^[^ ^29^ ^]^ complexes are finding increasing use as catalysts for the functionalization of C(sp^2^)─H bonds. A deeper understanding of multistep reaction sequences involving well‐defined Ni─Al heterometallic complexes, alongside identification of new strategies to promote these, could underpin further developments in catalysis.

In this paper, we report the isolation of a novel Ni─Al heterometallic complex through a straightforward reaction of an aluminum dihydride complex with [Ni(COD)_2_]. We demonstrate that this species is capable of a room temperature, multistep reaction process involving the sequential C(sp^2^)─H activation of the 2‐position of 4‐dimethylaminopyridine or pyridine and elimination of H_2_. We provide a detailed mechanistic study that suggests the reaction occurs through stepwise oxidative addition and reductive elimination steps that involve synergistic action of both metals in the heterometallic complex. Remarkably, we discovered that the entire reaction sequence can be catalyzed by the addition of an exogenous phosphine ligand. Ligand acceleration effects are well established in single‐site transition metal complexes, being an underlying principle behind asymmetric catalysis, but examples in heterometallic complexes are incredibly rare. The discovery that ligand acceleration gives access to multiple steps requisite for catalytic turnover in this system could have important implications for further catalyst design and screening.

## Results and Discussion

### Synthesis of Ni–Al Heterometallic Complexes

**Scheme 1:**
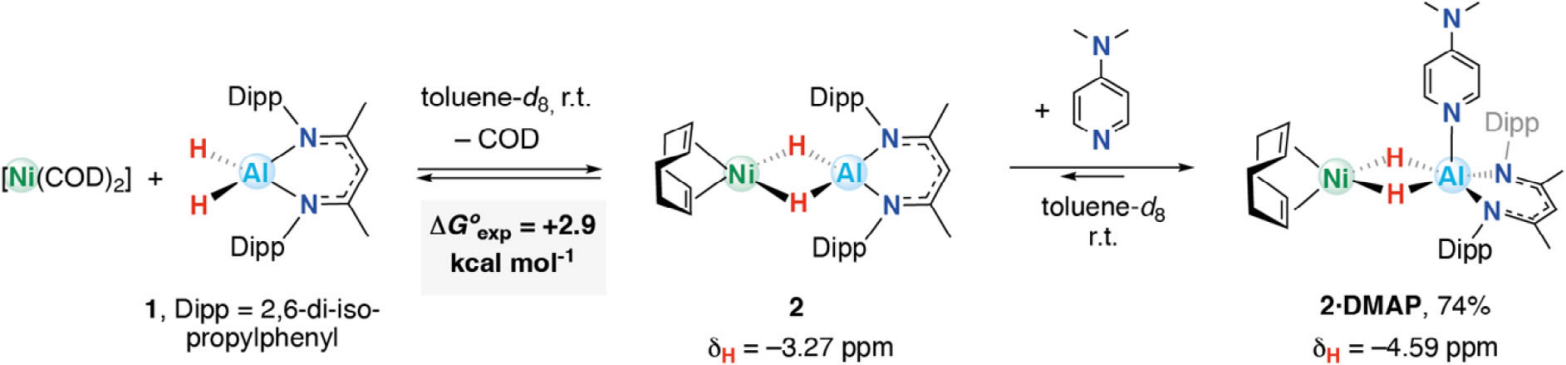
Reversible reaction of 1 and [Ni(COD)_2_] to form 2 and trapping with DMAP to form 2·DMAP.

Combining equimolar amounts of [(BDI)AlH_2_] (**1**; BDI = 2,6‐diisopropylphenyl‐β‐methyldiketiminate) and [Ni(COD)_2_] in toluene‐*d*_8_ at room temperature gave a gradual color change from yellow to red over the course of 1 h due to the formation of the heterometallic dihydride complex **2** (Scheme 1). ^1^H NMR spectroscopy revealed an upfield signal at *δ*_H_ = −3.27 ppm assigned to the hydride ligands of **2** (*c.f. δ*_H_  =  4.50 ppm for **1**). This signal integrated for 2H with respect to a new (*^i^* Pr)‐CH signal (4H) at *δ*_H_ = 3.15 ppm, suggesting that the new species had *C*_2v_ symmetry in solution. The reaction did not go to completion, and after 18 h, ^1^H NMR spectroscopy showed both **2** along with starting materials, consistent with an equilibrium process. Addition of excess (10 equiv.) COD to mixtures containing **2** resulted in the reformation of **1** and [Ni(COD)_2_]. A van‘t Hoff analysis was conducted to better understand and quantify the reversible process. The Gibbs free energy of binding was determined experimentally as Δ*G*°_exp_ = +2.9 kcal mol^−1^ (Figures S25–S27) and calculated by density functional theory (DFT) as Δ*G*°_calc_ = +4.0 kcal mol^−1^ (vide infra).

Acknowledging that the addition of a Lewis base might help to stabilize the product and thus push the reaction to completion by granting a five‐coordinate aluminum center, **1** was combined with 1 equiv. of [Ni(COD)_2_] and 4‐dimethylaminopyridine (DMAP) in toluene‐*d*_8_ (Scheme 1). A dark red powder consistent with the formulation of **2·DMAP** was isolated following workup in 74% yield. **2·DMAP** is again in equilibrium with its starting materials, as addition of 10 equiv. COD to **2** reformed **1**, [Ni(COD)_2_] and DMAP (Figure S28). Based on ^1^H NMR spectroscopic measurements, *K*_eq_ (**2·DMAP**) > *K*_eq_ (**2**) with the addition of DMAP appearing to favor product formation. This is likely due to the increased coordination number at aluminum leading to more hydridic [Al–H] units that have a greater affinity for nickel. In terms of diagnostic data, a new hydride signal of **2·DMAP** appeared in the ^1^H NMR spectrum with *δ*_H_ = ─4.59 ppm, integrating for 2H. The aromatic C─H signals for DMAP are found at *δ*_H_ = 8.88 and 5.94 ppm. These resonances are sharp and notably distinct from those of free DMAP (*δ*_H_ = 8.48 and 6.11 ppm). [Ni(COD)_2_] appeared requisite to observe the five‐coordinate aluminum center in **2·DMAP**, as combining an equimolar mixture of DMAP and **1** in C_6_D_6_ yielded no apparent reaction at 298 K.^[^ ^30^ ^]^ Consistent with this observation, DMAP coordination to **1** was calculated to be endergonic with Δ*G*_calc_ = +3.6 kcal mol^−1^, whereas addition of DMAP to **2** is exergonic with Δ*G*_calc_ = −3.7 kcal mol^−1^ (Table S9).

**Figure 2:**
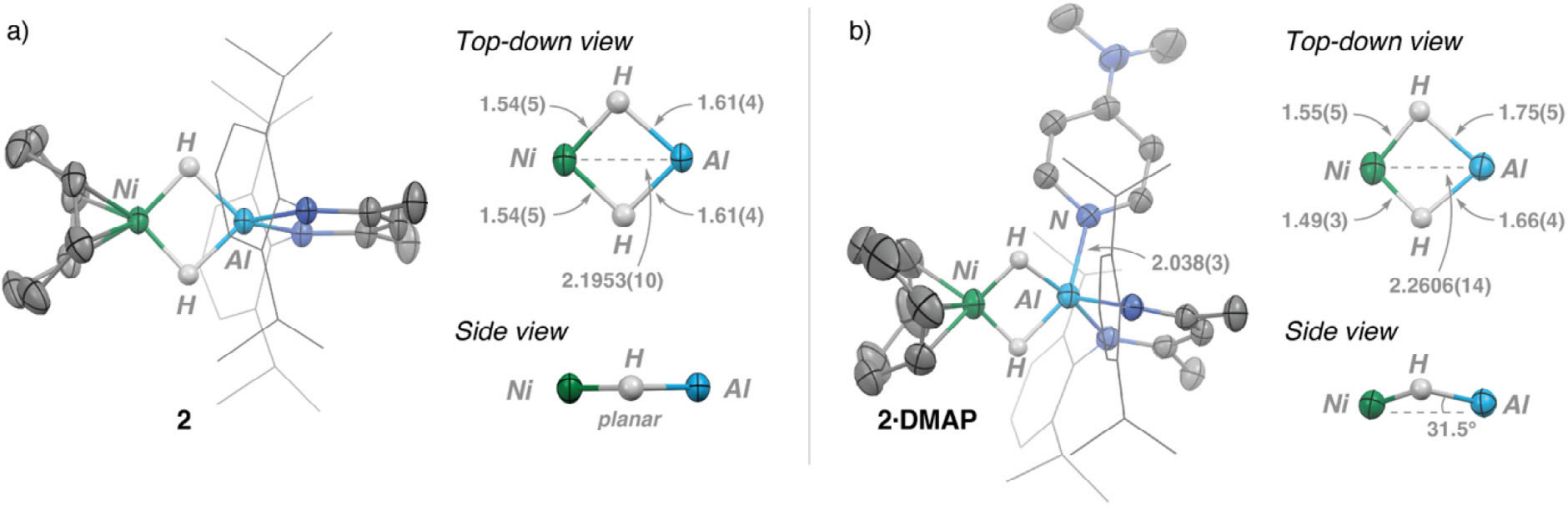
a) Solid‐state structure of **2** with top‐down and side views of the Ni–(*μ*‐H)_2_–Al core. b) Solid‐state structure of **2⋅DMAP**, with top‐down and side views of the Ni–(*μ*‐H)_2_–Al core. Ellipsoids are drawn at 50% probability, Dipp groups have been truncated for visual clarity, and only hydrogen atoms directly bound to metals are shown. Bond lengths are given in Å.

Crystals of **2** were grown from a saturated *n*‐pentane solution at −35 °C overnight. The solid‐state structure of **2** (Figure 2a) features two tetrahedral metal centers (*τ*_4_(Al) = 0.80; *τ*_4_(Ni) = 0.84) and maintains a Ni—Al distance of 2.1953(10) Å, falling within the sum of covalent radii (*∑*_Ni,Al_ = 2.45 Å).^[^ ^31^, ^32^ ^]^ Such short metal–metal distances are not always indicative of a chemical bond (vide infra). The two bridging hydrides were located from the Fourier difference map; these bridge symmetrically and are 1.54(5) Å from Ni and 1.61(4) Å from Al. The crystallographic *C*_2_ symmetry of the structure is further reflected in the planarity of the H─Ni─H′─Al core of **2**. The average Al─H distances in **2** are around 0.1 Å longer than those in **1**, but the errors and uncertainty of these measurements are such that definitive conclusions cannot be drawn. The solid‐state structure of **2·DMAP** was also confirmed by X‐ray diffraction (Figure 2b). **2·DMAP** now features a five‐coordinate aluminum center with *τ*_5_(Al) = 0.34, indicating a near‐square pyramidal geometry. Upon DMAP coordination, the complex features a slight elongation of the Ni─Al (2.2606(14) Å) distance along with the introduction of asymmetry within the heterometallic core. Unlike **2**, the Ni–(*μ*‐H)_2_–Al core in **2·DMAP** is nonplanar, with the Ni atom showing the largest deviation of 0.17(4) Å from the mean plane.

To the best of our knowledge, **2** and **2·DMAP** represent the first examples of structurally authenticated heterometallic Ni─Al complexes with two bridging hydride ligands. A Cambridge Structural Database search reveals only a handful of related structures that feature the Ni‐(*μ*‐H)‐Al unit, with none containing more than one bridging hydride. For example, in 1990, Pörschke described a heterometallic complex consisting of a Me_2_AlH moiety capped by 1‐azabicyclo[2.2.2]octane, coordinated to [Ni(CDT)] (CDT = 1,5,9‐cyclododecatriene) via a bridging hydride ligand.^[^ ^12^, ^33^, ^34^, ^35^ ^]^ This compound has a Ni─Al distance of 2.731(1) Å that is much longer than those found in **2** or **2⋅DMAP** but has comparable Ni─H and Al─H distances.

### Electronic Structures of 2 and 2·DMAP

**Figure 3:**
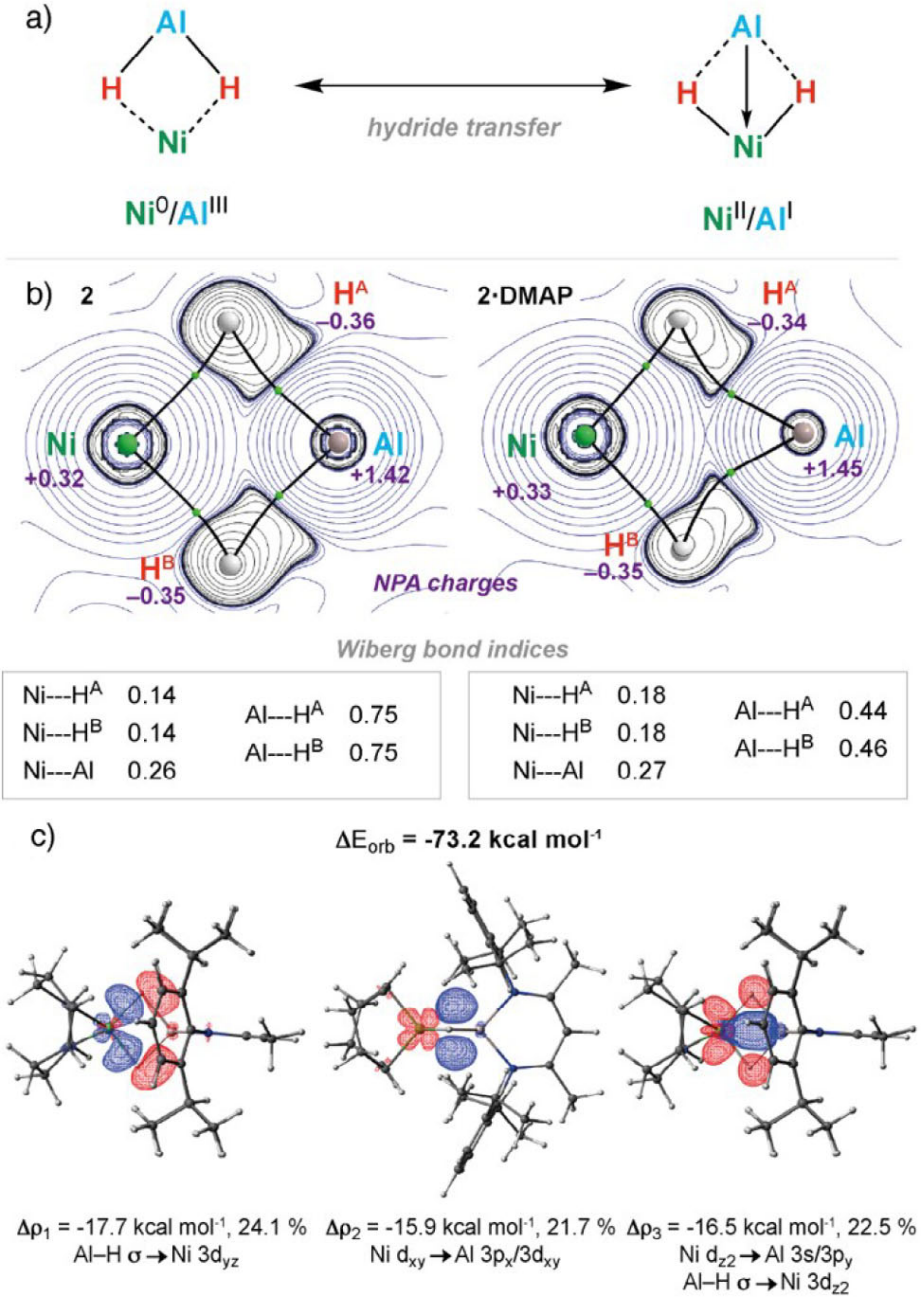
a) Extreme bonding models for **2** and **2·DMAP**. b) QTAIM and NBO analysis of left: **2** and right: **2·DMAP**. The background shows the Laplacian of the electron density. c) ETS‐NOCV analysis for **2** (deformation density plots for the first three contributions). For the fragmentation, see Figure S63, and for the next three contributions, see Table S14. Isovalue = 0.005. Charge flows from red to blue.

The heterometallic complexes **2** and **2·DMAP** were assessed by DFT calculations to elucidate the most appropriate bonding model.^[^ ^36^, ^37^ ^]^ Due to their high flexibility, careful conformational sampling was required to give accurate energies.^[^ ^38^ ^]^ In addition, Fractional Occupation number weighted electron Density (FOD) analysis revealed that the bimetallic complexes exhibited strong correlation effects (Figures S54–S63).^[^ ^39^ ^]^ After extensive benchmarking (Tables S3–S8), we report our results at the TPSS‐D4/‌def2‐QZVPP/SMD(toluene)//r^2^SCAN‐3c/CPCM(toluene) level of theory. A series of canonical forms can be considered, including formulation as either Ni(0)/Al(III) or Ni(II)/Al(I) complexes depending on the nature of the metal–metal and metal–hydride bonding interactions (Figure 3a). Based on the analysis presented below, the most suitable bonding description of **2** and **2·DMAP** is as bis(σ‐alane) complexes of nickel with formal Ni(0)/Al(III) oxidation states.

Quantum theory of atoms in molecules (QTAIM) analysis for **2** and **2·DMAP** showed no bond critical point (BCP) between Ni and Al, suggestive of limited metal–metal bonding. The presence of a BCP between each of the hydride ligands and metal centers indicated that the bonding in the bimetallic cores is dominated by bridging interactions through the hydrides (Figure 3b). These BCPs featured significant positive values for the Laplacian of the electron density (∇^2^ *ρ*(r)), indicative of the largely electrostatic nature of the metal–hydride interactions (Table S13).

Natural bond orbital (NBO) analysis gave a qualitatively similar model, fragmenting **2** and **2·DMAP** across the Ni─H bonds. Charges from natural population analysis (NPA) were positive on both Al (**2**, +1.42; **2·DMAP**, +1.45) and Ni (**2**, +0.32; **2·DMAP,** +0.33) and negative on the hydride ligands (**2**, −0.35/−0.36; **2·DMAP**, −0.34/−0.35). The Wiberg bond indices (WBIs) showed that the bimetallic core features minimal Ni─Al bonding (**2**, 0.26; **2·DMAP**, 0.28) along with 3c‐2e interactions in which the hydrides are most strongly associated with the Al rather than the Ni nuclei (**2**, Al─H = 0.75/0.75, Ni─H = 0.14/0.14; **2·DMAP** Al─H = 0.44/0.46, Ni─H = 0.18/0.18). Mayer bond indices suggested a drop in the Ni─Al (**2**, 0.90; **2·DMAP**, 0.55) covalency upon coordination of DMAP to **2** to form **2·DMAP**. This can be attributed to population of the Al 3p orbital by the Lewis base, resulting in decreased Ni 3d → Al 3p back‐bonding (Table S11).

A more detailed MO description of the bonding could be obtained from ETS‐NOCV analysis, which identified a total orbital contribution to the interaction of Δ*E*_orb_ = −73.2 kcal mol^−1^ (Figures 3c and S64; Table S14). The bonding in **2** is dominated by donation of electron density from the two Al─H σ‐bonds to the vacant Ni 3d_yz_ orbital, with significant back‐donation from the filled Ni 3d_xy_ and 3d_z2_ orbitals to a combination of s, p, and d acceptor orbitals on Al as well as the Al─H σ* antibonding orbitals. Agreeing with the Mayer population analysis, the coordination of DMAP results in consistently lower Al contributions to the analogous orbital interactions for **2·DMAP** (Figure S65, Table S15).

### C(sp^2^)─H Activation of DMAP and Pyridine

**Figure 4:**
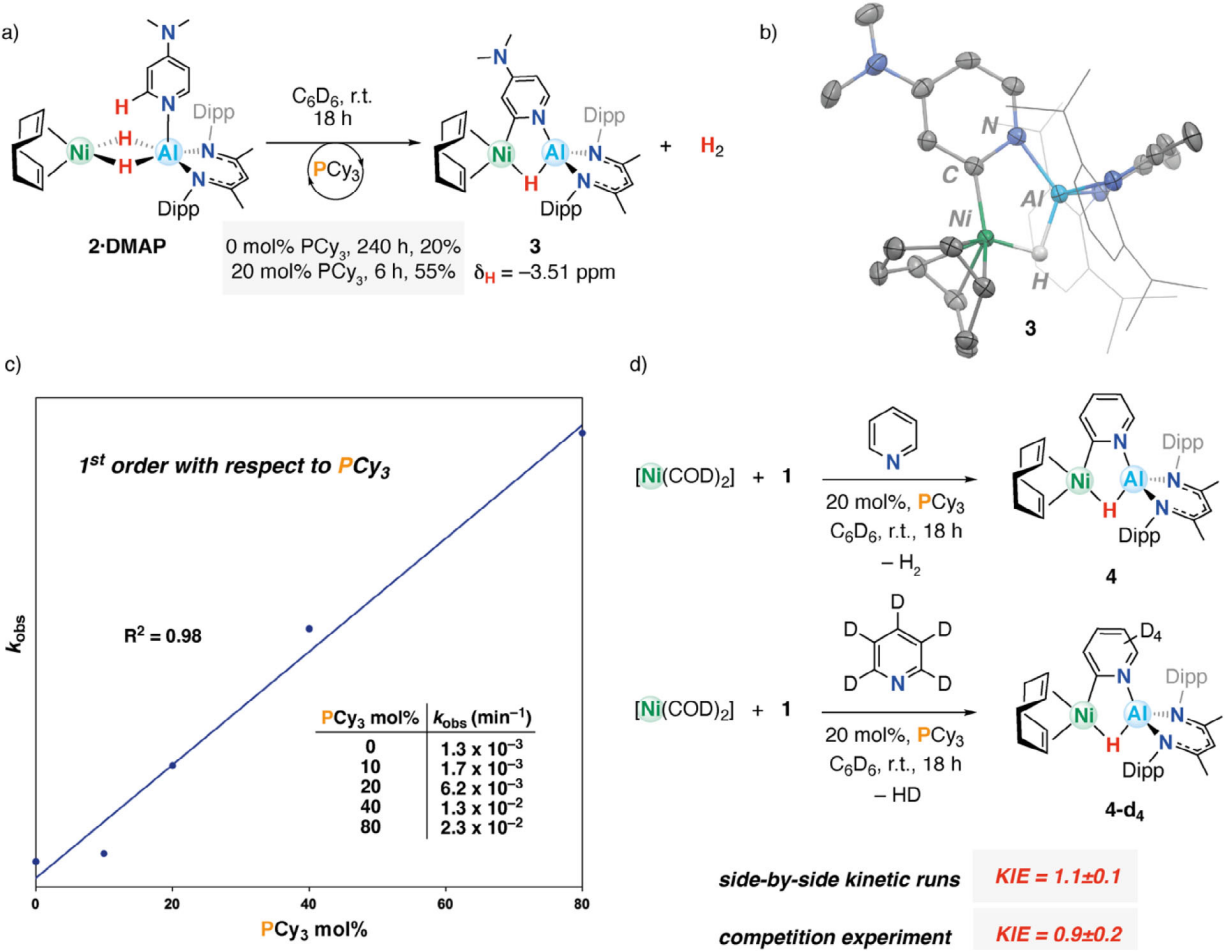
a) Formation of **3** from **2·DMAP**. b) Solid‐state structure of **3**: ellipsoids are drawn at 50% probability, Dipp groups have been truncated for clarity, and all hydrogens except the hydride have been removed. Selected distances (Å): Ni─Al = 2.3189(8), Ni─H = 1.53(2), Al─H = 1.65(2), Ni─C = 1.945(2), C─N = 1.395(3), N─Al = 1.882(2). c) Plot showing 1st‐order dependence of *k*_obs_ on [PCy_3_]. d) Kinetic isotope effect for the C2(sp^2^)─H activation of pyridine to form **4** and **4‐*d*_4_**.

Given the newfound access to **2·DMAP**, we wondered whether the coordination event might bring about the possibility of substrate C(sp^2^)─H bond activation. A C_6_D_6_ solution of **2·DMAP** was monitored overnight at room temperature by ^1^H NMR spectroscopy and showed partial consumption of **2·DMAP** (Figure S18). During this process the aromatic signals of DMAP are desymmetrized, with diagnostic signals appearing for the product at *δ*_H_ = 7.39 and 5.82 ppm, each integrating for 1H along with a third resonance between *δ*_H_ = 7.17–7.21 overlapping with the β‐diketiminate ligand environments. At the same time, the hydride signal of **2·DMAP** at *δ*_H_ = −4.59 ppm was observed to disappear with the appearance of a new resonance at *δ*_H_ = −3.51 ppm. Collectively, these data support a room temperature C(sp^2^)─H activation process at the 2‐position to give **3** (Figure 4a). **3** can be isolated in 61% yield and forms with the elimination of 1 equiv. of H_2_.

The connectivity of **3** was confirmed by X‐ray diffraction (Figure 4b). Complex **3** features a five‐membered cyclic core with a hydride and heteroaryl ligand that bridge Ni and Al centers. The nitrogen of the now C(sp^2^)─H activated DMAP motif maintains coordination to aluminum. Comparatively, the Ni─Al distance of 2.3189(8) Å is longer than those in **2** or **2·DMAP**. The Al─N distance to the pyridyl ligand is 1.882(2) Å in **3**. This is shorter than that of 2.038(3) Å observed in **2·DMAP**, consistent with conversion from a neutral to anionic ligand. At the same time, the C─N distance to the metalated 2‐position of the ring becomes longer on C(sp^2^)─H activation.^[^ ^40^ ^]^

The conversion of **2·DMAP** to **3** occurs slowly with first‐order kinetics at room temperature (*k*_obs_ = 1.3 x 10^−3^ min^−1^). This reaction can, however, be accelerated by the addition of a catalytic quantity of an exogenous ligand. Addition of 20 mol% PCy_3_ to **2·DMAP** led to rapid conversion to **3** with a near fourfold increase in rate reaction (*k*_obs_ = 6.2 x 10^−3^ min^−1^) and improved yields. The kinetics of this transformation were studied. Varying the concentration of PCy_3_ revealed a linear relation (*R* ^2^ = 0.98) between *k*_obs_ and [PCy_3_], indicating a first‐order process. The catalytic effect of PCy_3_ is irrefutable, as in its absence, mixtures of [Ni(COD)_2_], DMAP, and **1** produce at maximum 20% of **3** after 240 h (Figure 4c) at 298 K (*c.f*. 55% of **3** using 20 mol% PCy_3_ after 6 h, as measured by ^1^H NMR spectroscopy). Despite its impact on the rate and efficiency of C(sp^2^)─H activation, there is no evidence for the formation of stable coordination complexes from the binding of PCy_3_ to **2·DMAP** or **3** at 298 K. The C─H activation of DMAP also appears to be reversible: addition of H_2_ (1 bar) to a solution of **3** showed the reformation of the starting materials (Figure S24).

Attempts to expand this reactivity to an array of pyridine‐based substrates provided variable outcomes. No reaction was observed between **1**, [Ni(COD)_2_], and 2,6‐lutidine, either in the presence or absence of PCy_3_ (Figure S22). Quinoline underwent direct hydroalumination and dearomatization with **1** catalyzed by 10 mol% [Ni(COD)_2_]. Pyridine itself proved more successful. While combining 1 equiv. of [Ni(COD)_2_], **1**, and pyridine in C_6_D_6_ showed no signs of binding or C(sp^2^)─H activation by ^1^H NMR spectroscopy, addition of PCy_3_ (20 mol%) to this solution produced **4** in 78% conversion over 18 h (Figure S23). These results further support an important role of PCy_3_ as a catalyst for C(sp^2^)─H bond activation in the absence of a strong *para*‐electron donating group such as ─NMe_2_.

To gain more insight into the mechanism of C(sp^2^)─H activation, the reaction was performed with pyridine‐*d*_5_. A kinetic isotope effect (KIE) of 1.1 ± 0.1 was found at 298 K through side‐by‐side kinetic runs (Figure 4d). A similar KIE of 0.9 ± 0.2 was determined through a competition experiment in which a mixture of 10 equiv. of pyridine and 10 equiv. of pyridine‐d_5_ was added to [Ni(COD)_2_] and **1**. These values indicate either no or a very small KIE, strongly suggesting that the rate‐determining step is not the cleavage of the C(sp^2^)─H bond. For comparison, reactions involving oxidative addition of C(sp^2^)─H bonds to transition metals can occur with KIEs of up to 7–8.^[^ ^41^ ^]^ Activation of pyridine by an Fe–Al heterometallic complex was found to proceed with a very large primary KIE of 14.0 ± 0.2.^[^ ^10^ ^]^ Furthermore, when considering the fate of the isotopic label, during the reaction of pyridine‐d_5_, [Ni(COD)_2_], and **1**, no D‐incorporation was observed in the bridging hydride position of the product **4**, suggesting the isotopic label is lost exclusively through elimination of H–D.

**Figure 5:**
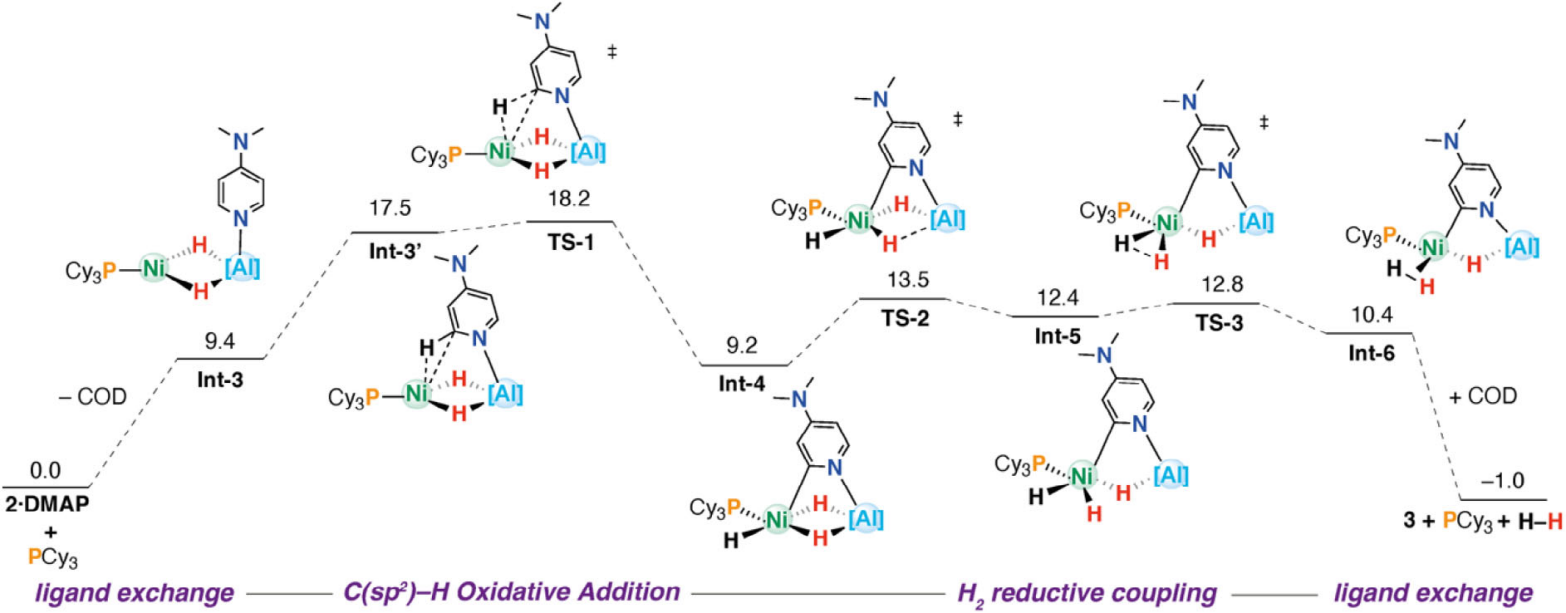
Computed PES for the C–H activation of **2·DMAP** catalyzed by PCy_3_ at the TPSS‐D4/‌def2‐QZVPP/SMD(toluene)//r^2^SCAN‐3c/CPCM(toluene) level of theory. Gibbs energies in kcal mol^−1^. [Al] = Al(BDI).

The conversion of **2⋅DMAP** to **3** provides a rare example of a defined reaction in which both C(sp^2^)─H activation and H_2_ elimination are observed at a heterometallic complex. We thus set out to gain deeper insight into the mechanism of phosphine‐catalyzed C(sp^2^)─H activation and H_2_ elimination by computational means; for clarity, some intermediates in the steps that exchange COD to PCy_3_ have been omitted but are all energetically accessible. (Figure 5 and S42).

The COD to PCy_3_ ligand exchange process at **2·DMAP** was calculated to occur through a stepwise pathway. It is initiated by decoordination of one of the *η* ^2^‐coordinated alkene arms of COD, transforming it from a bidentate to monodentate ligand in **Int**‐**1**. This was calculated to be an uphill but thermodynamically accessible process (Δ*G*°_298K_ = +10.8 kcal mol^−1^). Subsequent coordination of PCy_3_ forms **Int‐2** (Δ*G*°_298 K_ = +13.6 kcal mol^−1^) followed by the dissociation of COD, generating key intermediate **Int‐3** (Δ*G*°_298 K_ = +9.4 kcal mol^−1^). C(sp^2^)─H activation proceeds from an active conformer of **Int‐3**, **Int‐3′** (Δ*G*°_298 K_ = +17.5 kcal mol^−1^), which is stabilized through an *η* ^2^‐C─H agostic interaction with Ni. This leads directly to the C─H bond‐breaking transition state **TS‐1** (Δ*G* ^‡^_298K_ = +18.2 kcal mol^−1^), yielding the oxidative addition product **Int‐4**, which features a Ni─C(sp^2^) bond and a terminal hydride ligand on Ni, in addition to the two hydrides bridging Ni and Al centers. The reductive elimination of dihydrogen from **Int‐4** is a multistep process, initiated by migration of the bridging hydride ligand (**TS‐2**, Δ*G* ^‡^_298 K_ = +13.5 kcal mol^−1^) to a terminal position to form **Int‐5** (Δ*G*°_298 K_ = +12.4 kcal mol^−1^). Reductive coupling of H_2_ occurs through **TS‐3** (Δ*G* ^‡^_298K_ = +12.8 kcal mol^−1^) with formation of the dihydrogen complex **Int‐6** (Δ*G*°_298 K_ = +10.4 kcal mol^−1^). The dissociation of H_2_ and ligand exchange of PCy_3_ for COD completes the reaction sequence, regenerating the phosphine, the product **3**, and a molecule of H_2_ (Δ*G*°_298 K_ = −1.0 kcal mol^−1^).

NBO calculations were performed to gain insight into the changes in electronic structure across the reaction pathway and the role of phosphine in catalyzing the C─H activation process. The C─H activation of DMAP gave more positive charge on the two metals as the reaction progressed, consistent with a formal oxidation from Ni(0)/Al(III) to Ni(II)/Al(III). Specifically for the series from **Int‐3** → **Int‐3′** → **TS‐1** → **Int‐4** the NPA charges on Ni trend −0.11 → +0.13 → +0.17 → +0.19, while those on Al trend +1.45 → +1.52 → +1.59, →+1.61.

To support the catalytic role of PCy_3_, alternative C─H activation mechanisms ligated only by COD were also explored (Figures S43–S51). The most accessible phosphine‐free transition state for the C─H activation of DMAP is a direct analogue of **TS‐1**, supported by an *η* ^2^‐COD ligand (**TS‐1a**, Δ*G* ^‡^_298 K_ = +30.2 kcal mol^−1^). Comparing the electronic structure of the key transition states **TS‐1** to **TS‐1a** suggests that PCy_3_ likely accelerates the C─H activation step through increasing the electron density at Ni, resulting in increased population of the C─H σ* antibonding orbital of the pyridine fragment, allowing for bond breaking (NPA charge on Ni: **TS‐1**: +0.17; **TS‐1a**: +0.36).^[^ ^42^ ^]^ Alternatively, reactivity might be promoted through access to a three‐coordinate Ni site in **Int‐3**, which is expected to be more reactive than the four‐coordinate center in **2·DMAP**.

The DFT calculations suggest that the oxidative addition of the C(sp^2^)─H bond of DMAP to **1** is a low‐energy process that, when catalyzed by PCy_3_, is facile at room temperature. This observation is not only consistent with the lack of significant KIE in the reaction of **1** with pyridine catalyzed by PCy_3_,^[^ ^41^ ^]^ but also with numerous studies involving two‐component Ni/Al catalyst systems for the C–H functionalization of pyridines, which occur with low KIEs ranging between 1.04 and 1.25.^[^ ^16^, ^19^, ^20^ ^]^ These data suggest that C─H activation in these systems may be a universally low‐energy step, one that is not rate limiting. Similarly, the DFT calculations suggest that reductive elimination of H_2_ *en route* to form **3** proceeds through an almost flat potential energy surface and is also unlikely to be rate‐limiting. This prediction is consistent with the experimental observation that no hydride scrambling between bridging and terminal positions occurs in the reaction of **1**, cat. PCy_3_, and pyridine‐d_5_. Rather, based on the lack of experimental KIE and 1st‐order dependence of kinetics in PCy_3_, we suggest that an activation barrier associated with the COD to PCy_3_ ligand‐exchange sequence is likely rate‐determining.

## Conclusion

In summary, we report a rare example of a Ni–Al heterometallic complex that is capable of the site‐specific C–H activation of 4‐dimethylaminopyridine (or pyridine) at the 2‐position by oxidative addition. This reaction occurs with subsequent H_2_ reductive elimination to maintain the overall oxidation state of the heterometallic complex. Remarkably, the reaction sequence is catalyzed by an exogenous ligand, PCy_3_, which gives faster rates and higher yields of C─H activation. Unprecedented insight is provided into the nature of bond breaking and making at the heterometallic scaffold through a combination of kinetics, KIEs, isotope labeling experiments, and DFT calculations. Throughout the pathway, the Al center plays a key role in anchoring the substrate in place through a Lewis‐acid/Lewis‐base interaction. PCy_3_ catalyzes the oxidative addition step through ligand exchange at the Ni site and stabilization of the key transition state through its strong σ‐donor properties. These findings provide an underpinning framework for recent developments in C─H functionalization catalysis that use combinations of Ni precursors and Lewis‐acidic Al additives. This suggests that C─H activation of pyridine is energetically facile and unlikely to be rate‐limiting, whereas ligand exchange steps leading to the key reactive intermediate play a more significant role in determining the overall reaction rate.

## Supporting Information

Materials and methods, synthetic procedures, NMR and IR spectra of all compounds, kinetics data, the crystal structure of **S1**, crystallographic data, and computational methods and data (PDF). The authors cited additional references in the Supporting Information.^[^ ^42^, ^43^, ^44^, ^45^, ^46^, ^47^, ^48^, ^49^, ^50^, ^51^, ^52^, ^53^, ^54^, ^55^, ^56^, ^57^, ^58^, ^59^, ^60^, ^61^, ^62^, ^63^, ^64^, ^65^, ^66^, ^67^, ^68^, ^69^, ^70^, ^71^, ^72^, ^73^, ^74^, ^75^, ^76^, ^77^, ^78^ ^]^ Cartesian coordinates of the DFT‐optimized structures (XYZ). Crystal structures (cif). CCDC 2417828 (for **2**), 2417829 (for **2·DMAP**), 2417830 (for **3**), 2417831 (for **4**), and 2417832 (for S1) contain the supplementary crystallographic data for this paper. These data are provided free of charge by the joint Cambridge Crystallographic Data Centre and Fachinformationszentrum Karlsruhe Access Structures service via www.ccdc.cam.ac.uk/data_request/cif.

## Author Contributions

J.A.Z. performed all experimental work. B.S. performed all crystallographic and computational work. J.A.Z. and B.S. are listed as joint first authors. M.W.D. and M.R.C. supervised the project. All authors were involved in writing, reviewing, and editing drafts of this paper.

## Conflict of Interests

The authors declare no conflict of interest.

## Supporting information



Supporting Information

Supporting Information

## Data Availability

The data that support the findings of this study are available from the corresponding author upon reasonable request.
