# Supplementary material for: C(sp2)─H Bond Activation with a Heterometallic Nickel–Aluminium Complex
Source: Angew Chem Int Ed Engl. 2025 Jul 23;64(36):e202512684. doi: 10.1002/anie.202512684 (PMC12402840; doi:10.1002/anie.202512684)
Supplement: Supplementary file 1 — Supporting Information [file ANIE-64-e202512684-s001.pdf]

Supporting information for

C(sp²)–H Bond Activation with a Heterometallic Nickel---Aluminium Complex

Joseph A. Zurakowski,[bc‡] Benedek Stadler,[a‡] Marcus W. Drover,[b*] Mark R. Crimmin[a*]

* Corresponding authors, E-mail: marcus.drover@uwo.ca, m.crimmin@imperial.ac.uk

‡ Joint first authors

[a] Department of Chemistry, Imperial College London, Molecular Sciences Research Hub, 82 Wood Lane, Shepherds Bush, London, W12 0BZ, United Kingdom

[b] Department of Chemistry, Western University, 1151 Richmond Street, London, ON, N8K 3G6, Canada

[c] Department of Chemistry and Biochemistry, The University of Windsor, 401 Sunset Avenue, Windsor, ON, N9B 3P4, Canada

S1 Experimental Section....2

S2 Preparation of Compounds....3

S3 NMR and IR Spectroscopic Data....9

S4 Equilibrium Analysis of **1** and **2**....21

S5 Kinetic Data for the C(sp²)–H Activation....24

S6 X-Ray Crystallography....32

S7 Computational details....38

S8 References....94

S1 Experimental Section

S1.1 General considerations

All experiments were carried out employing standard Schlenk techniques under an atmosphere of dry dinitrogen employing degassed, dried solvents. Non-halogenated solvents were tested with a standard purple solution of sodium benzophenone ketyl in tetrahydrofuran in order to confirm effective moisture removal. d_6-Benzene was dried over 3Å molecular sieves and degassed by three freeze-pump-thaw cycles. All other reagents were purchased from commercial vendors and used without further purification unless otherwise stated. **1** was prepared according to a literature procedure.[1] 4-dimethylaminopyridine (DMAP) was purchased from MilliporeSigma Canada Ltd and recrystallized from *n*-pentane at –35 °C overnight.

S1.2 Physical methods

^{1}H NMR spectra are reported in parts per million (ppm) and are referenced to residual solvent e.g., ^{1}H(C_6D_6): δ = 7.16; ^{13}C(C_6D_6): δ = 128.06; coupling constants are reported in Hz. ^{13}C{^{1}H} and ^{31}P{^{1}H} NMR spectra were performed as proton-decoupled experiments (unless explicitly stated otherwise) and are reported in ppm.

S1.3 Bulk purity analysis

In order to determine bulk purity, ^{1}H NMR spectroscopy was performed on the compound of interest with an internal standard of hexamethyldisiloxane (HMDSO). Bulk purity results are included in the experimental section for each compound. The compounds disclosed herein were found to rapidly decompose upon contact with air/moisture, precluding elemental analysis as a reliable technique to assess bulk purity.[2,3]

S2 Preparation of Compounds

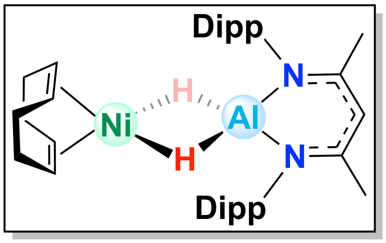

[(COD)Ni{(μ-H)$_2$Al(BDI)}] (**2**; $C_{37}H_{55}AlN_2Ni$, M_W = 614 g/mol): In the glovebox, [Ni(COD)$_2$] (150 mg, 0.55 mmol) was weighed into a 20 mL scintillation vial equipped with a stir bar and dissolved in approximately 5 mL of toluene. Next, a solution of **1** (243 mg, 0.55 mmol) in 1 mL of toluene was added. The reaction was stirred for 18 h at room temperature during which the solution turned from yellow to red. The reaction mixture was then evaporated to dryness *in-vacuo*. 1 mL of toluene was added to dissolve the solid material. The reaction mixture was subsequently evaporated to dryness *in-vacuo*. This process was repeated 10 times to ensure that all the COD was removed. The solids became increasingly red during this process. Finally, 1 mL of *n*-pentane was added and removed *in-vacuo*. Once the solid was dry, *n*-pentane was added until all of the solid material was dissolved (*ca.* 5 mL of *n*-pentane). The scintillation vial was placed in the freezer at –35 °C overnight. Unreacted [Ni(COD)$_2$] recrystallized from this solution as yellow blocks. The red supernatant was separated, reduced to half of its original volume, and placed back into the freezer at –35 °C. **2** recrystallized as red blocks from this solution overnight (44 mg, 10 %). These crystals were isolated and found to be suitable for analysis by single-crystal X-ray diffraction. Quantitative ^{1}H NMR spectroscopy on a bulk sample of **2** gave a purity of 65%; [Ni(COD)$_2$] consistently co-crystallized with **2**. **^{1}H NMR (400 MHz, C_6D_6, 298 K):** δ_H = 7.13-7.09 (m; 6H; (Dipp)-C$\underline{H}_{aromatic}$), 4.89 (s; 1H; (BDI)-C$\underline{H}$), 4.08 (br. s; 4H; (COD)-C$\underline{H}_{alkene}$), 3.15 (br. sept; 4H; (^{i}Pr)-C$\underline{H}$), 2.29 (br. m; 4H; (COD)-C$\underline{H}_{alkane}$), 2.13 (br. m; 4H; (COD)-C$\underline{H}_{alkane}$), 1.58-1.55 (18H; overlapping (BDI)-C$\underline{H}_3$ and (^{i}Pr)-C$\underline{H}_3$ signals), 1.08 (d; 12H; (^{i}Pr)-C$\underline{H}_3$), –3.27 (br.; 2H; [Ni-(μ-$\underline{H}$)$_2$-Al]). **^{13}C{^{1}H} NMR (125.8 MHz, C_6D_6, 298 K):** δ_C = 170.9 ((BDI)-$\underline{C}$N), 143.8 ((Dipp)-$\underline{C}$N), 138.7 ((Dipp)-$\underline{C}$-(^{i}Pr)), 124.7 ($\underline{C}$-H$_{aromatic}$), 97.5 ((BDI)-$\underline{C}$H), 78.2 ((COD)-$\underline{C}$H$_{alkene}$), 32.0 ((COD)-$\underline{C}$H$_{alkane}$), 29.1 ((^{i}Pr)-$\underline{C}$H), 24.9 (multiple overlapping $\underline{C}$H$_3$ signals), 24.3 (multiple overlapping $\underline{C}$H$_3$ signals).

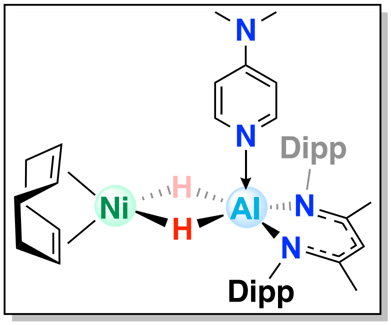

[(COD)Ni{(μ-H)$_2$(DMAP)Al(BDI)}] (**2·DMAP**; $C_{44}H_{65}AlN_4Ni$, M_W = 736 g/mol): In the glovebox, [Ni(COD)$_2$] (200 mg, 0.73 mmol) was weighed into a 20 mL scintillation vial equipped with a stir bar and dissolved in approximately 3 mL of toluene. Next, a solution of **1** (324 mg, 0.73 mmol) and DMAP (89 mg, 0.73 mmol) in 1 mL of toluene was added. The reaction was stirred for 18 h at room temperature during which the solution turned dark red. The reaction mixture was then evaporated to dryness *in-vacuo*. 1 mL of toluene was added and removed *in-vacuo*. This process was repeated 5 times to ensure that all the COD was removed. The resulting red solid was dissolved in 3 mL of toluene, filtered through a 1 cm^3 plug of Celite®, and placed in the freezer at –35 °C. Dark red crystals of **2·DMAP** suitable for X-ray diffraction were formed overnight (396 mg, 0.54 mmol, 74 %). Quantitative ^{1}H NMR spectroscopy on a bulk sample of **2·DMAP** gave a purity of 90%. **^{1}H NMR (400 MHz, C_6D_6, 298 K):** δ_H = 8.88 (d; 2H; (DMAP)-C<u>H</u>$_{ortho}$ ($^3J_{H\text{-}H}$ = 5.81 Hz)), 7.21-7.18 (m; 2H; (Dipp)-C<u>H</u>$_{aromatic}$), 7.14-7.13 (m; 4H; (Dipp)-C<u>H</u>$_{aromatic}$), 5.94 (d; 2H; (DMAP)-C<u>H</u>$_{meta}$), 5.00 (s; 1H; (BDI)-C<u>H</u>), 4.19 (br. m; 4H; (COD)-C<u>H</u>$_{alkene}$), 3.17 (sept.; 4H; (^{i}Pr)-C<u>H</u> ($^3J_{H\text{-}H}$ = 6.84 Hz)), 2.44 (m; 4H; (COD)-C<u>H</u>$_{alkane}$), 2.31 (m; 4H; (COD)-C<u>H</u>$_{alkane}$), 2.08 (s; 6H; (DMAP)-N(C<u>H</u>$_3$)$_2$), 1.63 (s; 6H; (BDI)-C<u>H</u>$_3$), 1.40 (d; 12H; (^{i}Pr)-C<u>H</u>$_3$ ($^3J_{H\text{-}H}$ = 6.84 Hz)), 1.12 (d; 12H; (^{i}Pr)-C<u>H</u>$_3$ ($^3J_{H\text{-}H}$ = 6.84 Hz)), –4.59 (br.; 2H; [Ni-(μ-<u>H</u>)$_2$-Al]). **^{13}C{^{1}H} NMR (100.6 MHz, C_6D_6, 298 K):** δ_C = 170.4 ((BDI)-<u>C</u>N), 150.3 ((DMAP)-<u>C</u>H$_{ortho}$), 143.8 ((Dipp)-<u>C</u>N), 129.3 (<u>C</u>H$_{aromatic}$), 129.1 (<u>C</u>H$_{aromatic}$), 127.2 ((Dipp)-<u>C</u>-(^{i}Pr)), 124.3 (<u>C</u>H$_{aromatic}$), 105.9 ((DMAP)-<u>C</u>H$_{meta}$), 97.5 ((BDI)-<u>C</u>H), 89.7 ((DMAP)-<u>C</u>N(CH$_3$)$_2$), 75.1 ((COD)-<u>C</u>H$_{alkene}$), 38.3 ((DMAP)-N(<u>C</u>H$_3$)$_2$), 32.8 ((COD)-<u>C</u>H$_{alkane}$), 29.1 ((^{i}Pr)-<u>C</u>H), 24.9-24.8 (overlapping (BDI)-<u>C</u>H$_3$ and (^{i}Pr)-<u>C</u>H$_3$ signals).

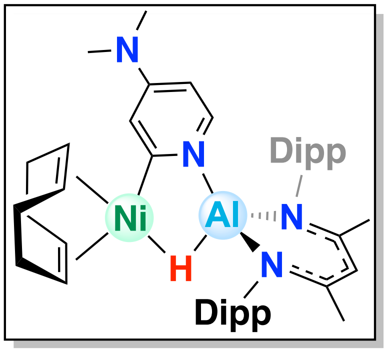

[(COD)Ni(μ-H)(μ-[C_5H_3N]NMe_2)Al(BDI)] (**3**; $C_{44}H_{63}AlN_4Ni$, M_W = 734 g/mol): Method 1: In the glovebox, [Ni(COD)$_2$] (30 mg, 0.11 mmol) was weighed into a 20 mL scintillation vial equipped with a stir bar and dissolved in approximately 3 mL of toluene. Next, a solution of **1** (49 mg, 0.11 mmol) and DMAP (13 mg, 0.11 mmol) in 3 mL of toluene was added. After stirring the reaction mixture for 10 min, 20 mol% of PCy_3 (6.1 mg, 0.02 mmol) was added as a toluene solution (*ca.* 500 μL). The reaction was stirred for 18 h at room temperature, during which the solution turned orange. The reaction mixture was evaporated to dryness *in-vacuo*. The orange oil was washed with 3 x 5 mL of cold (–35 °C) *n*-pentane to remove all of the PCy_3. **3** was obtained after recrystallizing from a saturated toluene/*n*-pentane mixture at –35°C overnight (49 mg, 0.07 mmol, 61 %). Method 2: **2·DMAP** (20 mg, 0.02 mmol) was dissolved in 1 mL of toluene and 20 mol% of PCy_3 was added from a stock solution in toluene (0.20 M, 28 μL). The reaction mixture was stirred for 18 h at room temperature, during which the solution turned orange. The reaction mixture was evaporated to dryness *in-vacuo*. 1 mL of *n*-pentane was added, and the orange solution filtered through a 1 cm^3 plug of Celite®. Crystals of **3** suitable for X-ray diffraction were grown from this *n*-pentane solution at –35 °C overnight (8 mg, 0.01 mmol, 40%). Quantitative ^{1}H NMR spectroscopy on a bulk sample of **3** gave a purity of 87%. **^{1}H NMR (400 MHz, C_6D_6, 298 K):** δ_H = 7.39 (d; 1H; (DMAP)-C$\underline{H}_{aromatic}$ ($^3J_{H-H}$ = 6.61 Hz)), 7.21-7.17 (m; 3H; overlapping (Dipp)-C$\underline{H}_{aromatic}$ and (DMAP)CH$_{aromatic}$), 7.14-7.10 (m; 2H; (Dipp)-C$\underline{H}_{aromatic}$), 7.03-7.00 (m; 2H; (Dipp)-C$\underline{H}_{aromatic}$), 5.82 (dd; 1H; (DMAP)-C$\underline{H}_{aromatic}$ ($^3J_{H-H}$ = 6.61 Hz; $^3J_{H-H}$ = 2.78 Hz)), 4.96 (s; 1H; (BDI)-C$\underline{H}$), 4.07 (m; 2H; (COD)-C$\underline{H}_{alkene}$), 3.84 (m; 2H; (COD)-C$\underline{H}_{alkene}$), 3.71 (sept.; 2H; (^{i}Pr)-C$\underline{H}$ ($^3J_{H-H}$ = 6.86 Hz)), 2.90 (sept.; 2H; (^{i}Pr)-C$\underline{H}$ ($^3J_{H-H}$ = 6.73 Hz)), 2.69 (m; 2H; (COD)-C$\underline{H}_{alkane}$), 2.41 (m; 2H; (COD)-C$\underline{H}_{alkane}$), 2.39 (s; 6H; (DMAP)-N(C$\underline{H}_3$)$_2$), 2.33 (m; 2H; (COD)-C$\underline{H}_{alkane}$), 2.25 (m; 2H; (COD)-C$\underline{H}_{alkane}$), 1.67 (d; 6H; (^{i}Pr)-C$\underline{H}_3$ ($^3J_{H-H}$ = 6.86 Hz)), 1.65 (s; 6H; (BDI)-C$\underline{H}_3$), 1.19 (d; 6H; (^{i}Pr)-C$\underline{H}_3$ ($^3J_{H-H}$ = 6.86 Hz)), 1.16 (d; 6H; (^{i}Pr)-C$\underline{H}_3$ ($^3J_{H-H}$ = 6.73 Hz)), 0.84 (d; 6H; (^{i}Pr)-C$\underline{H}_3$ ($^3J_{H-H}$ = 6.73 Hz)), –3.51 (br.; 1H; [Ni-(μ-$\underline{H}$)-Al]). **^{13}C{^{1}H} NMR (151.0 MHz, C_6D_6, 298 K):** δ_C = 214.6 ([Ni]-$\underline{C}_{DMAP}$), 170.5 ((BDI)-$\underline{C}$N), 151.3 ((DMAP)-$\underline{C}$N), 144.8 ((BDI)-$\underline{C}_{aromatic}$), 143.2 ((BDI)-$\underline{C}_{aromatic}$), 141.0 ((BDI)-$\underline{C}_{aromatic}$), 140.3 ((DMAP)-$\underline{C}$H$_{aromatic}$), 127.3 ((BDI)-$\underline{C}$H$_{aromatic}$), 124.4 ((BDI)-$\underline{C}$H$_{aromatic}$), 124.1 ((BDI)-

$\underline{C}H_{aromatic}$), 117.7 ((DMAP)-C$\underline{H}_{aromatic}$), 100.8 ((DMAP)-$\underline{C}H_{aromatic}$), 97.2 ((BDI)-$\underline{C}$H), 82.9 ((COD)-$\underline{C}H_{alkene}$), 77.0 ((COD)-$\underline{C}H_{alkene}$), 38.4 ((DMAP)-N($\underline{C}H_3)_2$), 33.6 ((COD)-$\underline{C}H_{alkane}$), 30.9 ((COD)-$\underline{C}H_{alkane}$), 29.7 ((^{i}Pr)-$\underline{C}$H), 28.7 ((^{i}Pr)-$\underline{C}$H), 25.0 ((^{i}Pr)-$\underline{C}H_3$), 24.7 ((^{i}Pr)-$\underline{C}H_3$), 24.5 ((^{i}Pr)-$\underline{C}H_3$), 24.3 ((^{i}Pr)-$\underline{C}H_3$), 23.7 ((BDI)-$\underline{C}H_3$).

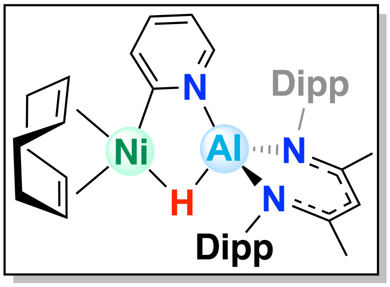

[(COD)Ni(μ-H)(μ-[C_5H_3N])Al(BDI)] (**4**; $C_{42}H_{58}AlN_3Ni$, M_W = 691 g/mol): In the glovebox, [Ni(COD)$_2$] (30 mg, 0.11 mmol) was weighed into a 20 mL scintillation vial equipped with a stir bar and dissolved in approximately 3 mL of toluene. Next, a solution of **1** (49 mg, 0.11 mmol) and pyridine (26 μL, 0.33 mmol) in 3 mL of toluene was added. After stirring the reaction mixture for 10 min, 20 mol% of PCy_3 (6.1 mg, 0.02 mmol) was added as a toluene solution (*ca.* 500 μL). The reaction was stirred for 18 h at room temperature. The solvent was removed *in-vacuo* and the resulting dark red powder was extracted into 5 mL of *n*-pentane and filtered through a 1 cm^3 plug of Celite®. The solvent was removed *in-vacuo*. The resulting powder was analyzed by ^{1}H NMR spectroscopy, giving a yield for the titled compound of 78%. Recrystallization of **4** was performed at –35 °C overnight. **4** could not be separated from [Ni(COD)$_2$] despite multiple attempts at recrystallization. Manually separating red crystals of **4** from yellow crystals of [Ni(COD)$_2$] gave small samples of **4** (~10 mg each) suitable for analysis by ^{1}H/^{13}C NMR spectroscopy and single-crystal X-ray diffraction. Quantitative ^{1}H NMR spectroscopy on a sample of **4** gave a purity of 66%. **^{1}H NMR (600 MHz, C_6D_6, 298 K):** δ_H = 7.77 (br. s; 1H; (pyridine)-C$\underline{H}_{aromatic}$), 7.32 (br. s; 1H; (pyridine)-C$\underline{H}_{aromatic}$), 7.16-7.00 (6H; overlapping (Dipp)-C$\underline{H}_{aromatic}$ signals), 6.67 (br. s; 1H; (pyridine)-C$\underline{H}_{aromatic}$), 6.10 (br. s; 1H; (pyridine)-C$\underline{H}_{aromatic}$), 4.91 (br. s; 1H; (BDI)-C$\underline{H}$), 4.04 (br. m; 2H; (COD)-C$\underline{H}_{alkene}$), 3.75 (br. m; 2H; (COD)-C$\underline{H}_{alkene}$), 3.64 (br. m; 2H; (^{i}Pr)-C$\underline{H}$), 2.71 (br. m; 2H; (^{i}Pr)-C$\underline{H}$), 2.56 (br. m; 2H; (COD)-C$\underline{H}_{alkane}$), 2.29-2.15 (6H; overlapping (COD)-C$\underline{H}_{alkane}$ signals), 1.60 (12H; overlapping (^{i}Pr)-C$\underline{H}_3$ and (BDI)-C$\underline{H}_3$ signals), 1.15-1.10 (12H; overlapping (^{i}Pr)-C$\underline{H}_3$ signals), 0.71 (br. m; 6H; (^{i}Pr)-C$\underline{H}_3$), –3.17 (br.; 1H; [Ni-(μ-$\underline{H}$)-Al]). **^{13}C{^{1}H} NMR (151.0 MHz, C_6D_6, 298 K):** δ_C = 219.7 ([Ni]-$\underline{C}$; assigned using a ^{1}H-^{13}C HMBC NMR spectroscopy experiment), 171.0 ((BDI)-$\underline{C}$N), 144.7 ((BDI)-$\underline{C}_{aromatic}$), 143.1 ((BDI)-$\underline{C}_{aromatic}$), 140.6 ((BDI)-$\underline{C}_{aromatic}$), 140.5 ((pyridine)-$\underline{C}H_{aromatic}$), 138.5 ((pyridine)-$\underline{C}H_{aromatic}$), 132.3 ((pyridine)-$\underline{C}H_{aromatic}$), 127.4 ((BDI)-$\underline{C}H_{aromatic}$), 124.5 ((BDI)-C$\underline{H}_{aromatic}$), 124.1 ((BDI)-$\underline{C}H_{aromatic}$), 113.1 ((pyridine)-$\underline{C}$H), 97.1 ((BDI)-$\underline{C}$H), 84.3 ((COD)-$\underline{C}H_{alkene}$), 78.0 ((COD)-$\underline{C}H_{alkene}$), 33.3 ((COD)-$\underline{C}H_{alkane}$), 30.7 ((^{i}Pr)-$\underline{C}$H), 29.7 ((^{i}Pr)-$\underline{C}$H), 28.6 ((^{i}Pr)-$\underline{C}H_3$), 24.9 ((^{i}Pr)-$\underline{C}H_3$), 24.5 ((^{i}Pr)-$\underline{C}H_3$), 24.4 ((^{i}Pr)-$\underline{C}H_3$), 23.8 ((BDI)-$\underline{C}H_3$).

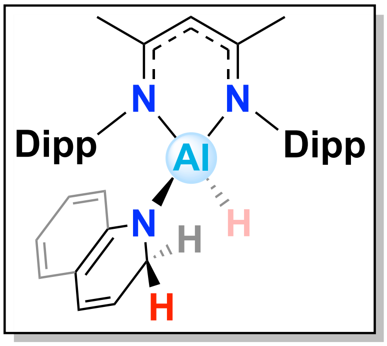

[(H)(2-hydroquinoline)Al(BDI)] (**S1**; $C_{38}H_{50}AlN_3$, M_W = 576 g/mol): In the glovebox, **1** (43 mg, 0.10 mmol) was weighed into a 20 mL scintillation vial equipped with a stir bar and dissolved in approximately 3 mL of C_6H_6. Next, quinoline (11.4 µL, 0.10 mmol) was added. This mixture was stirred for 5 min until all of **1** had dissolved. The mixture of **1** and quinoline was added to a stirring solution of 10 mol% [Ni(COD)$_2$] (3 mg, 0.01 mmol) in 1 mL of C_6H_6. The reaction was stirred for 4 h at room temperature, during which the solution became a dark green. The solvent was removed *in-vacuo* to give a dark green powder, which was dissolved in 3 mL of *n*-pentane and filtered through a 1 cm^3 plug of Celite®. Crystals of **5** suitable for X-ray diffraction were grown from a minimum solution of *n*-pentane at –35 °C overnight (32 mg, 0.06 mmol, 56 %). Quantitative ^{1}H NMR spectroscopy on a bulk sample of **5** gave a purity of 91%. **^{1}H NMR (400 MHz, C_6D_6, 298 K):** δ_H = 7.20-7.17 (3H; multiple overlapping (Dipp)-C$\underline{H}_{aromatic}$ and (2-hydroquinoline)-C$\underline{H}_{aromatic}$ signals), 7.14-7.04 (4H; multiple overlapping (Dipp)-C$\underline{H}_{aromatic}$ and (2-hydroquinoline)-C$\underline{H}_{aromatic}$ signals), 6.83 (m; 1H; (2-hydroquinoline)-C$\underline{H}_{aromatic}$), 6.65 (m; 1H; (2-hydroquinoline)-C$\underline{H}_{aromatic}$), 6.40 (m; 1H; (2-hydroquinoline)-C$\underline{H}_{aromatic}$), 6.12 (m; 1H; (2-hydroquinoline)-C$\underline{H}_{alkene}$), 5.13 (s; 1H; (BDI)-C$\underline{H}$), 5.04 (m; 1H; (2-hydroquinoline)-C$\underline{H}_{alkene}$), 4.22 (m; 2H; (2-hydroquinoline)-C$\underline{H}_2$), 3.41 (sept.; 2H; (^{i}Pr)-C$\underline{H}$ ($^3J_{H\text{-}H}$ = 6.76 Hz)), 3.30 (sept.; 2H; (^{i}Pr)-C$\underline{H}$ ($^3J_{H\text{-}H}$ = 6.76 Hz)), 1.58 (s; 6H; (BDI)-C$\underline{H}_3$), 1.49 (d; 6H; (^{i}Pr)-C$\underline{H}_3$ ($^3J_{H\text{-}H}$ = 6.76 Hz)), 1.24 (d; 6H; (^{i}Pr)-C$\underline{H}_3$ ($^3J_{H\text{-}H}$ = 6.76 Hz)), 1.17 (d; 6H; (^{i}Pr)-C$\underline{H}_3$ ($^3J_{H\text{-}H}$ = 6.76 Hz)), 1.09 (d; 6H; (^{i}Pr)-C$\underline{H}_3$ ($^3J_{H\text{-}H}$ = 6.76 Hz)). The [Al-$\underline{H}$] peak was not located due to the quadrupolar nature of the I = +5/2 ^{27}Al nucleus. **^{13}C{^{1}H} NMR (100.6 MHz, C_6D_6, 298 K):** δ_C = 170.8 ((BDI)-$\underline{C}$N), 150.1 ((2-hydroquinoline)-$\underline{C}$N), 146.5 ((Dipp)-$\underline{C}$N), 143.7 ((Dipp)-$\underline{C}$-(^{i}Pr), 140.0 ((Dipp)-$\underline{C}$N), 127.6 ($\underline{C}H_{aromatic}$), 127.2 ($\underline{C}H_{aromatic}$), 125.1 ((2-hydroquinoline)-$\underline{C}$), 124.5 ($\underline{C}H_{aromatic}$), 121.0 ((2-hydroquinoline)-$\underline{C}H_{alkene}$), 116.7 ($\underline{C}H_{aromatic}$), 115.7 ($\underline{C}H_{aromatic}$), 97.8 ((BDI)-$\underline{C}$H), 47.4 ((2-hydroquinoline)-$\underline{C}H_2$), 29.1 ((^{i}Pr)-$\underline{C}$H), 28.1 ((^{i}Pr)-$\underline{C}$H), 25.3 ((^{i}Pr)-$\underline{C}H_3$), 24.9 ((^{i}Pr)-$\underline{C}H_3$), 24.6 ((^{i}Pr)-$\underline{C}H_3$), 23.4 ((BDI)-$\underline{C}H_3$). **FT-IR (ATR):** 1847 cm^{-1} (ν[Al-$\underline{H}$]).

S3 NMR and IR Spectroscopic Data

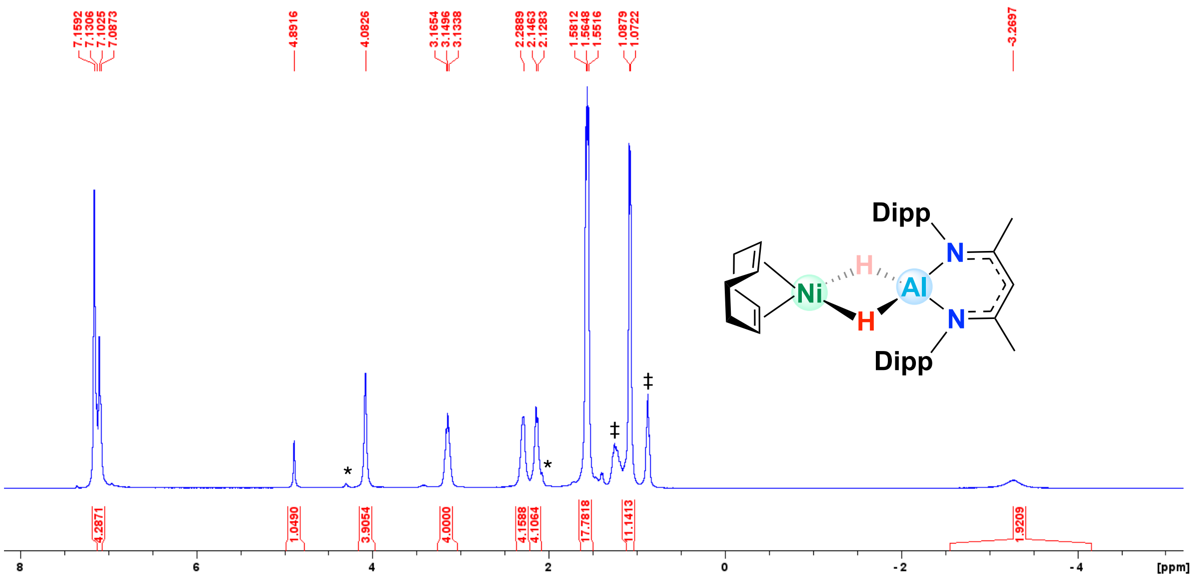

Figure S1 ^{1}H NMR, C_6D_6, 400 MHz, 298 K. * = $[Ni(COD)_2]$. ‡ = residual *n*-pentane.

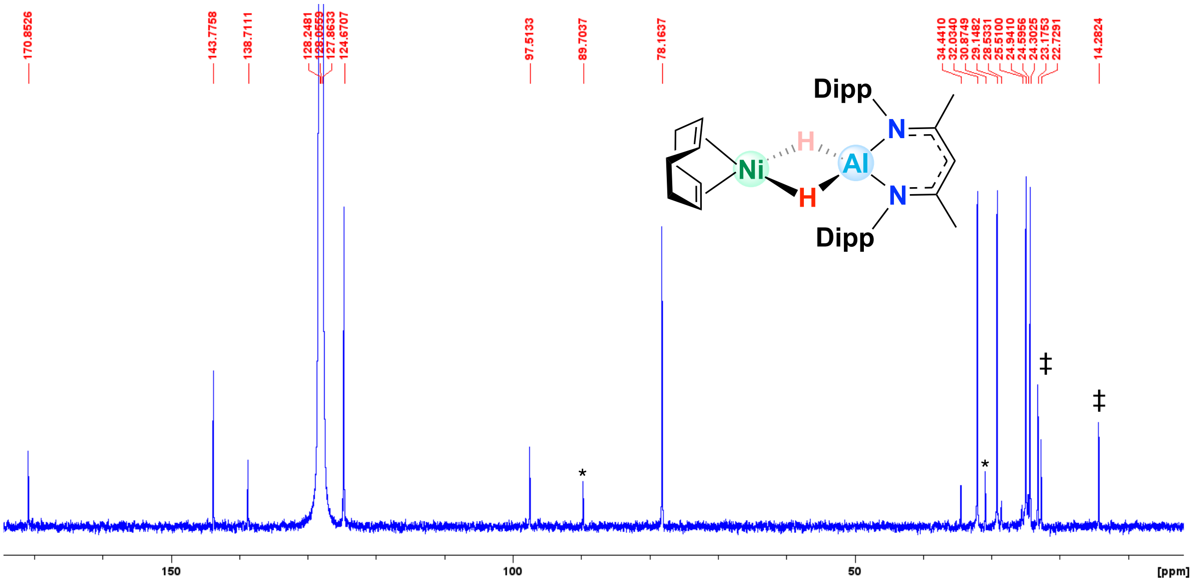

Figure S2 **2**, $^{13}C\{^{1}H\}$ NMR, C_6D_6, 125.8 MHz, 298 K. * = $[Ni(COD)_2]$. ‡ = residual *n*-pentane.

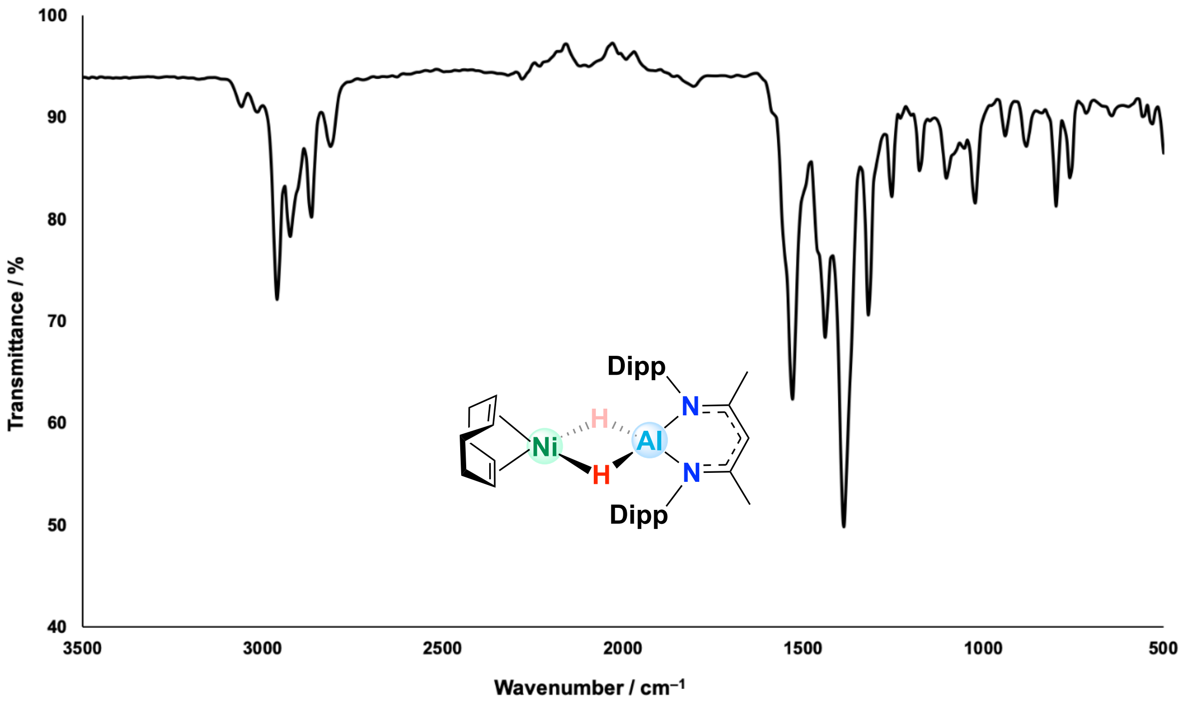

Figure S3 **2**, FT-IR (ATR), 298 K.

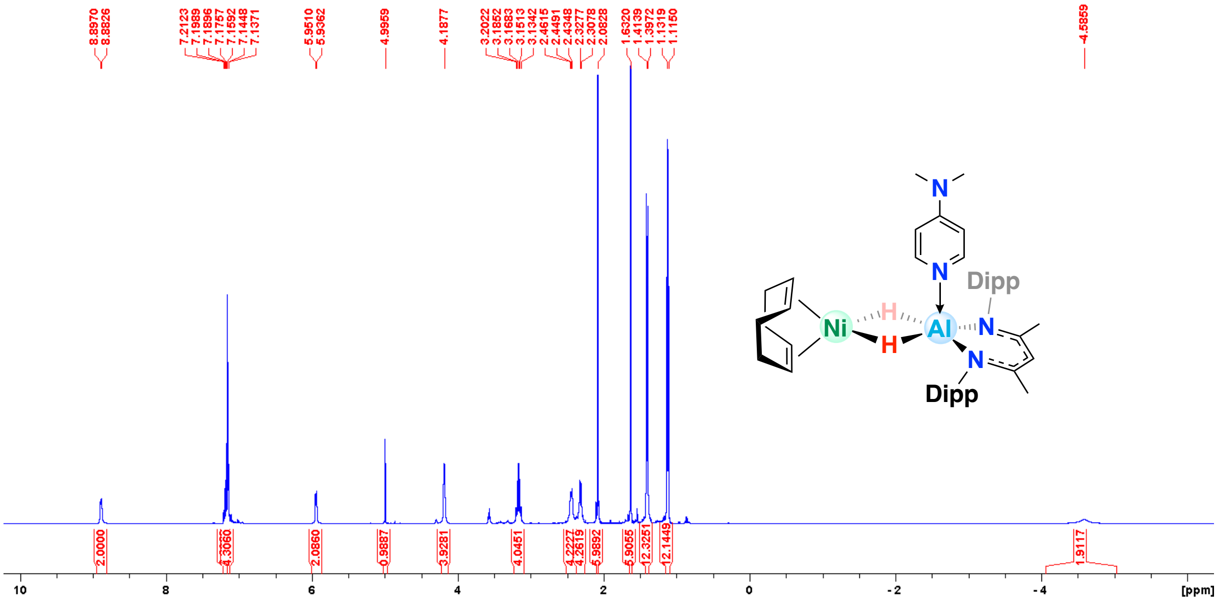

Figure S4 **2·DMAP**, ^{1}H NMR, C_6D_6, 400 MHz, 298 K.

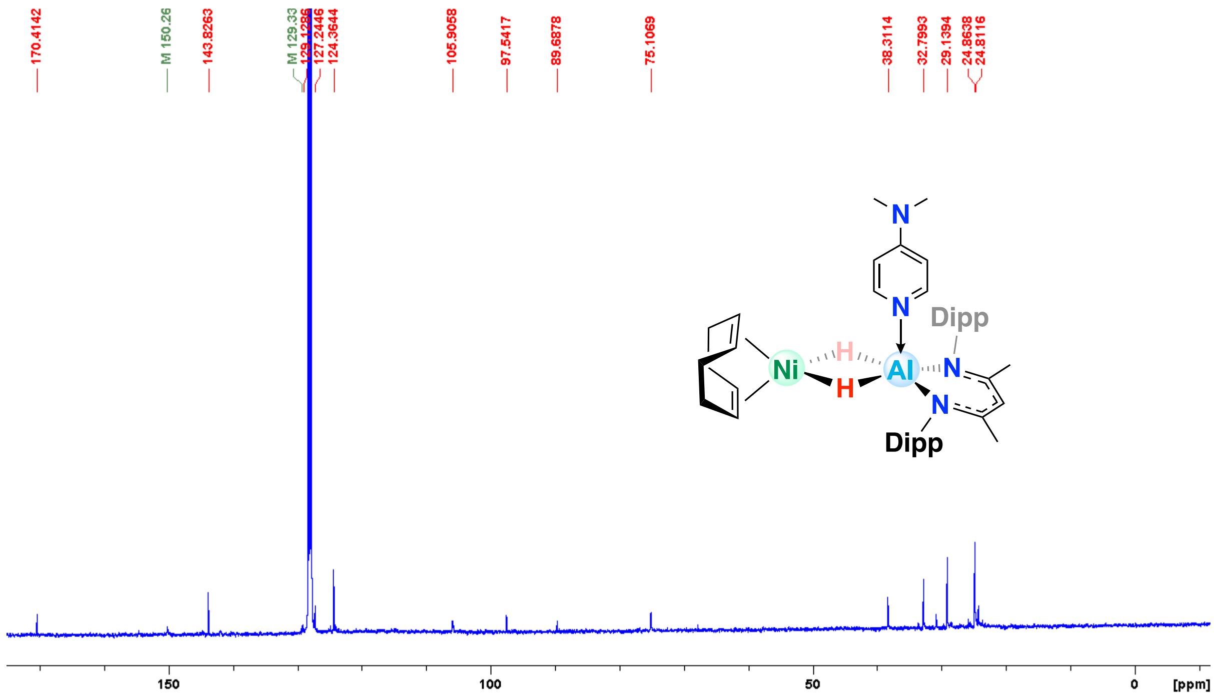

Figure S5 **2·DMAP**, $^{13}C\{^{1}H\}$ NMR, C_6D_6, 100.6 MHz, 298 K.

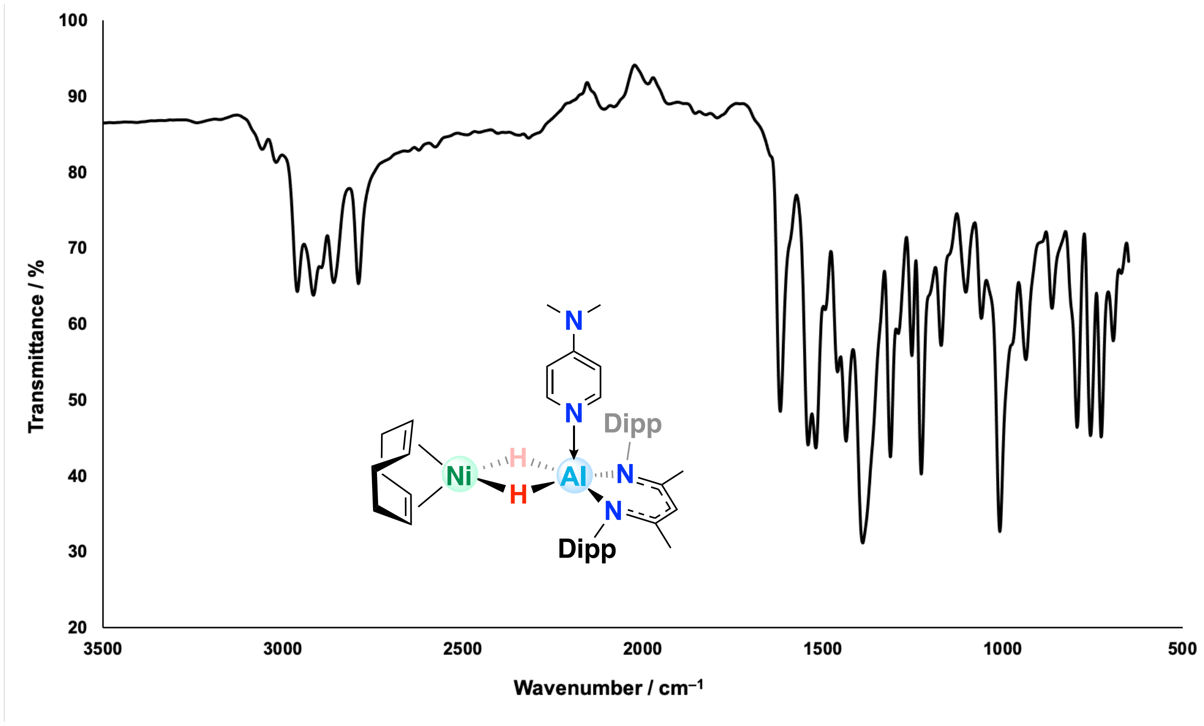

Figure S6 **2·DMAP**, FT-IR (ATR), 298 K.

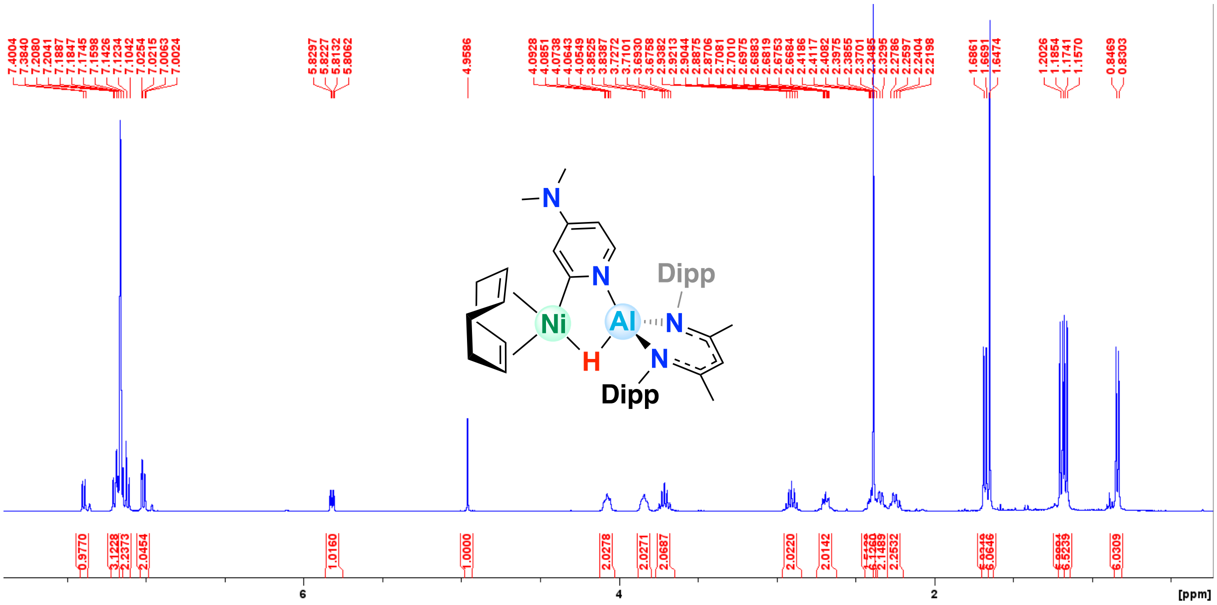

Figure S7 **3**, ^{1}H NMR, C_6D_6, 400 MHz, 298 K.

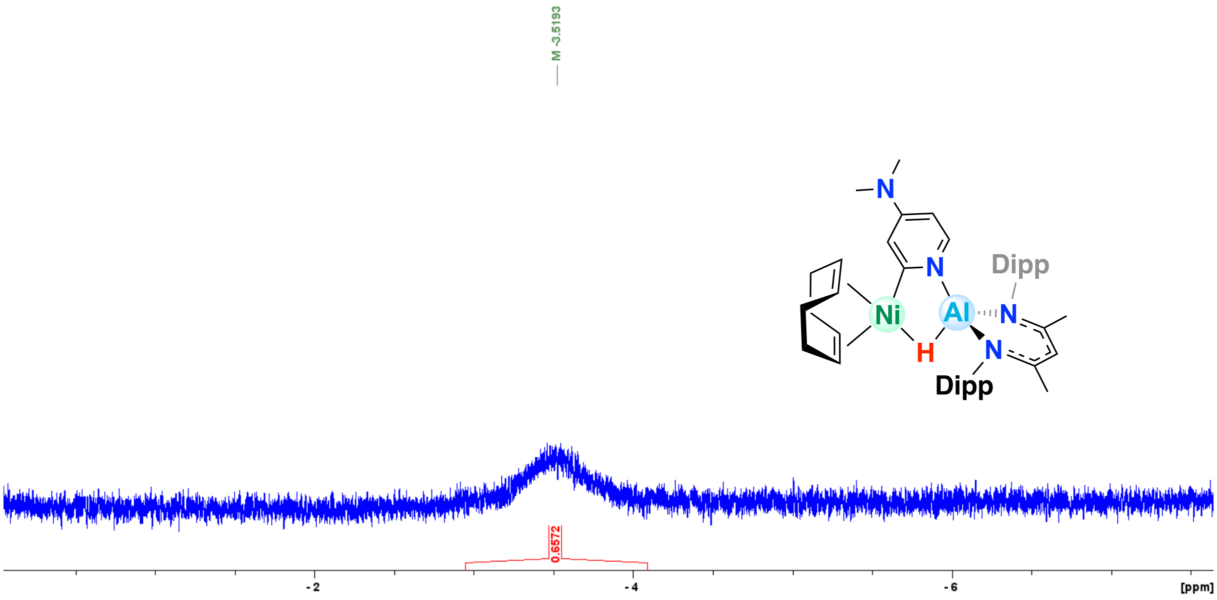

Figure S8 **3**, ^{1}H NMR, C_6D_6, 400 MHz, 298 K. Enhanced view of the hydride signal.

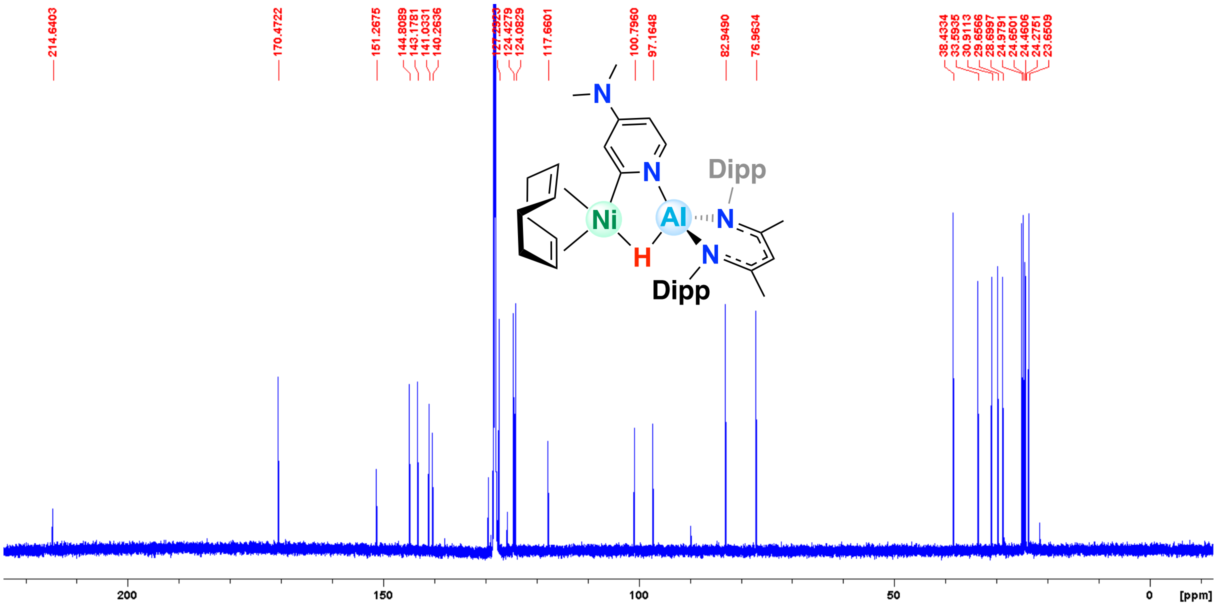

Figure S9 **3**, $^{13}C\{^{1}H\}$ NMR, C_6D_6, 151.0 MHz, 298 K.

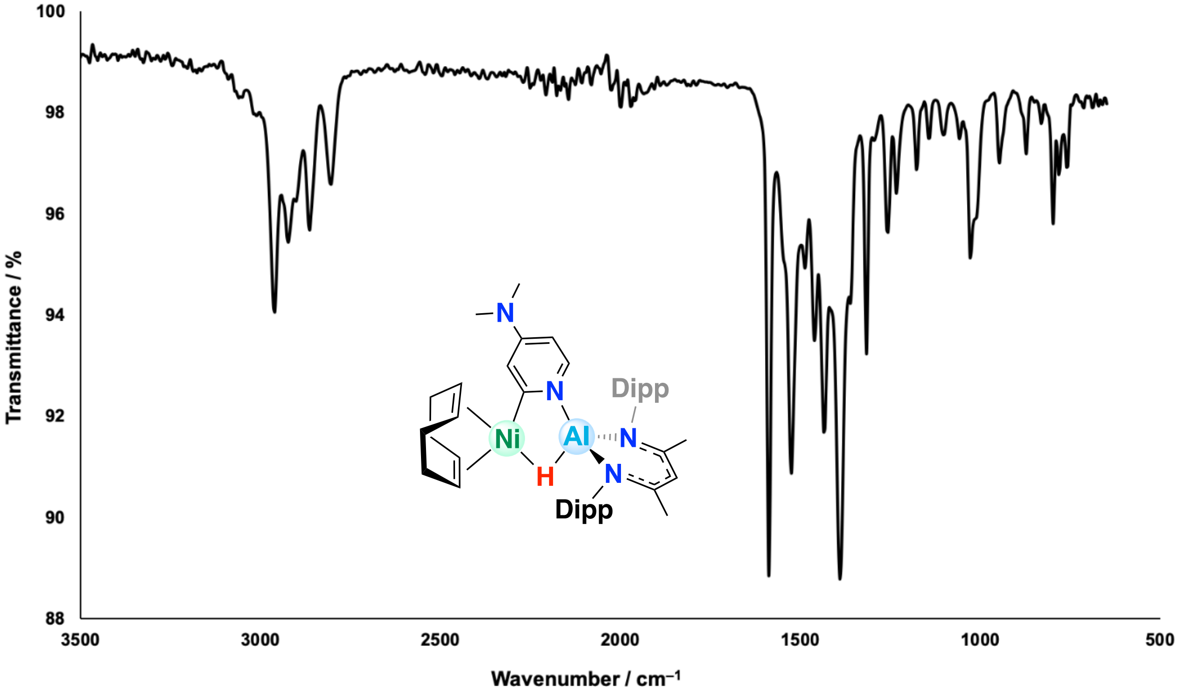

Figure S10 **3**, FT-IR (ATR), 298 K.

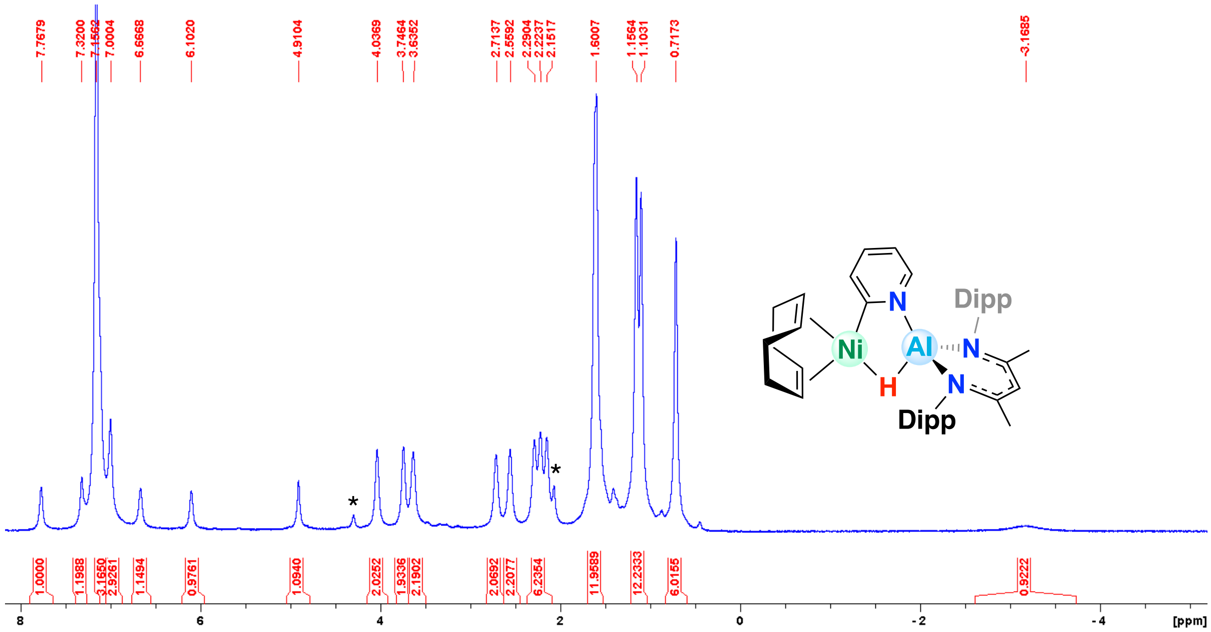

Figure S11 **4**, ^{1}H NMR, C_6D_6, 600 MHz, 298 K. * = [Ni(COD)$_2$]. This ^{1}H NMR spectrum represents the "cleanest" isolated sample of **4** that was obtained (in our hands).

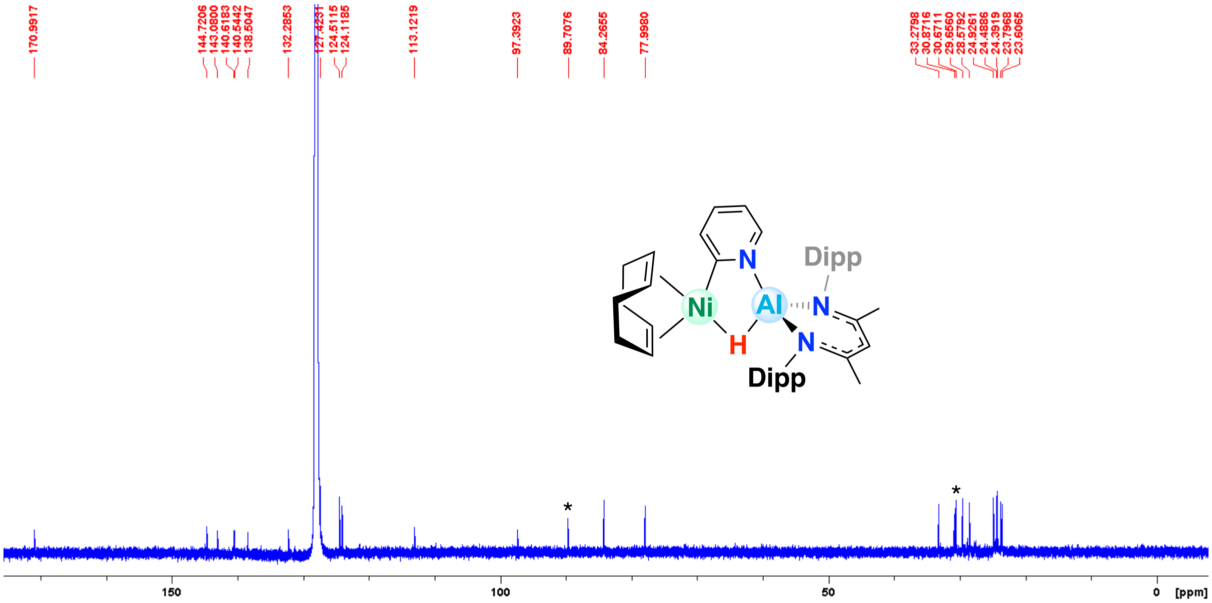

Figure S12 **4**, $^{13}C\{^{1}H\}$ NMR, C_6D_6, 151.0 MHz, 298 K. * = [Ni(COD)$_2$]. This $^{13}C\{^{1}H\}$ NMR spectrum represents the "cleanest" isolated sample of **4** that was obtained (in our hands).

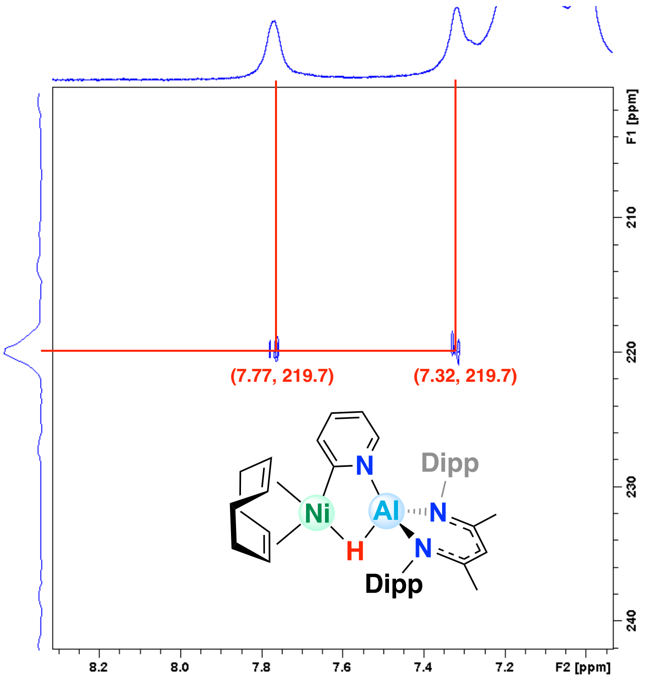

Figure S13 **4**, ^{1}H-^{13}C HMBC (600 MHz, C_6D_6, 298 K) showing the [Ni]-<u>C</u>$_{pyridine}$ signal at δ_C = 219.7 ppm. This ^{1}H-^{13}C HMBC spectrum was obtained on the same sample as the spectra presented in Figures S11 and S12.

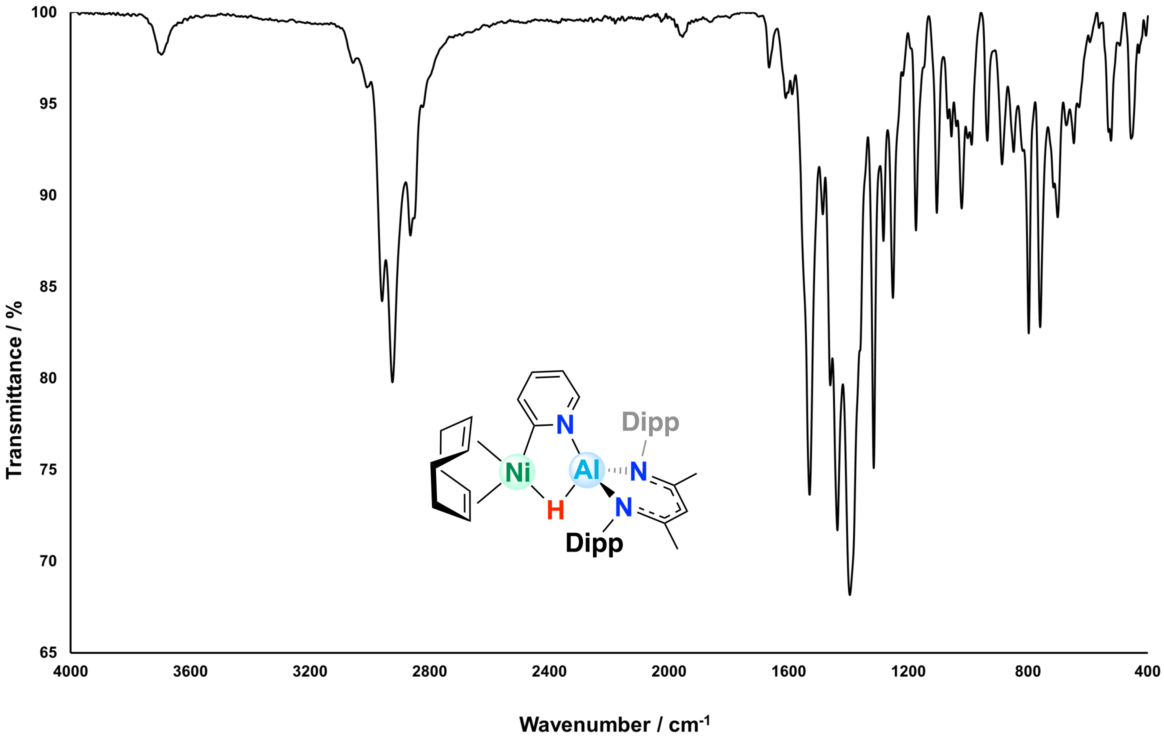

Figure S14 **4**, FT-IR (ATR), 298 K.

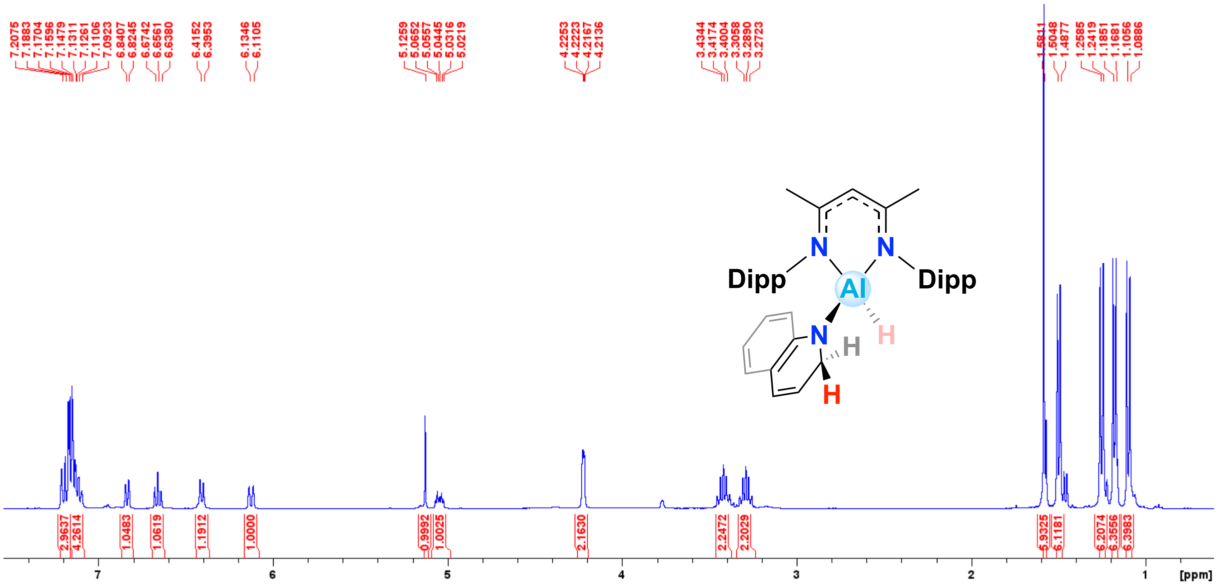

Figure S15 **S1**, ^{1}H NMR, C_6D_6, 400 MHz, 298 K.

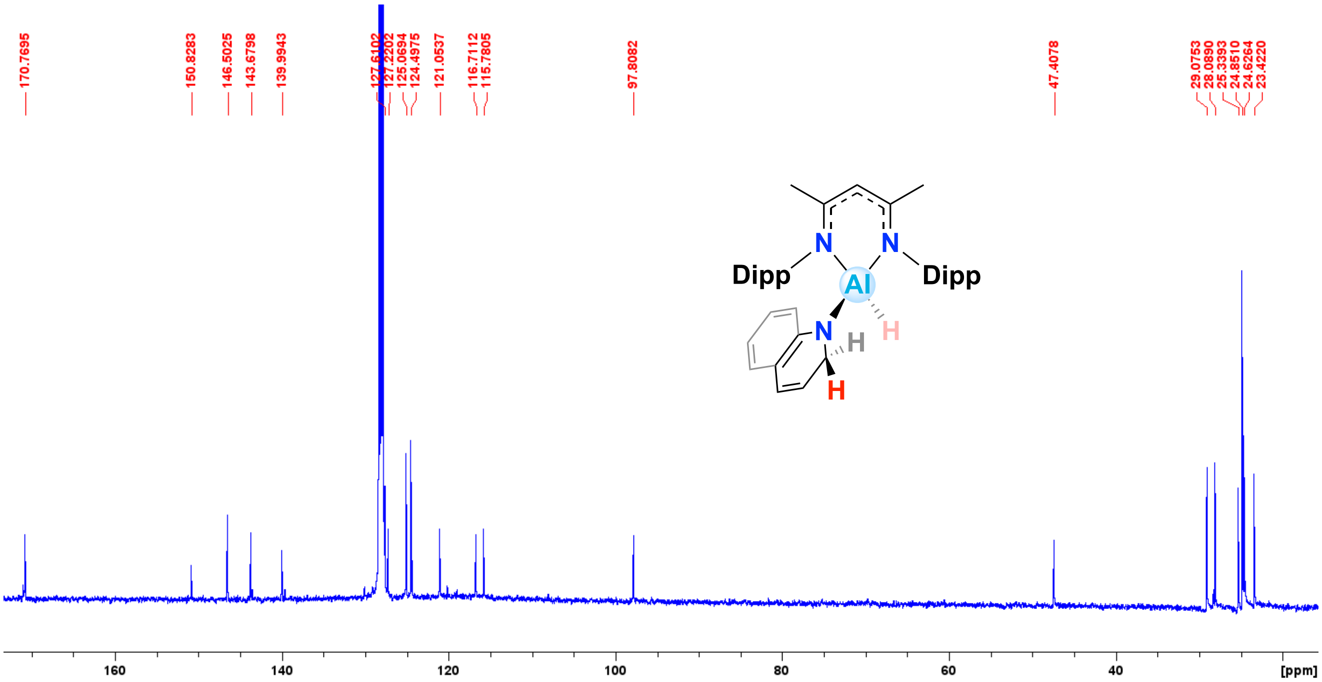

Figure S16 **S1**, $^{13}C\{^{1}H\}$ NMR, C_6D_6, 100.6 MHz, 298 K.

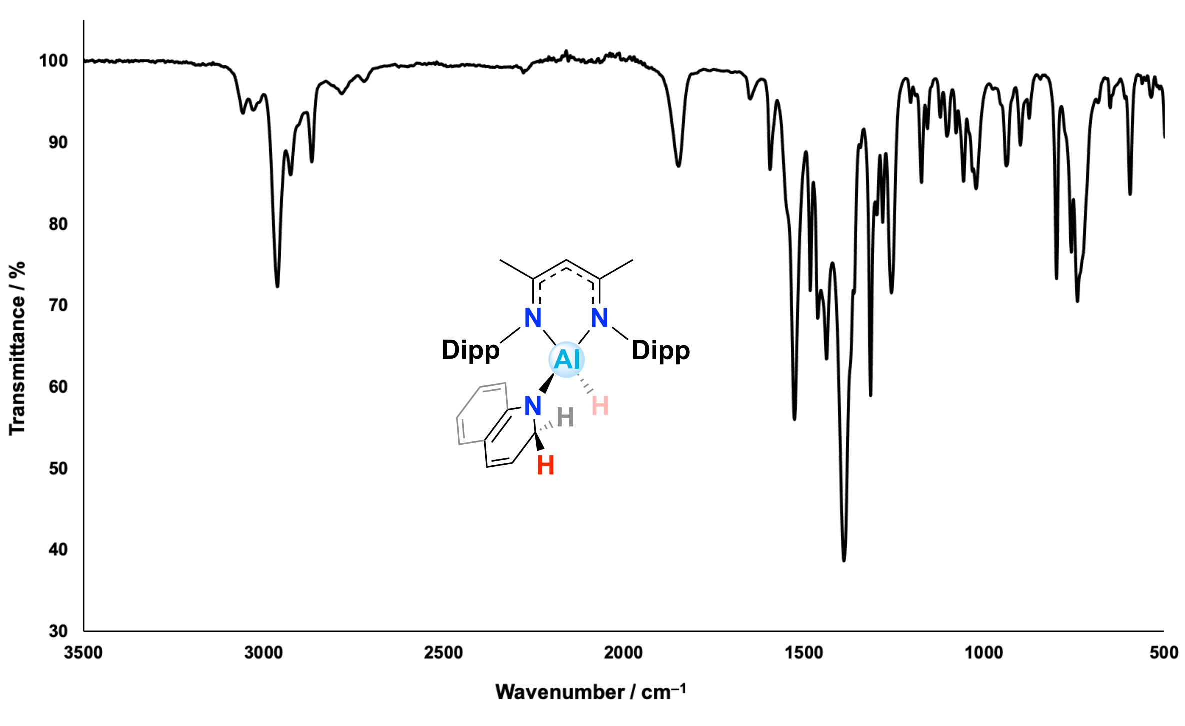

Figure S17 **S1**, FT-IR (ATR), 298 K (ν[Al-H] = 1847 cm^{-1}).

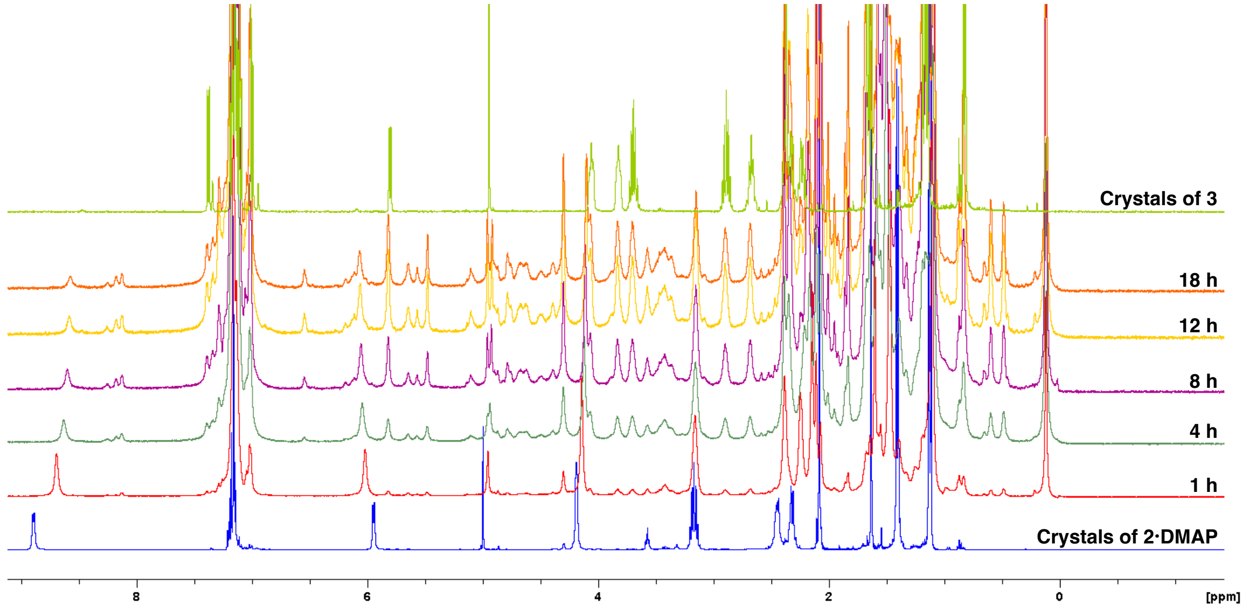

Figure S18 Stacked ^{1}H NMR spectra (400 MHz, C_6D_6, 298 K) for the conversion of **2·DMAP** into **3** over the course of 18 h with 0 mol% of PCy_3.

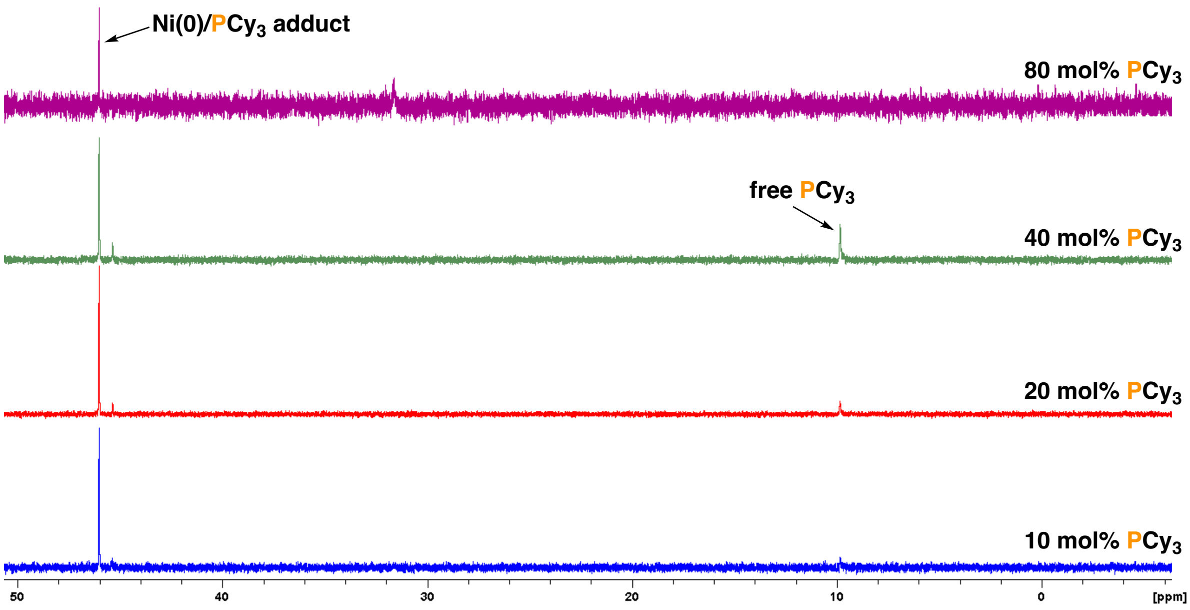

Figure S19 Overlayed $^{31}P\{^{1}H\}$ NMR spectra (242.9 MHz, C_6D_6, 298 K) showing a signal at δ_P = +46 ppm (Ni(0)/PCy_3 adduct)[4] and free PCy_3 at δ_P = +10 ppm. The Ni(0)/PCy_3 refers to ill-defined homometallic complexes.[4–6]

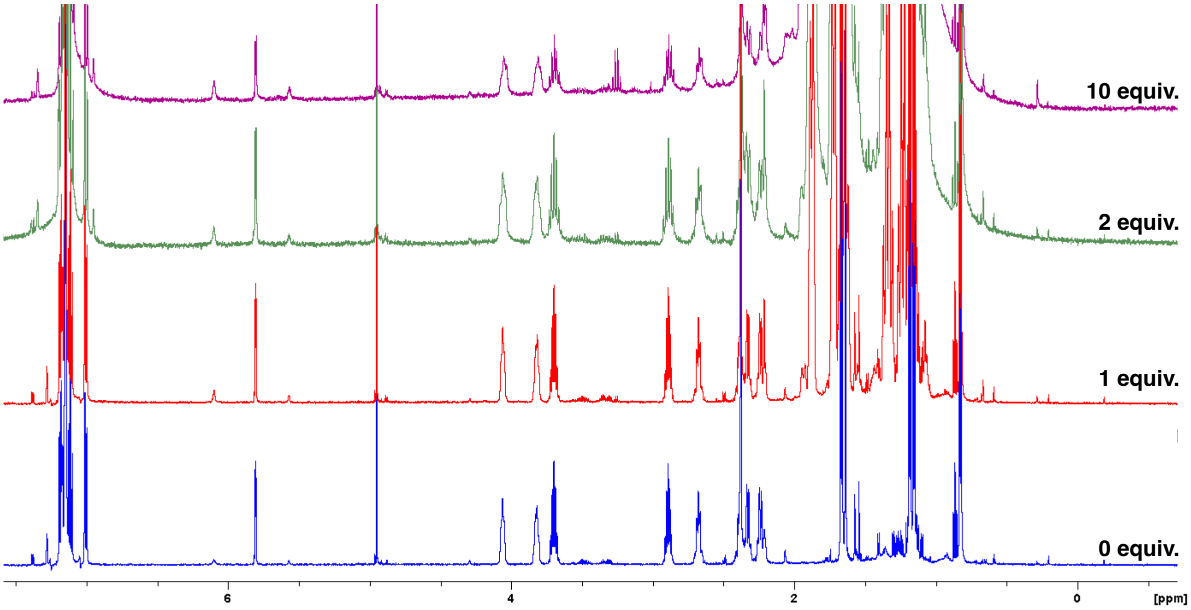

Figure S20 Overlayed ^{1}H NMR spectra (600 MHz, C_6D_6, 298 K) showing a titration of **3** with 0, 1, 2, and 10 equivalents of PCy_3. No reaction was observed.

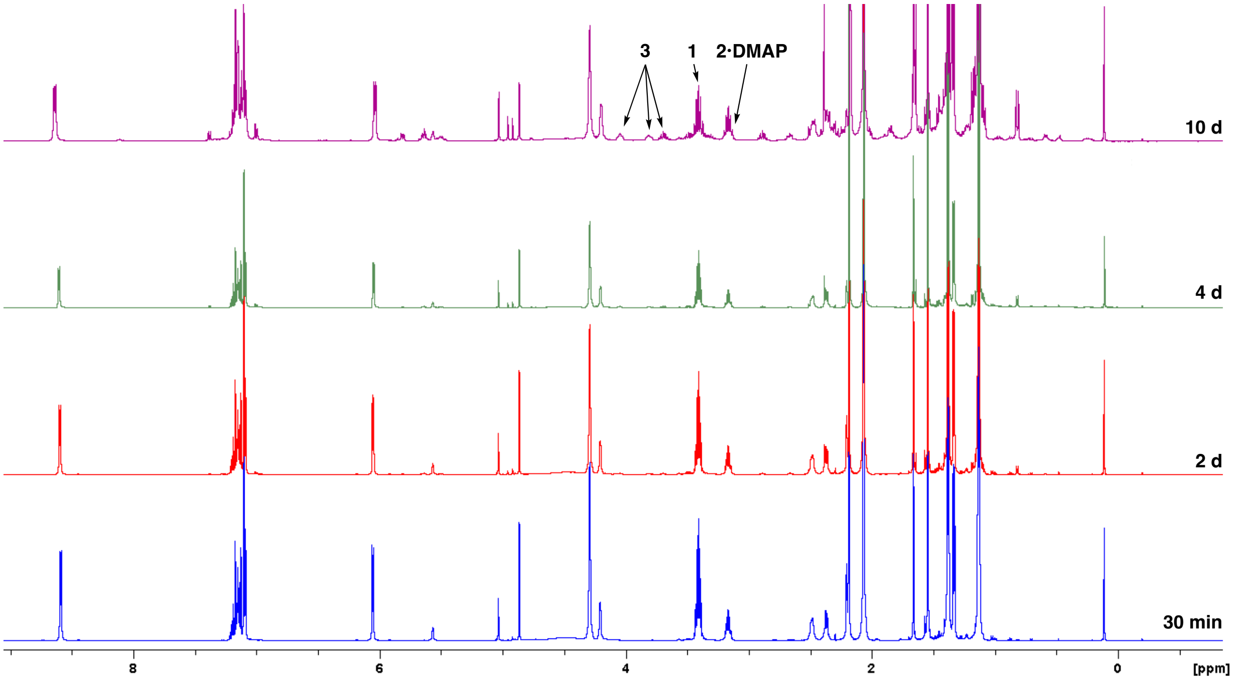

Figure S21 Overlayed ^{1}H NMR (C_6D_6, 600 MHz, 298 K) spectra for the conversion of [Ni(COD)$_2$], DMAP, and **1** into **3** in the absence of PCy_3. The spectroscopic yield of **3** was found to be 20 % after 10 days (with an internal HMDSO standard).

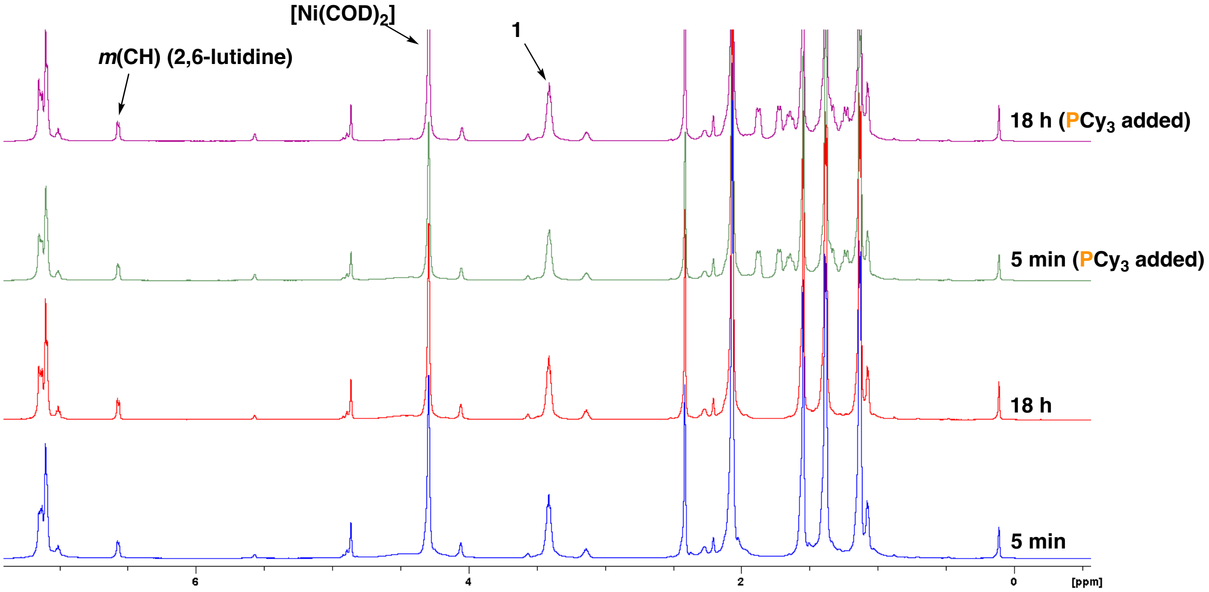

Figure S22 Overlayed ^{1}H NMR (C_6D_6, 600 MHz, 298 K) spectra for the reaction mixture of [Ni(COD)$_2$], **1**, and 2,6-lutidine. No reaction occurred, even in the presence of PCy_3 (20 mol%).

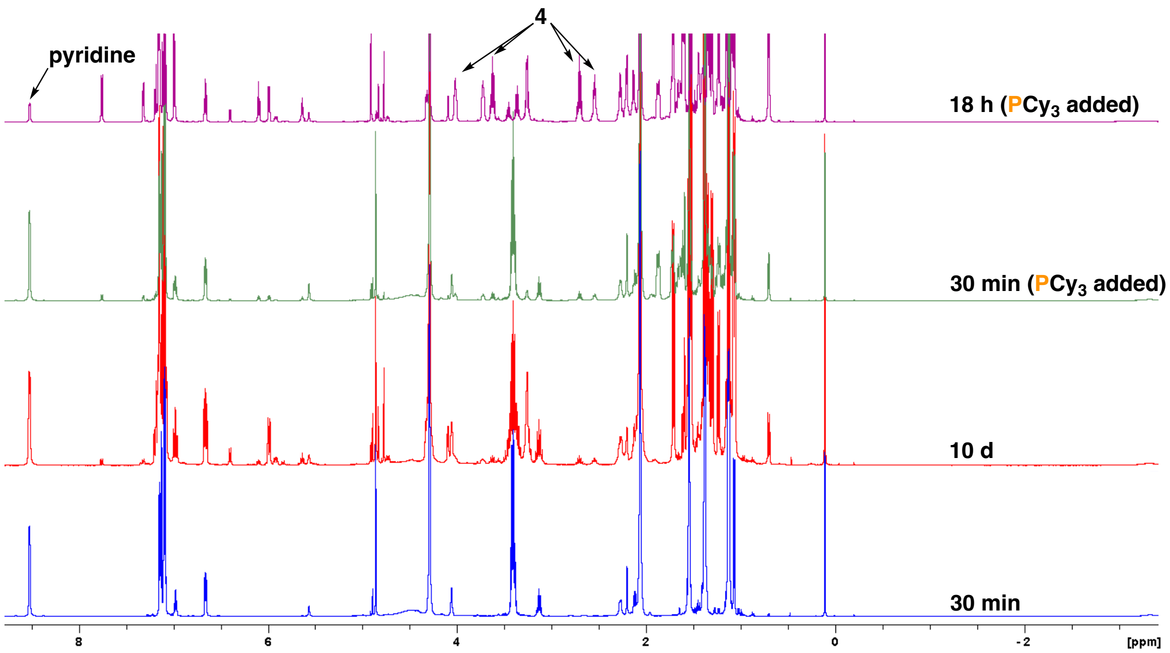

Figure S23 Overlayed ^{1}H NMR (C_6D_6, 600 MHz, 298 K) spectra for the reaction mixture of [Ni(COD)$_2$], **1**, and pyridine. After 10 days, PCy_3 (20 mol%) was added to form **4**.

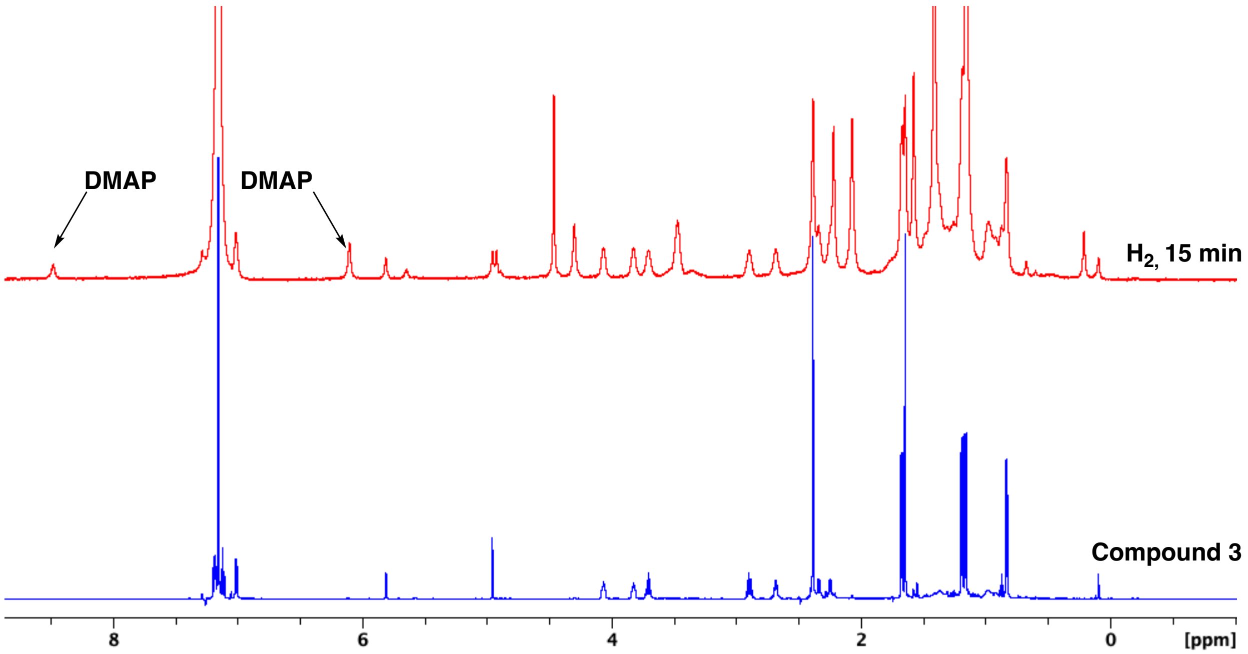

Figure S24 Stacked ^{1}H NMR (C_6D_6, 600 MHz, 298 K) spectra showing a sample of **3** (bottom) exposed to 1 atm. of H_2, releasing DMAP after 15 min.

S4 Equilibrium Analysis of **1** and **2**

In the glovebox, [Ni(COD)$_2$] (10 mg, 0.04 mmol) and **1** (16 mg, 0.04 mmol, 1 equiv.) were combined in 500 μL of toluene-d_8 in a J-Young NMR tube. The mixture was left for 15 min to allow equilibrium to be reached. To obtain concentration values suitable for van't Hoff analysis, an internal standard of HMDSO was added. Starting at 303 K, the sample was analyzed by ^{1}H NMR spectroscopy (400 MHz) every 15 min in increments of 10 K until 193 K.

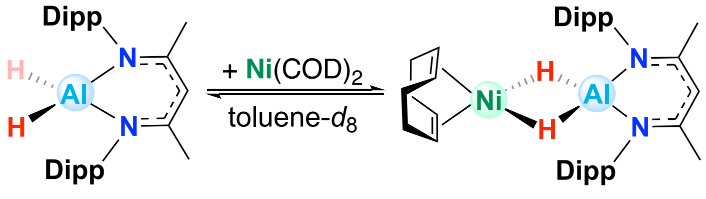

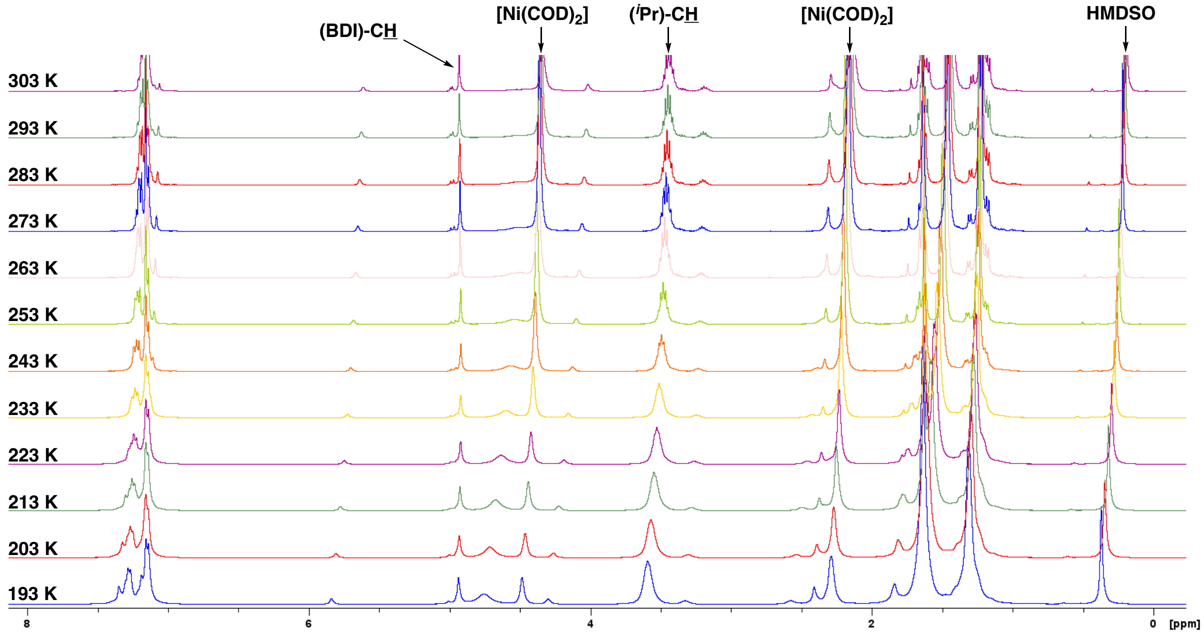

Figure S25 Variable temperature ^{1}H NMR (toluene-d_8, 400 MHz) spectrum for the mixture of **1** with [Ni(COD)$_2$] (with an internal HMDSO standard).

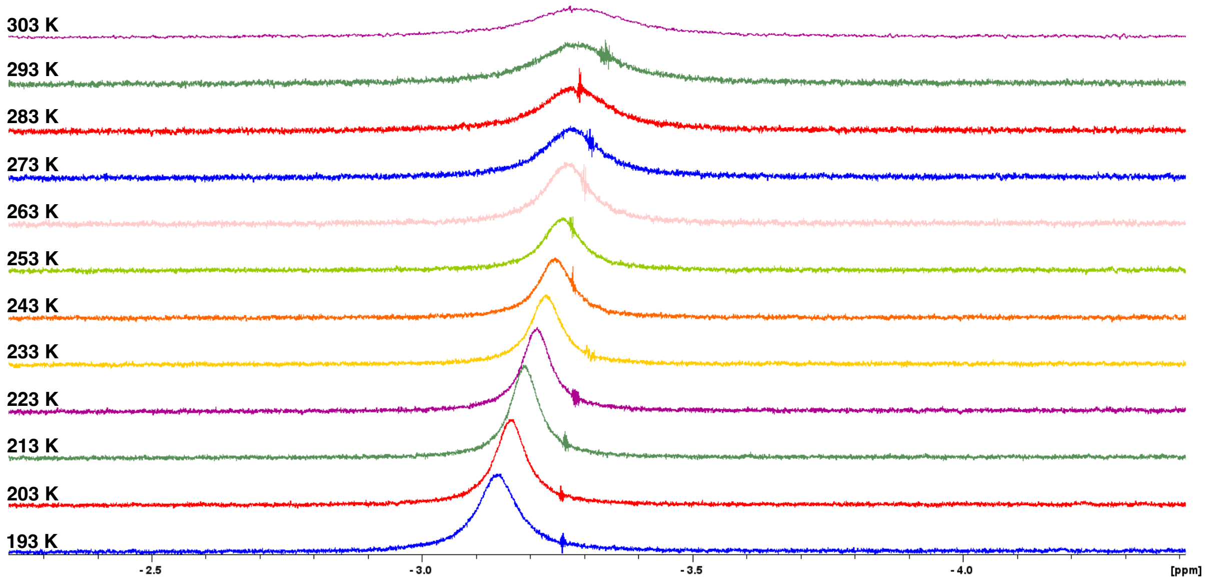

Figure S26 Variable temperature ^{1}H NMR (toluene-d_8, 400 MHz) spectrum for **2** showing the hydride region from –2.5 > δ_H > –4.0 ppm with the broad signal corresponding to the [Ni(μ-H)$_2$Al] protons in **2**.

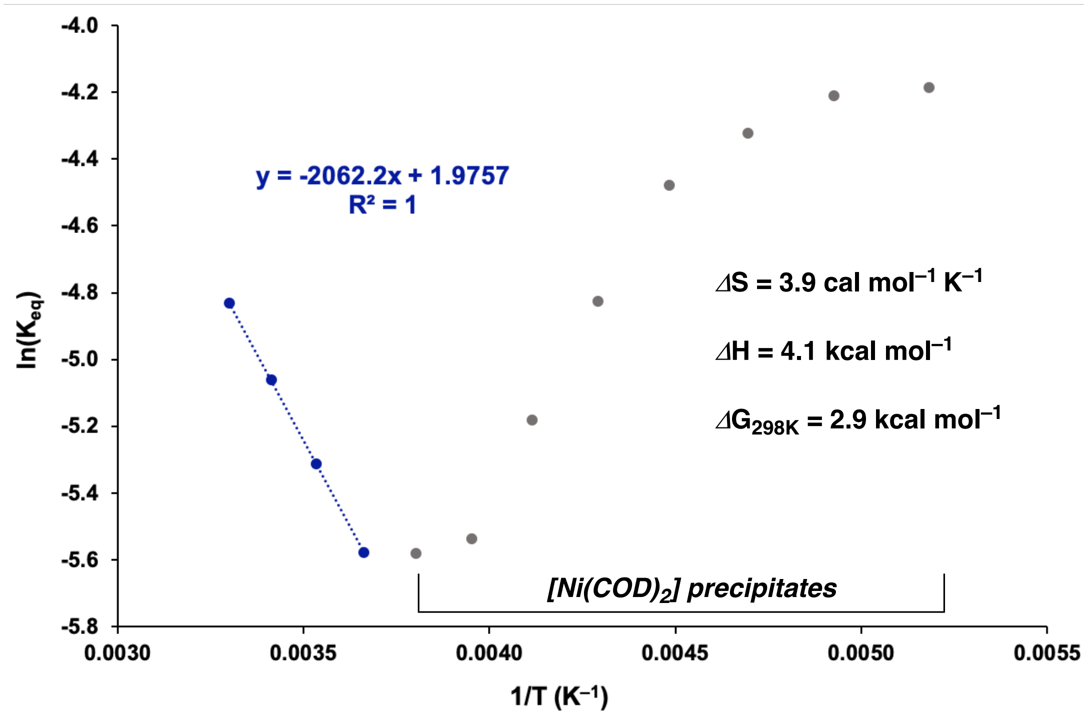

Figure S27 Plot of ln(K_{eq}) versus 1/T to obtain thermochemical values associated with the equilibrium between **1**, [Ni(COD)$_2$], and **2**. Blue markers represent spectra where equilibrium was observed. Grey markers represent spectra where the [Ni(COD)$_2$] concentration decreased as a result of precipitation from toluene-d_8 at lower temperatures.

In the glovebox, 10 mg of **2·DMAP** was dissolved in 700 μL of C_6D_6. A ^{1}H NMR spectrum was acquired. The NMR tube was cycled back into the glovebox, and 10 equivalents of 1,5-cyclooctadiene (COD) was added. The solution immediately changed colour from red to light yellow. A ^{1}H NMR spectrum was acquired after 5 min. The two ^{1}H NMR spectra are shown below in Figure S28 S.

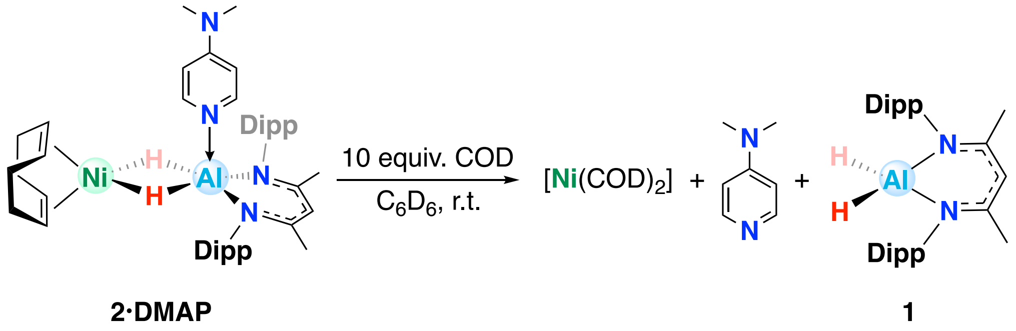

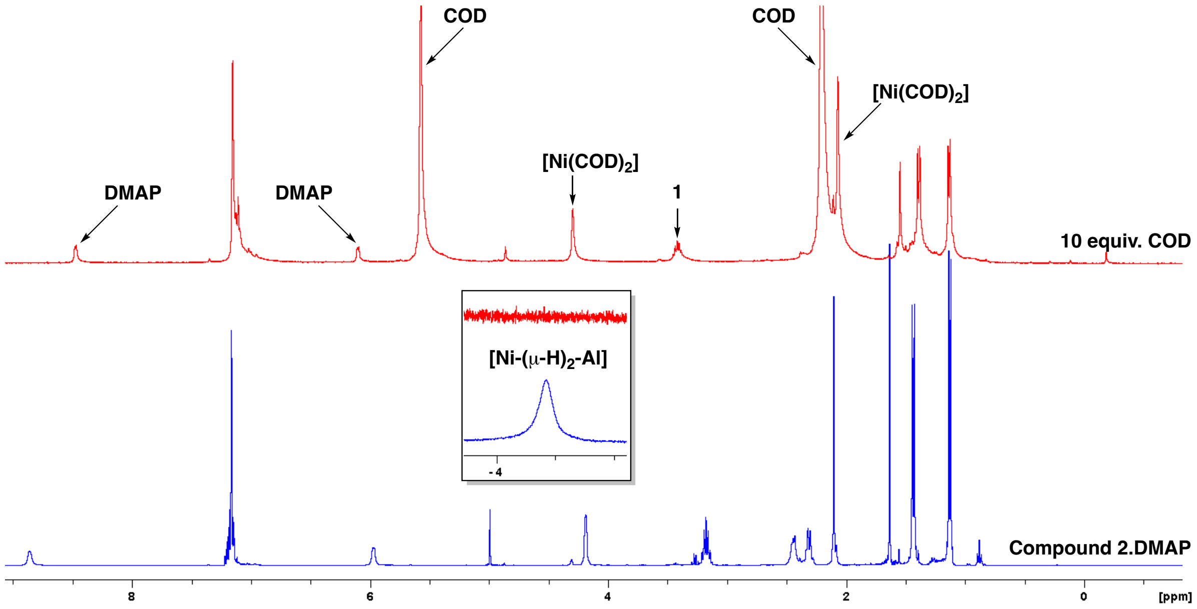

Figure S28 Stacked ^{1}H NMR (C_6D_6, 400 MHz, 298 K) spectra showing a sample of **2·DMAP** (bottom) followed by the addition of 10 equivalents of 1,5-cyclooctadiene (COD) (top), forming [Ni(COD)$_2$], DMAP and **1**. Insert shows the disappearance of the [Ni(μ-H)$_2$Al] signal upon addition of the COD.

S5 Kinetic Data for the C(sp^2)–H Activation

General procedure for a typical experiment to monitor the transformation of **2·DMAP** to **3**:

In the glovebox, 500 μL of a C_6D_6 stock solution of **2·DMAP** (0.007 mmol) was added to a J. Young NMR tube. Next, 5.2 μL of an HMDSO stock solution was added as an internal standard. The appropriate amount of PCy_3 was added as a stock solution in C_6D_6. In all cases, the final sample volume was made up to be 700 μL. It took 5 min to remove the sample from the glovebox, bring it to the NMR spectrometer, and setup the ^{1}H NMR acquisition parameters (lock, tune, shim etc). These 5 min of preparation were accounted for in the analysis of the kinetic data. All reactions were conducted at room temperature, and ^{1}H NMR spectra acquired at 298 K.

For 0, 10, 20, 40 and 80 mol% of PCy_3, ^{1}H NMR spectra were acquired every 30, 15, 15, 10 and 5 min, respectively. In all cases data was plotted as ln([**2·DMAP**]/[**2·DMAP**]$_0$) versus time (in minutes) to determine the value of k_{obs} (Figures S28-S32). For all kinetic runs, data was plotted up until **2·DMAP** was consumed by 60 %.

The disappearance of the signal corresponding to the (^{i}Pr)-C<u>H</u> proton (δ_H = 3.17 ppm) in **2** was monitored against the internal HMDSO standard.

Stock solutions:

2·DMAP: 30 mg (0.042 mmol) was dissolved in 3 mL of C_6D_6 (0.014 M). This solution was stored in the freezer at –35 °C until needed.

HMDSO: 100 μL (0.472 mmol) was dissolved in 2 mL of C_6D_6 (0.236 M). This solution was stored at room temperature until needed.

PCy_3: 103 mg (0.368 mmol) was dissolved in 1.5 mL of C_6D_6 (0.245 M). This solution was stored at room temperature until needed.

PCy_3 (mol%)	k_{obs} (min^{-1})
0	1.3 x 10^{-3}
10	1.7 x 10^{-3}
20	6.2 x 10^{-3}
40	1.3 x 10^{-2}
80	2.3 x 10^{-2}

Table S1 Rate constants (k_{obs}) obtained with different concentrations of PCy_3. k_{obs} increased proportionally with the concentration of PCy_3, indicating the transformation of **2·DMAP** into **3** was first order with respect to PCy_3.

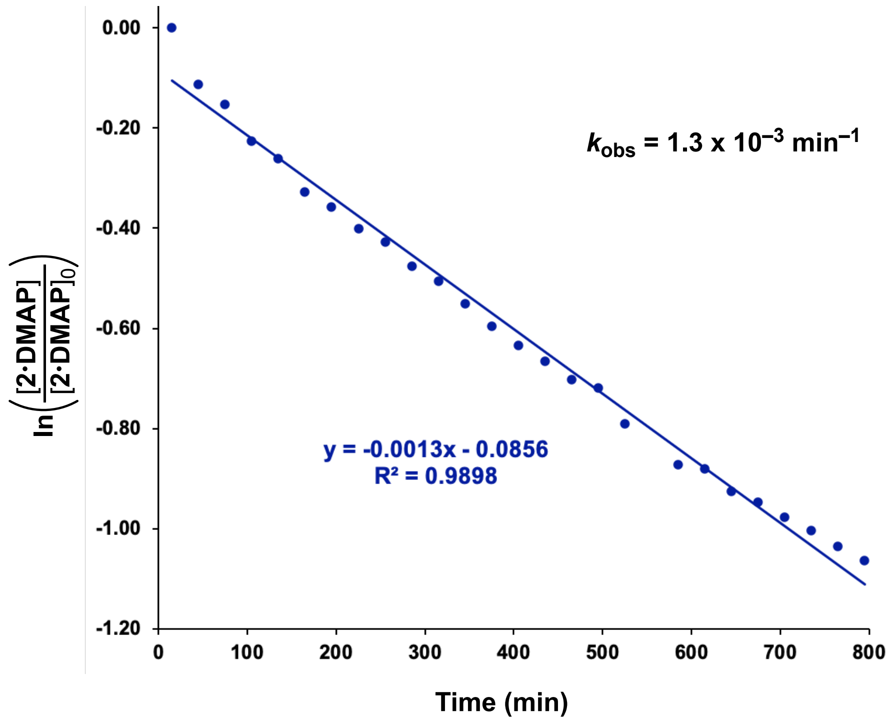

Figure S29 Plot used to obtain k_{obs} for the transformation of **2·DMAP** into **3** using 0 mol% PCy_3.

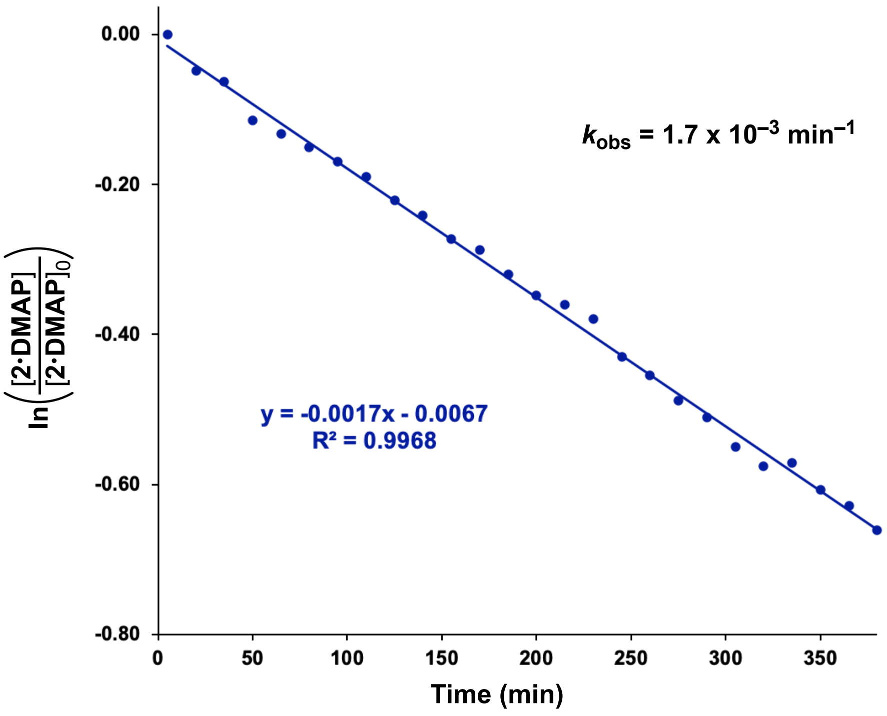

Figure S30 Plot used to obtain k_{obs} for the transformation of **2·DMAP** into **3** using 10 mol% PCy_3.

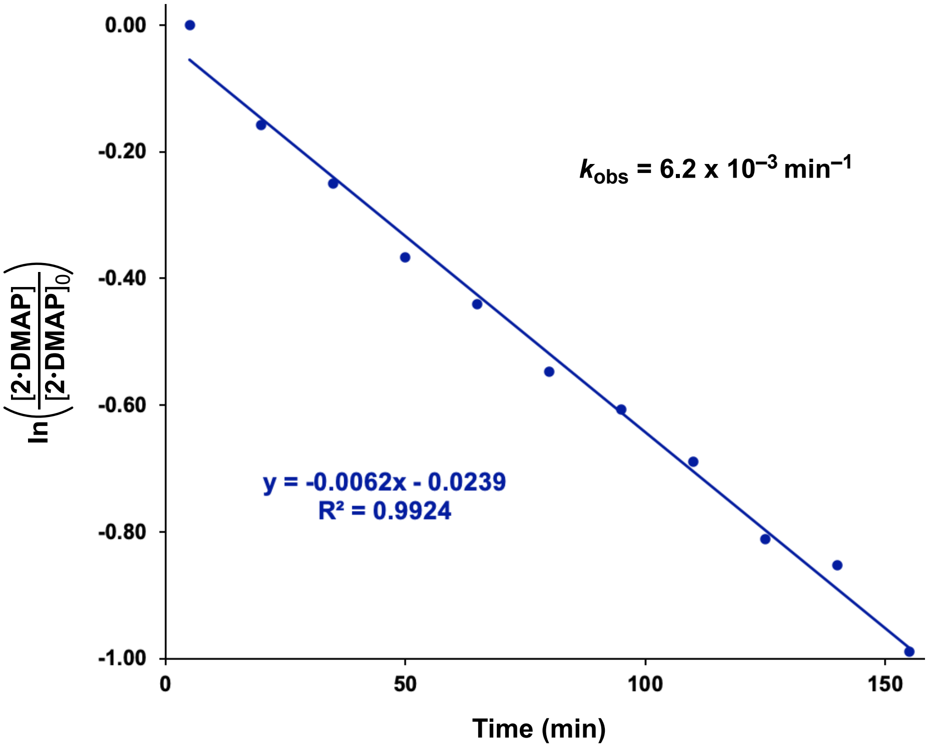

Figure S31 Plot used to obtain k_{obs} for the transformation of **2·DMAP** into **3** using 20 mol% PCy_3.

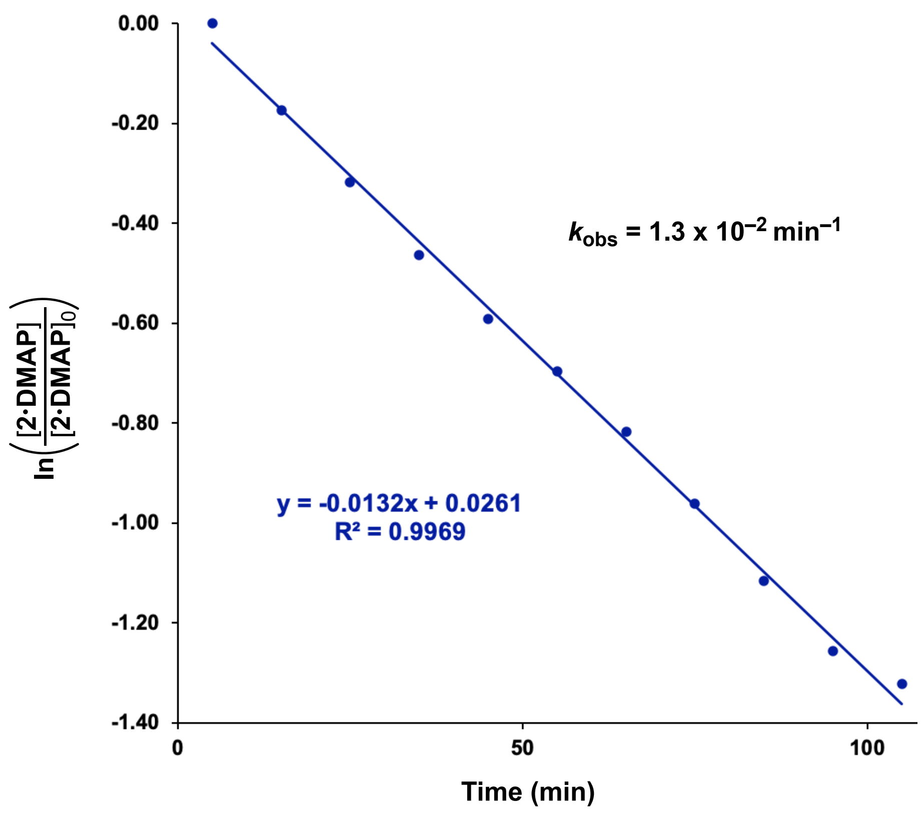

Figure S32 Plot used to obtain k_{obs} for the transformation of **2·DMAP** into **3** using 40 mol% PCy_3.

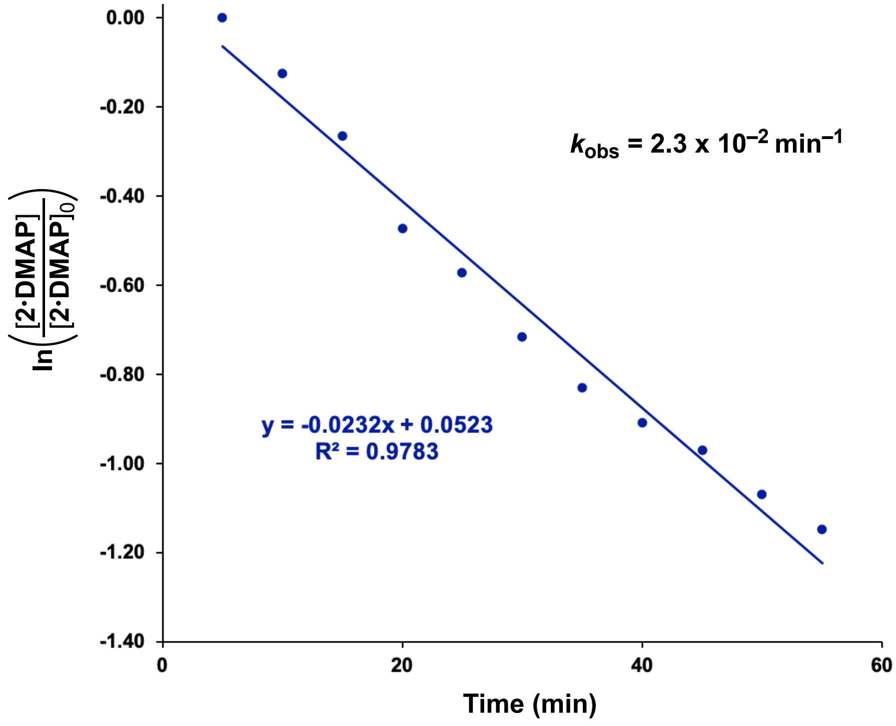

Figure S33 Plot used to obtain k_{obs} for the transformation of **2·DMAP** into **3** using 80 mol% PCy_3.

General procedure for the experiments to determine the kinetic isotope effect (KIE):

In the glovebox, 10 mg of [Ni(COD)$_2$] and 17 mg of **1** were combined in 500 μL of C_6D_6 in an NMR tube. To this solution was added 1 equivalent of pyridine or pyridine-d$_5$. Next, 5.2 μL of an HMDSO stock solution was added as an internal standard. The appropriate amount of PCy_3 (20 mol%) was added as a stock solution in C_6D_6. In each case, the final sample volume was made up to be 700 μL. It took 5 min to remove the sample from the glovebox, bring it to the NMR spectrometer, and setup the ^{1}H NMR acquisition parameters (lock, tune, shim etc). These 5 min of preparation were accounted for in the analysis of the kinetic data (the first data point was acquired 10 min after starting the reaction in the glovebox). All reactions were conducted at room temperature, and ^{1}H NMR spectra acquired at 298 K.

For each experiment, ^{1}H NMR spectra were acquired every 10 min. In both cases, the first 6 data points were plotted as ln([**4**]) or ln([**4-d$_4$**]) versus time (min) to create a linear plot, where the slope was k_{obs} (Figures S34-S35). The appearance of the signal corresponding to the (^{i}Pr)-C$\underline{H}$ proton (δ_H = 2.71 ppm) in **4** or **4-d$_4$** was monitored against the internal HMDSO standard.

Stock solutions:

HMDSO: 100 μL (0.472 mmol) was dissolved in 2 mL of C_6D_6 (0.236 M). This solution was stored at room temperature until needed.

PCy_3: 103 mg (0.368 mmol) was dissolved in 1.5 mL of C_6D_6 (0.245 M). This solution was stored at room temperature until needed.

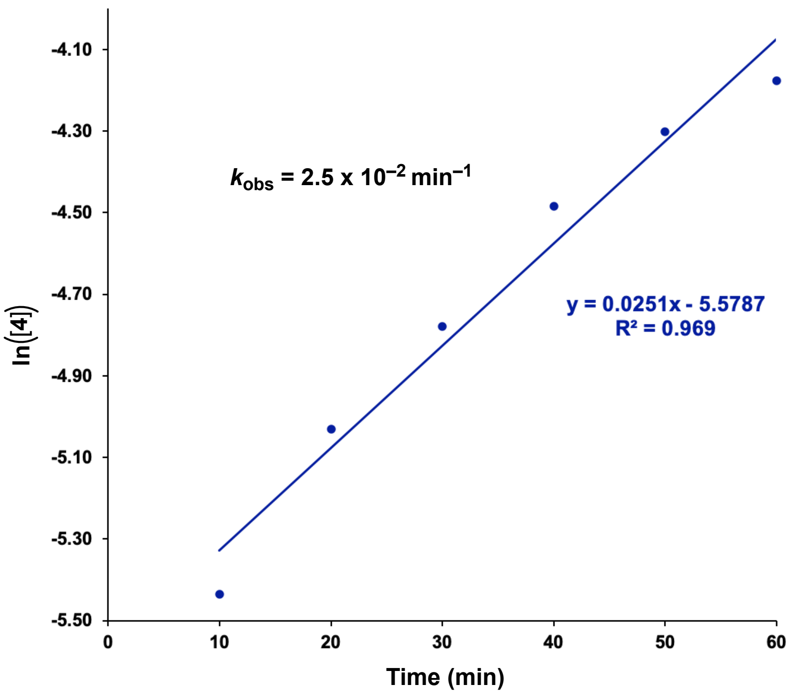

Figure S34 Plot used to obtain k_{obs} for the formation of **4** using 20 mol% PCy_3.

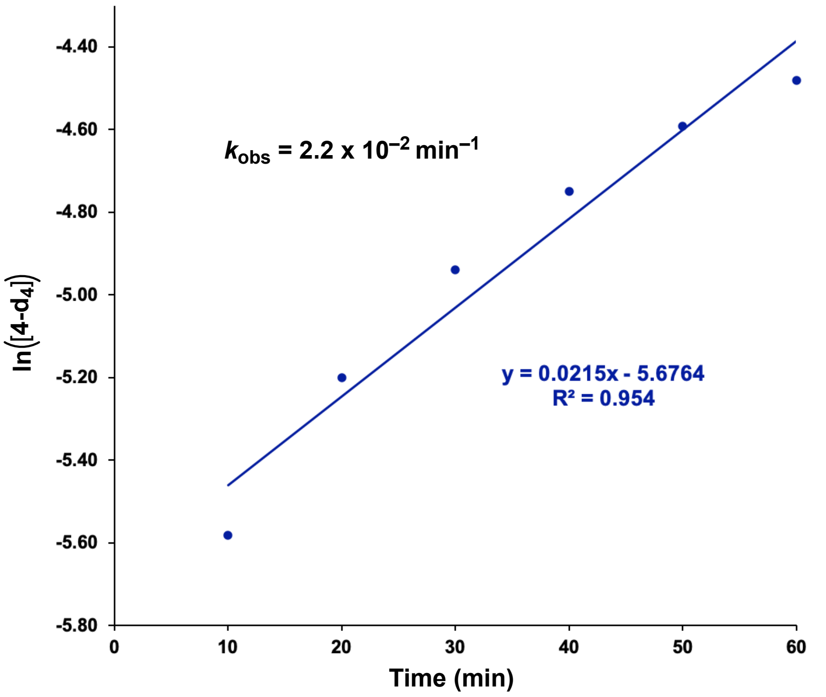

Figure S35 Plot used to obtain k_{obs} for the formation of **4-d**$_4$ using 20 mol% PCy_3.

General procedure for the competition experiment to determine the kinetic isotope effect (KIE):
In the glovebox, [Ni(COD)$_2$] (10 mg), and **1** (17 mg) were each dissolved in 0.75 mL of C_6H_6 and combined in a 20 mL scintillation vial. Under vigorous stirring, pyridine (29 μL, 10 equiv.) and pyridine-d$_5$ (29 μL, 10 equiv.) were added to the reaction vessel. The 4 reactants were stirred for an additional 5 min to ensure homogeneous mixing. After 5 min, and under vigorous stirring, 20 mol% PCy$_3$ was added as a stock solution in C_6H_6. The colour gradually changed from orange to dark red over the course of 1 h. The reaction was stirred for a total of 18 h in the glovebox at room temperature. Next, the solvent and excess pyridine/pyridine-d$_5$ were removed *in-vacuo*. The solids were dissolved in 3 mL of *n*-pentane and filtered through a 1 cm^3 plug of Celite®. The *n*-pentane was removed *in-vacuo* and the solids dissolved in C_6D_6. A ^{1}H NMR spectrum was acquired (see Figure S35 below).

A KIE was calculated by integrating the (pyridine)-C$\underline{H}_{aromatic}$ proton at δ_H = 7.77 ppm and setting the value to 1H, then integrating the (^{i}Pr)-C$\underline{H}$ protons at δ_H = 2.71 ppm. If the competition experiment provided **4** and **4-d$_1$** in a 50/50 mixture, then the (^{i}Pr)-C$\underline{H}$ protons would integrate to 4H (2H from **4** and 2H from **4-d$_1$**). As shown in Figure S35, the integral values were 1.000:4.2415 for (pyridine)-C$\underline{H}_{aromatic}$ and (^{i}Pr)-C$\underline{H}$, respectively. This ratio corresponds to a KIE of 0.9. Given the error associated with manually integrating signals for a single ^{1}H NMR spectrum, we are confident within an error of ± 0.2. This KIE value of 0.9 ± 0.2 determined from the competition experiment falls within range of the KIE value determined from the side-by-side kinetic runs.

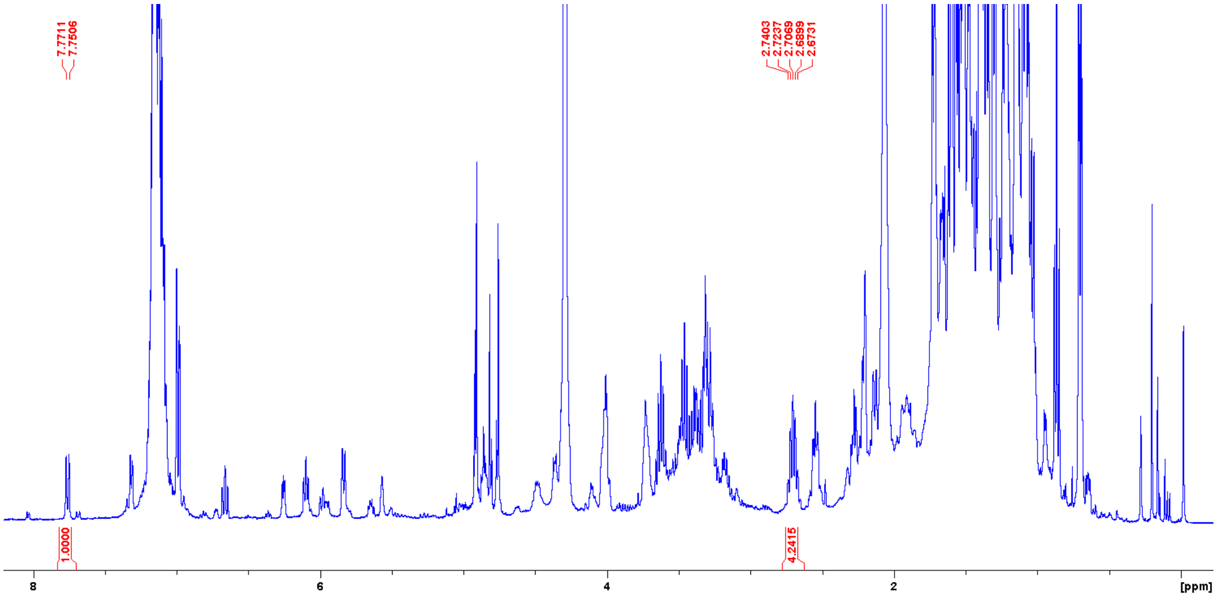

Figure S36 ^{1}H NMR spectrum (C_6D_6, 400 MHz, 298 K) of the reaction mixture from a competition experiment between pyridine and pyridine-d_5. Integrated signals correspond to the (pyridine)-C$\underline{H}_{aromatic}$ proton at δ_H = 7.77 (set to 1H; from **4**) and the (^{i}Pr)-C$\underline{H}$ protons at δ_H = 2.71 ppm (from both **4** and **4-d$_1$**). The integral values correspond to a KIE of 0.9 ± 0.2.

S6 X-Ray Crystallography

S6.1 Crystal data

data	2	2·DMAP	3
CCDC number	2417828	2417829	2417830
formula	$C_{37}H_{55}AlN_2Ni$, 1(C_5H_{12})	$C_{44}H_{65}AlN_4Ni$, 1(C_7H_8), 0.5(C_5H_{12})	$C_{44}H_{63}AlN_4Ni$
formula weight (g·mol^{-1})	685.66	863.89	733.67
colour, habit	red/orange dichroic block	dark red block	orange blocky needle
crystal size (mm)	0.309 x 0.289 x 0.217	0.262 x 0.164 x 0.117	0.273 x 0.133 x 0.131
crystal system	orthorhombic	monoclinic	orthorhombic
space group	$P2_12_12$ (no. 18)	$P2_1/n$ (no. 14)	$Pbca$ (no. 61)
a (Å)	10.7009(3)	14.7447(3)	18.4811(6)
b (Å)	16.2713(3)	18.0724(4)	19.2769(6)
c (Å)	11.9292(2)	20.3216(5)	23.0406(6)
α (°)	90.0	90	90
β (°)	90.0	109.623(2)	90
γ (°)	90.0	90	90
V (Å^3)	2077.09(8)	5100.6(2)	8208.5(4)
Z	2[b]	4	8
T (K)	172.9(3)	173(4)	173.00(14)
D_c (g·cm^{-3})	1.096	1.125	1.187
radiation used, μ (mm^{-1})	Cu-Kα, 1.075	Cu-Kα, 0.983	Mo-Kα, 0.529
F(000)	748	1876	3168
absorption correction	analytical	analytical,	analytical
min-max transmission	0.773 - 0.838	0.851 - 0.919	0.899 - 0.950
Θ range for data collection (°)	3.705 - 73.540	3.363 - 73.935	2.444 - 28.202
no. of unique reflns measured, obs [F > 4σ(F)]	4047, 3443	17660, 12557	8516, 5853
R_{int}, R_{sigma}	0.0423, 0.0645	0.0477, 0.0376	0.0349, 0.0511
completeness to Θ(full) (°)	0.997 to 67.684	0.999 to 67.684	0.992 to 25.242
no. of parameters, restraints	244, 24	605, 174	467, 0
R_1, wR_2 [F > 4σ(F)] [a]	0.0388, 0.0881	0.0591, 0.1611	0.0414, 0.0980
R_1, wR_2 [all data] [a]	0.0487, 0.0939	0.0812, 0.1746	0.0735, 0.1136
GooF	1.009	1.041	1.015
largest diff. Fourier peak, hole (eÅ^{-3})	0.413, -0.224	0.577, -0.331	0.321, -0.332

data	4	S1
CCDC number	2417831	2417832
formula	$C_{42}H_{58}AlN_3Ni$	$C_{38}H_{50}AlN_3$
formula weight (g·mol^{-1})	690.60	575.79
colour, habit	clear orange prism	yellow column
crystal size (mm)	0.234 x 0.212 x 0.089	0.288 x 0.238 x 0.189
crystal system	monoclinic	monoclinic
space group	$P2_1$/n (no. 14)	$P2_1$/c (no. 14)
a (Å)	11.9136(6)	11.9165(3)
b (Å)	20.0514(10)	22.9236(6)
c (Å)	16.1104(8)	12.8983(3)
α (°)	90	90
β (°)	94.602(2)	98.089(2)
γ (°)	90	90
V (Å^3)	3836.1(3)	3488.36(15)
Z	4	4
T (K)	110.00	173.00(10)
D_c (g·cm^{-3})	1.196	1.096
radiation used, μ (mm^{-1})	Mo-Kα, 0.561	Cu-Kα, 0.710
F(000)	1488	1248
absorption correction	multi-scan	analytical
min-max transmission	0.6723 - 0.7458	0.870 - 0.913
Θ range for data collection (°)	2.733 - 29.183	3.747 - 73.766
no. of unique reflns measured, obs [F > 4σ(F)]	10334, 7450	6748, 4503
R_{int}, R_{sigma}	0.1061, 0.0437	0.0485, 0.0745
completeness to Θ(full) (°)	0.999 to 25.242	0.989 to 67.684
no. of parameters, restraints	439, 0	394, 0
R_1, wR_2 [F > 4σ(F)] [a]	0.0393, 0.0801	0.0521, 0.1217
R_1, wR_2 [all data] [a]	0.0703, 0.0927	0.0865, 0.1498
GooF	1.018	1.028
largest diff. Fourier peak, hole (eÅ^{-3})	0.597, -0.483	0.210, -0.236

Table S2 Summary of the crystallographic data. Data were collected using Agilent Xcalibur 3 E (**3**) Agilent Xcalibur PX Ultra A (**2**, **2·DMAP**, **S1**), and Bruker Apex II (**4**) diffractometers, and the structures were solved and refined using the OLEX2[7][8], SHELXTL and SHELX-2018[7,9] program systems. [a] $R_1 = \Sigma||F_o| - |F_c||/\Sigma|F_o|$; $wR_2 = \{\Sigma[w(F_o^2 - F_c^2)^2] / \Sigma[w(F_o^2)^2]\}^{1/2}$; $w^{-1} = \sigma_2(F_o^2) + (aP)^2 + bP$. [b] The molecule has crystallographic C_2 symmetry.

S6.2 Refinement details

S6.2.1 **2**

The crystal of **2** was found to contain half of the molecule in the asymmetric unit, with molecule sitting across a two-fold rotation axis plane passing through Ni1, Al1, C6 and H6. The single crystallographically unique Ni–*μ*-H–Al hydride H1 was found in the Δ*F* map and freely refined (2.0 total hydrides per molecule). The asymmetric unit also contains 1.0 equivalents of *n*-pentane solvent, disordered across a two-fold rotation axis located *ca.* 0.63 Å away from the central carbon. The included solvent molecule was modelled as a single orientation in 0.5 occupancy (and its symmetry-related orientation).

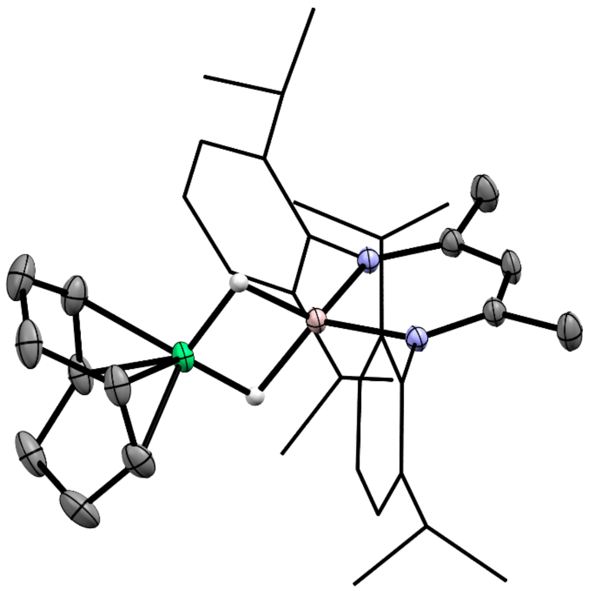

Figure S37 Crystal structure of **2**. Thermal ellipsoids are shown at the 30 % probability level. Most hydrogens and the included solvent molecules are hidden for clarity.

S6.2.2 **2·DMAP**

The crystal of **2·DMAP** was found to be a two-component non-merohedral twin in a *ca.* 0.82:0.18 ratio with the two lattices related by the [1.00 0.00 -0.01 0.01 -1.00 0.00 -0.93 -0.01 -1.00] approximate twin law. The asymmetric unit of **2·DMAP** was found to contain 1.0 equivalent of toluene and 0.5 equivalent of *n*-pentane included solvent. The toluene molecule was modelled in two orientations in a ratio of *ca.* 0.72:0.28, with only the non-hydrogen atoms of the major occupancy orientation modelled anisotropically, the rest were modelled isotropically. The *n*-

pentane was found to be disordered across a centre of inversion located *ca.* 0.36 Å away from the central carbon in one orientation. As modelling it in two orientations (plus their symmetry-related orientations) resulted in occupancies very close to 0.25:0.25, they were ultimately modelled in two orientations (plus their symmetry-related orientations) with their occupancies set to 0.25. Both orientations were modelled anisotropically. For both of the above disorders atoms in the disordered regions were restrained to be similar. The two Ni–μ-H–Al hydrides H1 and H2 were found in the ΔF map and freely refined. The COD ligand likely exhibits disorder as evidenced by the elongated thermal ellipsoids. Attempts to model this did not result in significant improvements in the structure.

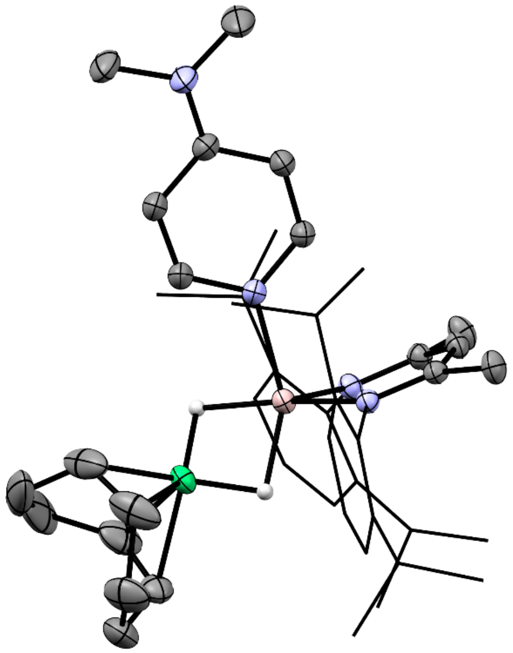

Figure S38 Crystal structure of **2·DMAP**. Thermal ellipsoids are shown at the 30 % probability level. Most hydrogens and the included solvent molecules are hidden for clarity.

S6.2.3 **3**

The Ni–μ-H–Al hydride H1 was found in the ΔF map and freely refined.

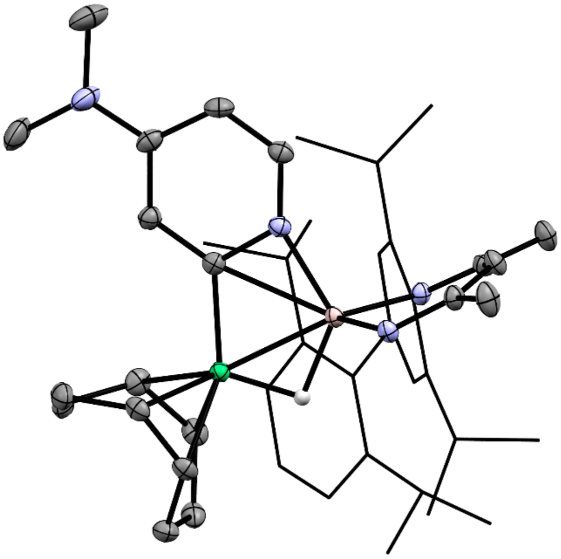

Figure S39 Crystal structures of **3**. Thermal ellipsoids are shown at the 30 % probability level. Most hydrogens are hidden for clarity.

S6.2.4 **4**

The Ni–μ-H–Al hydride H1 was found in the ΔF map and freely refined.

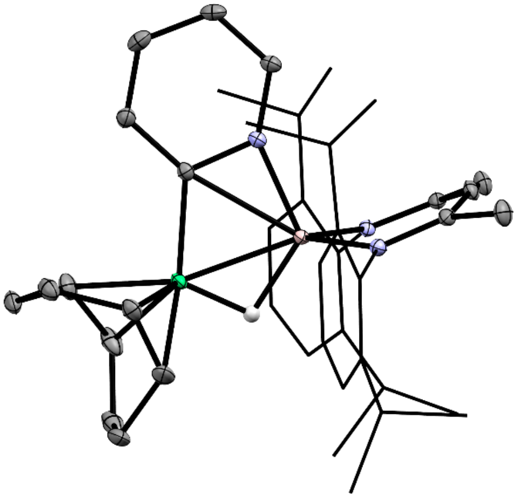

Figure S40 Crystal structure of **4**. Thermal ellipsoids are shown at the 30 % probability level. Most hydrogens are hidden for clarity.

S6.2.5 **S1**

The Al–H hydride H1 was found in the Δ*F* map and freely refined. The hydrogen atoms on the former quinoline ring were found in the Δ*F* map, confirming the regioselectivity of the hydroalumination. As free refinement put them very close to the calculated positions, they were ultimately modelled using the conventional riding model.

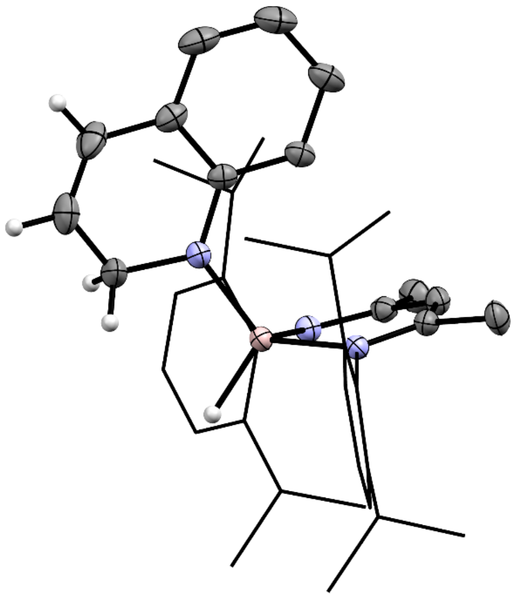

Figure S41 Crystal structure **S1**. Thermal ellipsoids are shown at the 30 % probability level. Most hydrogens are hidden for clarity.

S7 Computational details

S7.1 General computational

Unless otherwise specified, all calculations were performed using ORCA 6.0.0.[10,11]

S7.1.1 Geometry optimisations

Geometry optimisation calculations were performed without symmetry constraints. We employed the r^{2}SCAN-3c composite method developed by the Grimme group.[12] This uses the regularised and restored SCAN *meta*-GGA functional.[13–17] Similarly to other Grimme group "3c" methods, it uses well-balanced approximations to give optimal speed for a reasonable accuracy: the polarised triple-ζ basis set termed def2-mTZVPP (a modified version of def2-TZVP[18]), the atomic-charge dependent D4 dispersion correction[19] and the geometrical counterpoise (gCP)[20] correction to reduce BSSE and BSIE. Solvation was modelled using the Conductor-like Polarizable Continuum Model (CPCM) using toluene solvent. All calculations were performed with the resolution of identity approximation for the Coulomb integrals, and chain of spheres approximation for the exchange integrals (RIJCOSX)[21–23] with the def2/j auxiliary basis set.[24] All calculations were performed using the `defgrid2` integration grid, as is the default in ORCA 6.0.0. Analytical frequency analyses for all stationary points were performed using the enhanced criteria to confirm the nature of the structures as either minima (no imaginary frequency) or transition states (only one imaginary frequency). The vibrational frequencies were not scaled for the thermostatistical corrections. Intrinsic reaction coordinate (IRC) calculations[25] followed by full geometry optimisations on final points were used to connect transition states and minima located on the potential energy surface allowing a full reaction free energy profile to be constructed.

Transition states guess structures were generated primarily using relaxed energy scans or constrained optimisation, either using full r^{2}SCAN-3c, GFN2-xTB, or r^{2}SCAN-3c/GFN2-xTB ONIOM methods, or using the NEB method implemented in ORCA 6.0.0. These were then subjected to transition state optimisations including the calculation of a full analytical Hessian at the beginning. For the present system, we found that the use of a hybrid Hessian gave very slow convergence or no convergence at all to the desired transition states.

Generally, optimisations were only attempted for closed shell singlet species. Most C–H activation transition states were reoptimized on the triplet PES. As these were consistently found to be higher in energy than their closed shell counterparts no attempts were made to locate minimal energy crossing points between the singlet and the triplet PESs. No attempts were made to locate C–H activation transition states on the triplet PES other than those we report as singlets (and additional conformers thereof).

Computational geometries are provided as a separate .xyz file. All energies are reported in kcal mol^{-1} unless otherwise specified.

S7.1.2 Single-point corrections

The electronic energies were recomputed using the def2-QZVPP basis set[18] for all atoms, with no effective core potentials. Unless otherwise specified, the TPSS[26] exchange-correlation functional was used. Solvent corrections were applied using the universal solvent model (SMD) using parametrisation for toluene solvent.[27] Dispersion corrections were applied using Grimme's atomic-charge dependent D4 dispersion correction.[19] A correction for the change of state of 1.89 kcal mol^{-1} was added to each species. The thermostatistical corrections used the q-RRHO approximation[28] as is the default in ORCA 6.0.0.

S7.1.3 Conformer searching

For most intermediates, a full conformational search was conducted using the GOAT procedure[29] as included in ORCA 6.0.0, at the GNF2-xTB[30]/ALPB(toluene)[31] level of theory, as part of the xTB 6.7.1 package bundled with ORCA 6.0.0. The use of GFN-FF[32] by itself gave significant speed-ups, but was found to give a qualitatively incorrect model for the bridging hydrides of the system for some intermediates. For the most conformationally flexible species a GNF2-xTB/GFN-FF/ALPB(toluene) hybrid method was used (using the `GFNUPHILL GFNFF` keyword combination). Transition states and some intermediates necessitated key geometric parameters to be constrained. For each intermediate, at least the 50 lowest energy conformers were reoptimized with DFT. For transition states, optimisations were initiated from multiple guess structures, including ones closely resembling the lowest energy conformer of the

intermediates on both side of the TS. Unless otherwise specified, only the lowest energy conformer of each species is presented.

S7.1.4 Wavefunction analyses

Unless otherwise specified, the level of theory used for single point corrections was used for wavefunction analyses.

NBO analysis was performed using NBO 7.0.10[33] as interfaced with ORCA 6.0.1. The level of theory used for single point correction was used.

QTAIM analyses were performed using the AIMAll package (version 17.01.25)[34] or using Multiwfn 3.8dev.[35,36] The level of theory used for single point correction was used.

FOD analysis[37] was performed in ORCA 6.0.0, as defined by the `FOD` keyword. This means, the TPSS functional, the def2-TZVP basis set and an electronic temperature of T_{el} = 5000 K were used. FOD analysis was performed using the same implicit solvent model as single point calculations (SMD, toluene).

Being disabled in ORCA 6.0.0 and 6.0.1, ETS-NOCV analysis[38] was performed using ORCA 5.0.4[39] and Multiwfn 3.8dev.[35,36] The level of theory used for single point correction was used.

Mayer Bond Indices (MBIs) and MBIS charges[40] were calculated using ORCA 6.0.1. The level of theory used for single point correction was used. Atomic contributions were obtained from a subsequent Löwdin population analysis.

IBO analysis[41] was performed in ORCA 6.0.0. The level of theory used for single point correction was used. Atomic contributions were obtained from a subsequent Löwdin population analysis.

ELF analysis was performed using Multiwfn 3.8dev.[35,36] The level of theory used for single point correction was used.

S7.1.5 The treatment of H_2

To better match experiments, H_2 was treated as an unsolvated gas. This means that it was optimised, and its energy calculated without the use of an implicit solvation model and without the change of state correction to the Gibbs free energy (*vide infra* for details).

S7.2 Computational benchmarking

S7.2.1 Benchmarking methodology

Complexes **1**, **2**, **2·DMAP** and **3**, [Ni(COD)$_2$], DMAP, COD and H_2 were optimized and subjected to single-point corrections using various computational methods.

Most of the benchmarking took place before full conformational analysis was performed, so the geometries used here do not exactly match the final geometries. For several methods, we also report the energies for the conformers we ultimately determined to be most stable (marked with **conf**). For all other methods, optimisations were performed starting from the same geometries (solid state geometries in case of **2**, **2·DMAP** and **3**), which were ultimately found to be close in energy to the most stable conformer.

The following assessment criteria were used:

- According to our van't Hoff analysis, ΔG(**2**) = 2.9 kcal mol^{-1}. For a system of this size and complexity, accounting for errors, the computed ΔG(**2**) = 2.9±2.0 kcal mol^{-1}. Methods that gave a qualitatively incorrect result (predicted the formation of **2** to be exergonic or only very slightly endergonic) were in general not followed up further.
- The addition of DMAP to **2** resulted in the formation of **2·DMAP** in high yield, so ΔG(**2·DMAP**) < ΔG(**2**).
- C–H activation from **2·DMAP** is spontaneous but the reaction is reversible in the presence of excess H_2. Consequently, ΔG(**3**) < ΔG(**2·DMAP**). As a model for the reversibility, ΔG(**3**) < 0 kcal mol^{-1} when H_2 is treated as an unsolvated gas and 0 < ΔG(**3**) when H_2 is treated as a solute. To aid interpretation and better reflect experiments, we report all energies where H_2 is formed with H_2 being treated as an unsolvated gas.

For optimisations, the following hybrid basis set was used, unless otherwise specified or the basis set definition is part of a composite method: def2-SVP(C,H)/def2-TZVP(-f)(N)/def2-TZVPP(Ni,Al). The D4 dispersion correction was applied to all DFT methods unless a different method is part of the definition of the composite method. Most optimisations were performed using the CPCM implicit solvation correction for toluene solvent.

Single point corrections were performed with the following basis sets: QZ = def2-QZVPP, ma-TZ = ma-def2-TZVPP, TZ = def2-TZVPP. The D4 dispersion correction was used for all DFT methods unless no parametrisation was available; in this case D3(BJ) or D2 were used instead. All single point corrections used the SMD solvent model for toluene solvent. For double hybrid functionals, the numbers outside the parentheses were computed using the frozen core approximation (`frozencore` keyword, default), the values inside the parentheses were computed with the frozen core approximation turned off (`nofrozencore` keyword). The frozen core approximation was always applied for coupled cluster methods.

S7.2.2 Thermochemistry benchmarking results

Optimisation	Single point	ΔG(**2**)	ΔG(**2·DMAP**)	ΔG(**3**)
r^2SCAN-3c/CPCM(toluene)		+4.4	+1.0	-0.7
	r^2SCAN-D4/QZ	+1.4	-0.5	-0.9
	**r^2SCAN-D4/QZ/conf**	**+1.1**	**-0.8**	**-1.2**
	SCAN-D4/QZ	+1.4	-0.8	-0.8
	SCAN-D4/QZ/**conf**	+1.1	-1.1	-1.2
	RSCAN-D4/QZ	+0.8	-1.7	-1.0
	RSCAN-D4/QZ/**conf**	+0.5	-2.0	-1.3
	r^2SCANh-D4/QZ	+0.5	-1.0	-0.3
	r^2SCAN0-D4/QZ	-0.6	-1.6	+0.7
	r^2SCAN50-D4/QZ	-2.5	-2.6	+1.9
	ωr^2SCAN-D4/QZ	-2.7	-4.6	-1.3
	Pr^2SCAN50-D4/TZ	+3.4 (+3.5)	+0.2 (-0.1)	-0.6 (-1.0)
	Pr^2SCAN50-D4/ma-TZ	+3.2 (+3.2)	+0.1 (-0.4)	-0.7 (-1.2)
	Pr^2SCAN50-D4/QZ	+3.6 (+3.5)	+1.3 (+0.6)	+0.3 (-0.4)
	Pr^2SCAN69-D4/TZ	+5.9 (+6.0)	+3.2 (+2.6)	+1.7 (+1.0)
	Pr^2SCAN69-D4/ma-TZ	+5.7 (+5.7)	+2.8 (+2.0)	+1.4 (+0.6)
	Pr^2SCAN69-D4/QZ	+6.2 (+6.1)	+4.6 (+3.3)	+3.0 (+1.8)
	κPr^2SCAN50-D4/TZ	+2.5 (+2.6)	-1.0 (-1.4)	-1.6 (-2.0)
	**κPr^2SCAN50-D4/TZ/conf**	+2.6	-1.1	-1.5
	κPr^2SCAN50-D4/ma-TZ	+2.3 (+2.3)	-1.3 (-1.8)	-1.7 (-2.3)
	κPr^2SCAN50-D4/QZ	+2.7 (+2.6)	+0.1 (-0.8)	-0.6 (-0.4)
	ωPr^2SCAN50-D4/TZ	+2.5 (+2.6)	-1.6 (-2.1)	-1.6 (-2.2)
	ωPr^2SCAN50-D4/ma-TZ	+2.3 (+2.3)	-1.9 (-2.5)	-1.8 (-2.5)
	ωPr^2SCAN50-D4/QZ	+2.7 (+2.7)	-0.4 (-1.4)	-0.5 (-1.5)
	TPSS-D4/QZ	+4.2	+0.6	-0.4
	**TPSS-D4/QZ/conf**	**+4.0**	**+0.3**	**-0.7**
	revTPSS-D4/QZ	+9.2	+4.7	+2.8
	revTPSS-D4/QZ/**conf**	+9.0	+4.5	+2.5
	TPSSh-D4/QZ	+3.1	0.0	+0.3
	TPSS0-D4/QZ	+2.0	-0.3	+1.9

Table S3 Thermochemistry benchmarking results.

Optimisation	Single point	ΔG(**2**)	ΔG(**2·DMAP**)	ΔG(**3**)
r²SCAN-3c/CPCM(toluene)	ωB97M-D4rev/QZ	-2.8	-3.4	-1.3
	ωB97M-V/QZ	-1.8	-4.1	-1.9
	B97M-D4/QZ	+3.6	+2.7	+6.4
	X3LYP-D4/QZ	-3.8	-2.1	-2.3
	PWPB95-D4/QZ	+4.6	+0.7	+2.3
	PW6B95-D4/QZ	+1.1	-0.9	+3.0
	PW1PW-D4/QZ	-1.0	-0.6	+1.9
	O3LYP-D4/QZ	+0.1	-0.3	-1.9
	mPW2PLYP-D4/QZ	+2.4	+3.3	+0.8
	mPW1PW-D4/QZ	-0.9	-2.2	-0.6
	mPW1LYP-D4/QZ	-4.1	-0.9	-0.3
	M06-L-D4/QZ	-0.1	-4.3	+1.0
	M06-D4/QZ	-1.6	-3.4	-2.2
	LC-PBE-D4/QZ	+1.4	-10.1	-4.2
	LC-BLYP-D4/QZ	-2.0	-1.8	+2.3
	CAM-B3LYP-D4/QZ	-4.7	-3.2	-1.3
	BHLYP-D4/QZ	-5.7	-3.1	-0.5
	B3PW-D4/QZ	-1.7	-4.5	-4.1
	B3P-D4/QZ	-2.3	-5.4	-4.5
	B2PLYP-D4/QZ	+3.3	+2.6	-1.4
	B2GP-PLYP-D4/QZ	+4.6	+4.6	+0.8
	B1P-D4/QZ	-3.2	-5.9	-4.4
	REVPBE0-D4/QZ	+1.4	-0.2	+2.2
	DSD-PBEB95-D3(BJ)/QZ	+5.3 (+5.2)	+0.3 (-0.7)	+2.4 (+1.4)
	DSD-PBEP86-D3(BJ)/QZ	+7.5 (+7.4)	+5.5 (+4.1)	+4.6 (+3.4)
	PBE0-DH-D3(BJ) /QZ	+0.1 (+0.1)	-8.0 (-8.4)	-4.7 (-5.1)
	PBE-QIDH-D3(BJ) /QZ	+5.9 (+5.8)	+4.6 (+3.6)	+5.5 (+4.5)
	revDOD-PBEP86-D4-2021/QZ	+7.0 (+6.9)	+6.7 (+5.4)	+6.9 (+5.7)
	revDSD-PBEP86-D4-2021/QZ	+7.2 (+7.1)	+7.0 (+5.7)	+6.8 (+5.6)
	RSX-0DH-D2/QZ	+8.1 (+8.1)	-20.3 (-20.7)	-10.3 (-10.6)
	RSX-QIDH-D2/QZ	+11.6 (+11.5)	-19.7 (-20.7)	-12.7 (-13.6)

Table S3 continued. Thermochemistry benchmarking results.

Optimisation	Single point	ΔG(**2**)	ΔG(**2·DMAP**)	ΔG(**3**)
r²SCAN-3c/CPCM(toluene)	SOS-PBE-QIDH-D3(BJ) /QZ	+5.9 (+5.8)	+4.6 (+3.6)	+5.5 (+4.5)
	SOS-RSX-QIDH-D2/QZ	+11.6 (+11.5)	-19.7 (-20.7)	-12.7 (-13.6)
	SOS-ωPBEPP86-D2/QZ	+15.5 (+15.3)	-27.2 (-28.6)	-21.3 (-22.6)
	DLPNO-CCSD(T)/TZ	0.0		
r²SCAN-3c		+6.5	+3.0	-1.7
	r²SCAN-D4/QZ	+1.3	-0.5	-0.8
	r²SCANh-D4/QZ	+0.4	-1.0	-0.2
	r²SCAN0-D4/QZ	-0.8	-1.7	+0.8
	r²SCAN50-D4/QZ	-2.8	-2.8	-2.0
	ωr²SCAN-D4/QZ	-2.9	-4.7	-1.3
	Pr²SCAN50-D4/TZ	+3.9	+1.6	+0.7
	ωPr²SCAN50-D4/TZ	+3.1	-0.1	-0.2
	κPr²SCAN50-D4/TZ	+2.9	+0.3	-0.3
	TPSS-D4/QZ	+4.1	+0.4	-0.4
	TPSSh-D4/QZ	+3.0	-0.2	+0.4
	TPSS0-D4/QZ	+1.9	-0.5	+1.9
	B2PLYP-D4/QZ	+3.3	+2.8	-1.4
	ωB97XM-D4rev/QZ	-1.6		
	REVPBE0-D4/QZ	+0.9		
TPSSh-D4/vDZP/CPCM(tol)		+6.0	-0.8	-0.3
	TPSSh-D4/QZ	+3.2	-0.6	0.0
	TPSS0-D4/QZ	+2.3	-1.0	+1.5
TPSSh-D4/vDZP		+7.3	+0.3	-2.0
	TPSSh-D4/QZ	+3.3	-0.2	+0.6
	TPSS0-D4/QZ	+2.3	-0.6	+1.8
TPSS0-D4/vDZP/CPCM(tol)		+4.7	-2.1	+0.3
	TPSS0-D4/QZ	+2.5	-0.6	+1.6
	TPSSh-D4/QZ	+3.4	-0.3	0.0
TPSS0-D4/vDZP		+6.0	-0.7	-1.4
	TPSS0-D4/QZ	+2.6	-0.3	+1.8
	TPSSh-D4/QZ	+3.5	+0.1	+0.2

Table S3 continued. Thermochemistry benchmarking results.

Optimisation	Single point	ΔG(**2**)	ΔG(**2·DMAP**)	ΔG(**3**)
TPSS-D4/vDZP/CPCM(tol)		+7.3	+0.4	-0.5
	TPSS-D4/QZ	+4.2	-0.1	-0.6
	TPSSh-D4/QZ	+3.0	-0.9	0.0
	TPSS0-D4/QZ	+1.9	-1.4	+1.4
TPSS-D4/vDZP		+8.7	+1.5	-2.2
	TPSS-D4/QZ	+4.4	+0.3	-0.4
	revTPSS-D4/QZ	+9.5	+4.5	+3.0
	TPSSh-D4/QZ	+3.2	-0.5	+0.3
	TPSS0-D4/QZ	+2.0	-1.0	+1.7
BP86-D4/TZ/CPCM(tol)		+6.0		
	BP86-D4/QZ	-6.6		
	B3P-D4/QZ	-0.3		
BP86-D4/TZ		-0.6		
	B3P-D4/QZ	-3.5		
B3P-D4/CPCM(tol)		0.0		
	B3P-D4/QZ	-3.5		
PBEh-3c/CPCM(tol)		+8.3		
	REVPBE0-D4/QZ	+1.5		
	DLPNO-CCSD(T)/TZ	+0.2		
CAM-B3LYP-D4/CPCM(tol)		-0.3		
	CAM-B3LYP-D4/QZ	-4.8		
REVPBE0-D4/CPCM(tol)		+4.2		
	REVPBE0-D4/QZ	+1.2		
ωB97X-3c		-0.5		
	ωB97M-D4rev/QZ	-2.9		
	DLPNO-CCSD(T)/TZ	+0.6		

Table S3 continued. Thermochemistry benchmarking results.

S7.2.3 Summary of thermochemistry benchmarking results

Most density functionals gave a *qualitatively incorrect* result for **2**, as in ΔG(**2**) < 0 kcal/mol. In general, optimisation and single point corrections with pure *m*GGA functionals provided reasonable results. Results deteriorated upon the inclusion of exact exchange, with functionals incorporating a significant admixture of HF exchange giving high errors. This is especially true for range-separated hybrids with 100 % Fock exchange in the long-range regime. Most double hybrid density functionals (DHDFs) in general gave erratic energies. The frozen core approximation did not appear to introduce significant *additional* errors. Certain DHDFs appeared to be able to correct for the errors introduced by the inclusion of exact exchange with exact correlation, however, the approximations of the perturbation theory appeared to break down for structures further from the test set (transition states and intermediates later along the PES). A possible explanation for these results is the presence of significant static electron correlation and corresponding local multireference character (*vide infra* for FOD analysis).

In the end, the TPSS-D4/def2-QZVPP/SMD(toluene)//r^{2}SCAN-3c/CPCM(toluene) level of theory was chosen. While we acknowledge somewhat reduced accuracy of *m*GGA functionals compared to hybrids, their local nature gave us confidence in the overall reliability and balanced errors for the present system.

S7.2.4 Comparison of computed and crystal structures

Below are comparisons of structural parameters for **2**, **2·DMAP**, **3**, **4** and **S1**. All bond lengths are given in Å. Metal-hydride distances are systematically underestimated by X-ray crystallography under the IAM approximation. For the computed structures the lowest energy conformers were used.

Method	X-ray	DFT [r^{2}SCAN-3c/CPCM(tol)]
Ni---Al	2.1953(10)	2.194
Ni---H	1.54(5), 1.54(5)	1.655, 1.652
Al----H	1.61(4), 1.61(4)	1.699, 1.701
Ni---C(COD)	2.058(4), 2.058(4), 2.066(3), 2.066(3)	2.065, 2.065, 2.071, 2.073
Al---N(BDI)	1.880(2), 1.880(2)	1.884, 1.885

Table S4 Comparison of crystallographic and computed structural parameters for **2**.

Method	X-ray	DFT [r^{2}SCAN-3c/CPCM(tol)]
Ni---Al	2.2606(14)	2.248
Ni---H	1.49(13), 1.55(5)	1.596, 1.572
Al----H	1.66(14), 1.75(5)	1.790, 1.817
Ni---C(COD)	2.008(7), 2.022(8), 2.062(7), 2.096(6)	2.029, 2.043, 2.093, 2.110
Al---N(DMAP)	2.038(4)	2.044
Al---N(BDI)	1.985(4), 1.939(4)	1.949, 1.977

Table S5 Comparison of crystallographic and computed structural parameters for **2·DMAP**.

Method	X-ray	DFT [r^{2}SCAN-3c/CPCM(tol)]
Ni---Al	2.3188(7)	2.317
Ni---H	1.53(2)	1.592
Al----H	1.65(2)	1.716
Ni---C(DMAP)	1.945(2)	1.951
Ni---C(COD)	2.059(2), 2.077(2), 2.111(2), 2.118(2)	2.074, 2.092, 2.099, 2.110
Al---N(DMAP)	1.8818(19)	1.886
Al---N(BDI)	1.9004(19), 1.9207(19)	1.908, 1.920

Table S6 Comparison of crystallographic and computed structural parameters for **3**.

Method	X-ray	DFT [r^{2}SCAN-3c/CPCM(tol)]
Ni---Al	2.3041(6)	2.309
Ni---H	1.52(2)	1.596
Al----H	1.65(2)	1.716
Ni---C(Py)	1.9367(18)	1.936
Ni---C(COD)	2.053(2), 2.084(2), 2.105(2), 2.1071(19)	2.080, 2.091, 2.106, 2.112
Al---N(Py)	1.8916(15)	1.904
Al---N(BDI)	1.9003(15), 1.9061(15)	1.907, 1.909

Table S7 Comparison of crystallographic and computed structural parameters for **4**.

Method	X-ray	DFT [r^{2}SCAN-3c/CPCM(tol)]
Al----H	1.53(2)	1.593
Al---N(Quin)	1.837(2)	1.858
Al---N(BDI)	1.8899(19), 1.903(2)	1.899, 1.905
N---CH_2(Quin)	1.469(3)	1.484
N---C(Quin)	1.382(3)	1.379

Table S8 Comparison of crystallographic and computed structural parameters for **S1**.

S7.3 Computed stationary points

S7.3.1 Full computed potential energy surface for the PCy3-catalysed C–H activation of DMAP by **2**

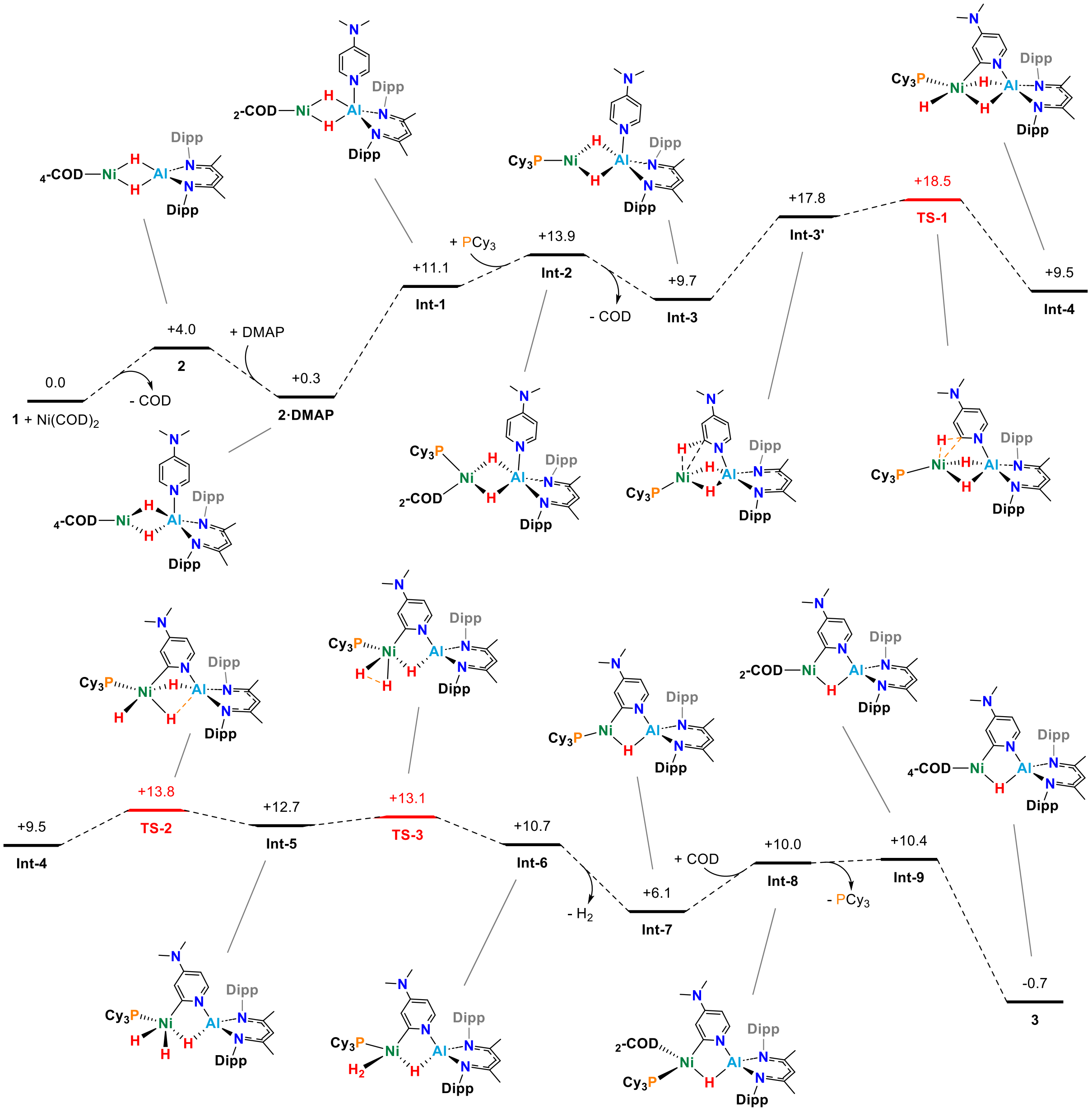

Figure S42 Full computed PES for the C–H activation of DMAP by **2** at the TPSS-D4/def2-QZVPP/SMD(toluene)//r²SCAN-3c/CPCM(toluene) level of theory. Gibbs free energies in kcal mol⁻¹, reported with respect to **1** + [Ni(COD)2].

S7.3.2 Energies of all computed stationary points

Gibbs free energies are reported with respect to **1** + [Ni(COD)$_2$] outside of the brackets, with respect to **2·DMAP** (in parentheses) or **2·Py** [in square brackets].

**Species**	**G(r²SCAN-3c) / eH**	**E(r²SCAN-3c) / eH**	**E(TPSS-D4/QZ) / eH**	**ΔG / kcal mol⁻¹**	**Imaginary frequency / cm⁻¹**
**1**	-1482.08529281	-1482.67234843282	-1483.94655472681	0.0 (-0.3)	
[Ni(COD)$_2$]	-2131.98587786	-2132.30718673458	-2133.04424812546		
COD	-311.78895435	-311.93678405350	-312.25197922399		
PCy$_3$	-1046.51210990	-1046.94757507858	-1047.73153433822		
H_2	-1.17127655	-1.16938164685	-1.18059091561		
DMAP	-382.03018486	-382.15782520205	-382.52431835474		
pyridine	-248.16227242	-248.22309685357	-248.45550928715		
quinoline	-401.73297783	-401.83666026639	-402.20777518245		
**2**	-3302.27455539	-3303.03482672620	-3304.73224332087	+4.0 (+3.7)	
**2·DMAP**	-3684.30716241	-3685.22421558216	-3687.28853804308	+0.3 (0.0)	
**Int-1**	-3684.28903726	-3685.20318974200	-3687.26841224507	+11.1 (+10.8)	
**Int-2**	-4730.79431564	-4732.17538259982	-4735.02397959677	+13.9 (+13.6)	
**Int-3**	-4419.00969879	-4420.21054531914	-4422.74922800350	+9.7 (+9.4)	
**Int-3′**	-4418.99970700	-4420.20142123548	-4422.73724835479	+17.8 (+17.5)	
**TS-1**	-4418.99973346	-4420.19942495162	-4422.73415421471	+18.5 (+18.2)	-751
**Int-4**	-4419.01372150	-4420.21411843937	-4422.74906489699	+9.5 (+9.2)	
**TS-2**	-4419.00710872	-4420.20718836149	-4422.74195026055	+13.8 (+13.5)	-228
**Int-5**	-4419.00733421	-4420.20794312358	-4422.74431667112	+12.7 (+12.4)	
**TS-3**	-4419.00881138	-4420.20805903549	-4422.74220809808	+13.1 (+12.8)	-469
**Int-6**	-4419.01341860	-4420.21397846843	-4422.74734367215	+10.7 (+10.4)	
**Int-7**	-4417.84751380	-4419.03285593822	-4421.55699878609	+6.1 (+5.8)	
**Int-8**	-4729.63343888	-4730.99382445148	-4733.82698630760	+14.4 (+14.4)	
**Int-9**	-3683.12233289	-3684.01726006129	-3686.06788196397	+10.4 (+10.1)	
**3**	-3683.13856783	-3684.03544316154	-3686.08741854958	-0.7 (-1.0)	

Table S9 Calculated Gibbs free energies of all stationary points at the TPSS-D4/def2-QZVPP/SMD(toluene)//r²SCAN-3c/CPCM(toluene) level of theory.

Species	G(r^2SCAN-3c) / eH	E(r^2SCAN-3c) / eH	E(TPSS-D4/QZ) / eH	ΔG / kcal mol^{-1}	Imaginary frequency / cm^{-1}
**^{3}TS-1**	-4418.95790303	-4420.154242208873	-4422.71925326417	+25.7 (+25.4)	-678
**TS-1a**	-3684.26038467	-3685.16959186944	-3687.23250226337	+30.5 (+30.2)	-768
**^{3}TS-1a**	-3684.20966413	-3685.11599180724	-3687.21006739866	+42.8 (+42.5)	-588
**TS-1b**	-3684.23238341	-3685.14448384322	-3687.21245344926	+44.9 (+44.6)	-161
**^{3}TS-1b**	-3684.20662171	-3685.11267600355	-3687.18020709840	+61.4 (+61.1)	-446
**TS-1c**	-3684.24108066	-3685.15298867474	-3687.21814288687	+41.2 (+40.8)	-20
**TS-1d**	-3684.22702496	-3685.13518168666	-3687.19783668123	+51.6 (+61.3)	-797
**TS-1e**	-3683.08833649	-3683.98503603665	-3686.03468286228	+32.3 (+32.0)	-552
**^{3}TS-1e**	-3683.05579422	-3683.94905613938	-3686.01021968426	+45.5 (+45.2)	-373
**2·Py**	-3550.43295480	-3551.28214636903	-3553.21285079997	+4.0 [0.0]	
**TS-1-Py**	-4285.12970429	-4286.26145576156	-4288.66193563126	+19.9 [+15.5]	-734
**Int-4-Py**	-4285.14337797	-4286.27652186563	-4288.67723976693	+11.2 [+7.2]	
**TS-3-Py**	-4285.13905942	-4286.27068030275	-4288.67087445983	+14.2 [+10.2]	-430
**4**	-3549.26985764	-3550.09888434580	-3552.01671533244	-0.1 [-4.1]	
**1·DMAP**	-1864.10507852	-1864.84694767852	-1866.48930254749	+3.6 (+3.3)	
**S1-DMAP**	-1864.10907142	-1864.85418502320	-1866.49113040419	+4.5 (+4.2)	
**S1-Py**	-1730.24811732	-1730.92432957516	-1732.42817098583	-0.5 [-4.5]	
**S1**	-1883.83466647	-1884.55526963522	-1886.19707171996	-10.0	

Table S9 continued. Calculated Gibbs free energies of all stationary points at the at the TPSS-D4/def2-QZVPP/SMD(toluene)//r^2SCAN-3c/CPCM(toluene) level of theory.

Species	E(r^2SCAN-D4/QZ) / eH	ΔG / kcalmol^{-1}	E(κPr^{2}SCAN50-D4/TZ)/ eH	ΔG / kcalmol^{-1}
**1**	-1482.84948388240	0	-1482.10627008395	0
[Ni(COD)$_2$]	-2132.43269718291		-2131.85939038134	
COD	-311.98134091461		-311.83121057790	
PCy$_3$	-1047.07139220140		-1046.60968941785	
H_2	-1.17159651612		-1.17373578336	
DMAP	-382.21437382923		-382.02011333715	
pyridine	-248.25471163557		-248.12356913090	
quinoline	-401.88439290793		-401.66693806690	
**2**	-3303.29875464912	+1.2	-3302.13010741163	+2.6
**2·DMAP**	-3685.54232898883	-0.8	-3684.18221966223	-1.1

Table S10 Calculated Gibbs free energies using alternative functionals.

**Species**	**E(r²SCAN-D4/QZ) / eH**	**ΔG / kcalmol-1**	**E(κPr²SCAN50-D4/TZ)/ eH**	**ΔG / kcalmol-1**
**Int-1**	-3685.52470694672	+8.5	-3684.15786252732	+12.3
**Int-2**	-4732.61534946463	+14.1	-4730.79318709196	+14.2
**Int-3**	-4420.61250791442	+9.3	-4418.93775390422	+10.9
**Int-3′**	-4420.60314991661	+15.7	-4418.92609618484	+18.7
**TS-1**	-4420.60061014270	+16.0	-4418.91924186369	+21.8
**Int-4**	-4420.61511141095	+7.4	-4418.92293875055	+19.9
**TS-2**	-4420.60899382450	+11.0	-4418.92155585465	+20.6
**Int-5**	-4420.60984705291	+10.8	-4418.92231844928	+20.4
**TS-3**	-4420.60884681873	+10.6	-4418.92455690181	+18.6
**Int-6**	-4420.61431614633	+8.0	-4418.94106497047	+8.6
**Int-7**	-4419.42583864822	+7.6	-4417.74854233588	+9.4
**Int-8**	-4731.42774972828	+9.8	-4729.59389038601	+15.7
**Int-9**	-3684.33206055196	+10.4	-3682.96236175588	+12.5
**3**	-3684.34885660248	-1.2	-3682.98661476788	-1.5
**³TS-1**	-4420.58367000727	+24.5	-4418.832236548257	+74.3
**TS-1a**	-3685.48758283116	+29.0	-3684.11903168706	+34.0
**³TS-1a**	-3685.46396181981	+41.7	-3684.03064290213	+87.3
**TS-1b**	-3685.46536566928	+44.4	-3684.08638562021	+55.9
**³TS-1b**	-3685.42921911215	+63.3	-3684.03507517398	+84.3
**TS-1c**	-3685.47128371925	+40.6	-3684.11123420060	+40.2
**TS-1d**	-3685.45312785033	+51.8	-3684.08901664067	+49.6
**TS-1e**	-3684.30077855629	+30.2	-3682.93370346965	+32.9
**³TS-1e**	-3684.27157726061	+45.1	-3682.85779105906	+77.1
**2·Py**	-3551.57555053976	+3.0	-3550.27830114883	+2.9
**TS-1-Py**	-4286.63768920326	+17.4	-4285.01735292598	+24.4
**Int-4-Py**	-4286.65248511901	+9.0	-4285.02258098025	+22.0
**TS-3-Py**	-4286.64654906002	+11.7	-4285.02386020599	+20.2
**4**	-3550.38726840681	-0.6	-3549.08770730779	-0.7
**1·DMAP**	-1865.07981316917	+5.2	-1864.14432689227	+3.9
**S1-DMAP**	-1865.08809115298	+2.0	-1864.15524997444	-0.9
**S1-Py**	-1731.13431841659	-5.1	-1730.26327299095	-3.0
**S1**	-1884.77945405874	-11.8	-1883.82391582670	-15.0

Table S10 continued. Calculated Gibbs free energies using alternative functionals.

S7.3.3 Computed transition states for the C–H activation step of DMAP

Gibbs free energies are given with respect to **1** + [Ni(COD)$_2$].

To determine plausible mechanisms for the C–H activation, alternative transition states were also computed for DMAP as the substrate. In accordance with the experimental results, the lowest energy transition state (**TS-1**, $\Delta G^{\ddagger}_{298K}$ = +18.5 kcal mol^{-1}) features a PCy$_3$ ligand (first order in PCy$_3$). The active conformer of **Int-3** in this case is the high energy η^2-agostic complex **Int-3’**.

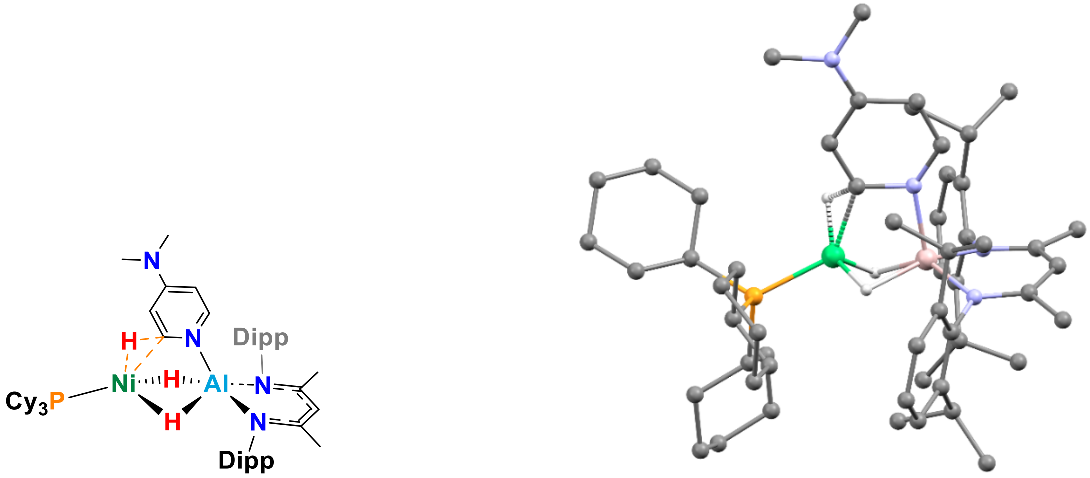

Figure S43 Left: line drawing and right: computed geometry of **TS-1**. Most hydrogens are hidden for clarity. Computed distances: Ni---H: 1.513 Å, C---H: 1.443 Å, Ni---C: 1.931 Å.

On the triplet potential energy surface, a very similar geometry was obtained, but this corresponds to a much higher action energy (^{3}**TS-1**, $\Delta G^{\ddagger}_{298K}$ = +24.5 kcal mol^{-1}).

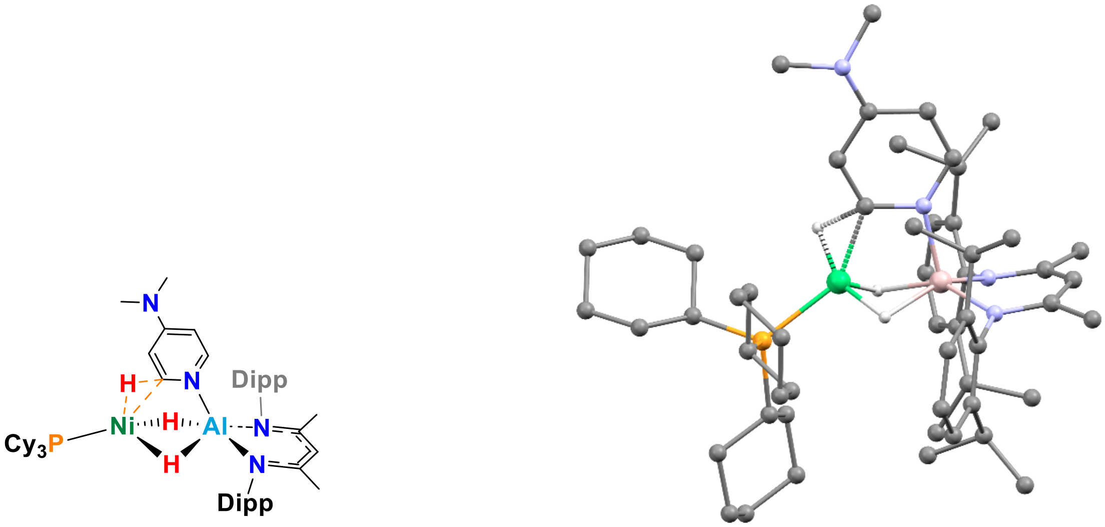

Figure S 44 Left: line drawing and right: computed geometry of ^{3}**TS-1**. Most hydrogens are hidden for clarity. Computed distances: Ni---H: 1.503 Å, C---H: 1.646 Å, Ni---C: 1.943 Å.

In lieu of the phosphine catalyst, the analogous transition state featuring an η^2-COD ligand is significantly higher in energy (**TS-1a**, $\Delta G^{\ddagger}_{298K}$ = +30.5 kcal mol^{-1}).

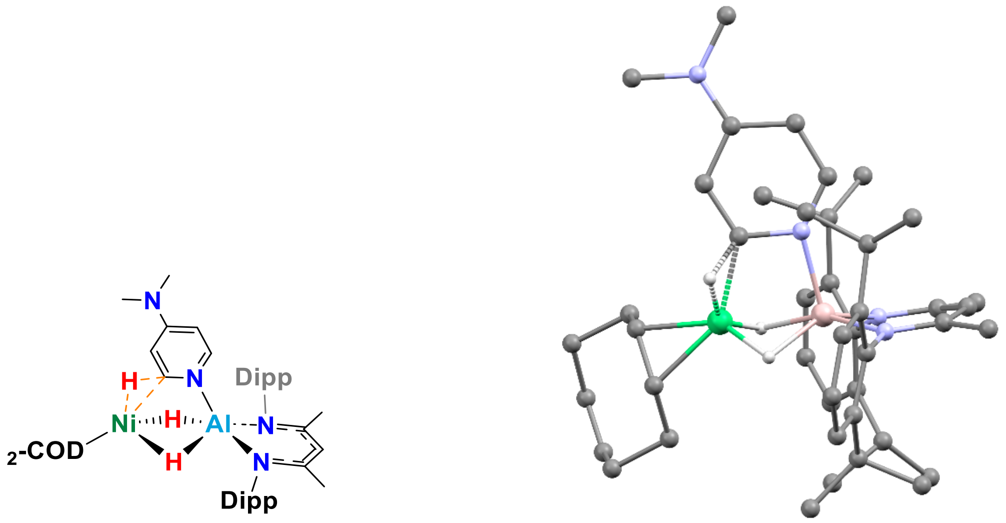

Figure S45 Left: line drawing and right: computed geometry of **TS-1a**. Most hydrogens are hidden for clarity. Computed distances: Ni---H: 1.512 Å, C---H: 1.443 Å, Ni---C: 1.951 Å.

Again, a similar geometry corresponds to a much higher activation energy on the triplet surface (^{3}**TS-1a**, $\Delta G^{\ddagger}_{298K}$ = +42.8 kcal mol^{-1}).

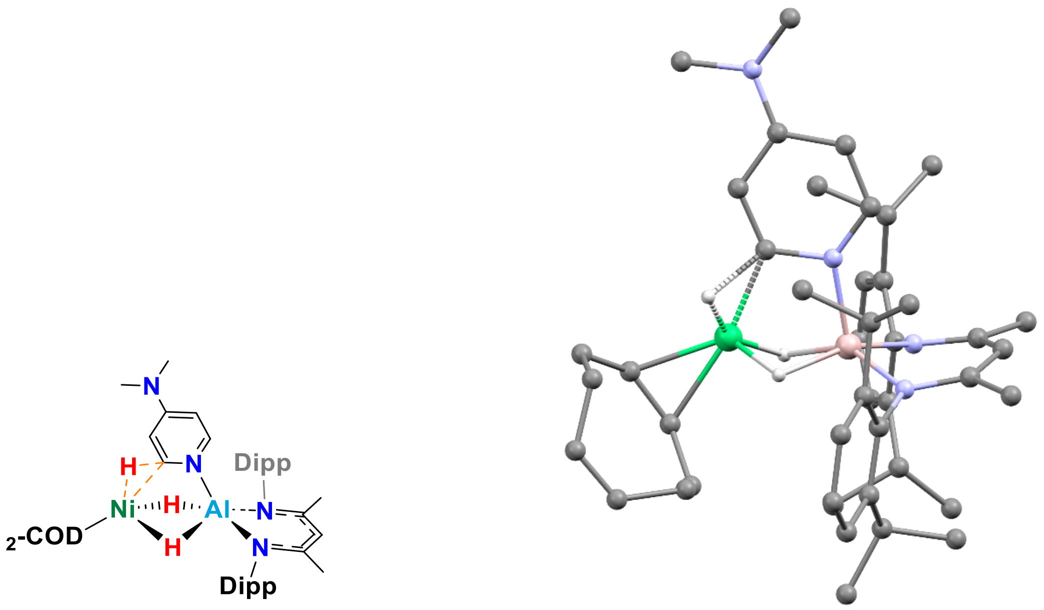

Figure S46 Left: line drawing and right: computed geometry of ^{3}**TS-1a**. Most hydrogens are hidden for clarity. Computed distances: Ni---H: 1.536 Å, C---H: 1.743 Å, Ni---C: 1.966 Å.

When the COD ligand is bound in an η^4 fashion only transition states that are even higher in energy could be found. Analogously to our work on Fe–Al complexes no M–C bond formation was observed in **TS-1b** ($\Delta G^{\ddagger}_{298K}$ = +44.9 kcal mol^{-1}).[42] Again, analogously, **TS-1b** leads to an Al–C-bound isomer, which can then undergo comparatively facile isomerisation.

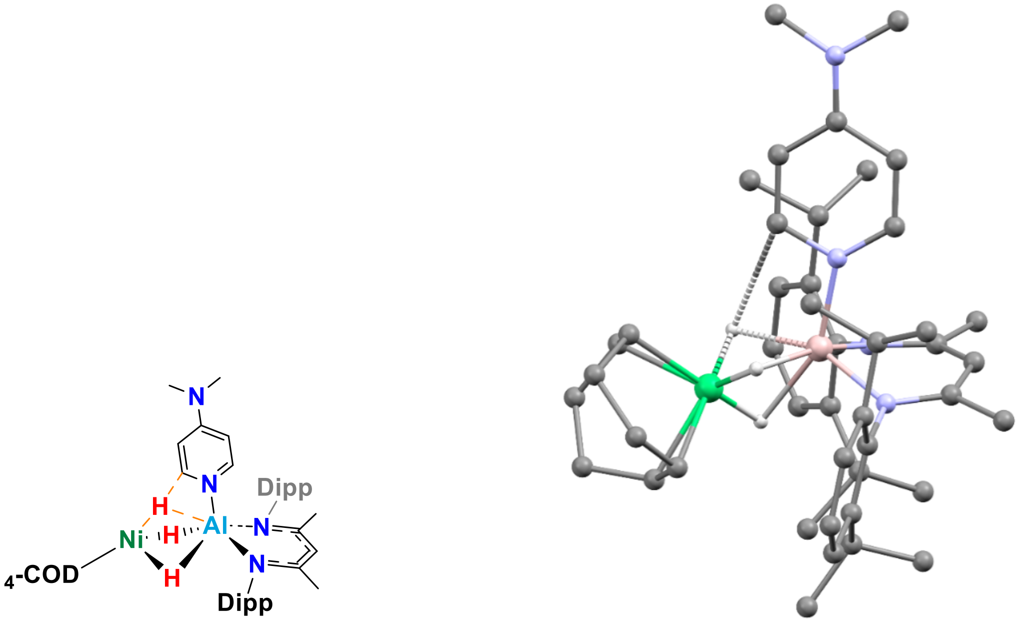

Figure S47 Left: line drawing and right: computed geometry of **TS-1b**. Most hydrogens are hidden for clarity. Computed distances: Ni---H: 1.486 Å, Al---H: 1.961 Å, C---H: 2.650 Å.

This was also found higher in energy on the triplet surface (^{3}**TS-1b**, $\Delta G^{\ddagger}_{298K}$ = +61.4 kcal mol^{-1}).

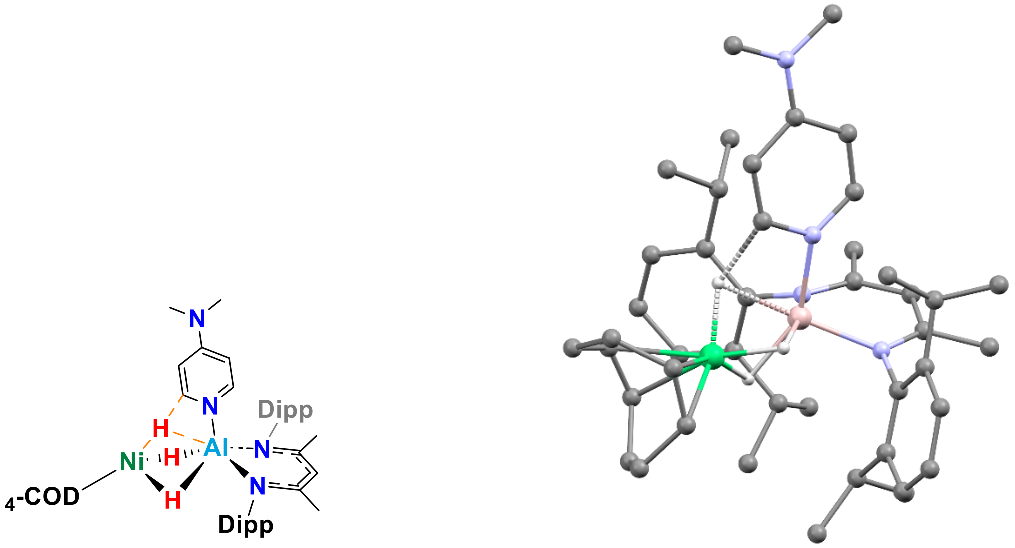

Figure S48 Left: line drawing and right: computed geometry of ^{3}**TS-1b**. Most hydrogens are hidden for clarity. Computed distances: Ni---H: 1.647 Å, Al---H: 2.050 Å, C---H: 2.860 Å.

We also considered that the bridging hydride ligands may act as an internal base, forming a dihydrogen complex directly. This was also found not to be competitive (**TS-1c**, $\Delta G^{\ddagger}_{298K}$ = +41.2 kcal mol^{-1}).

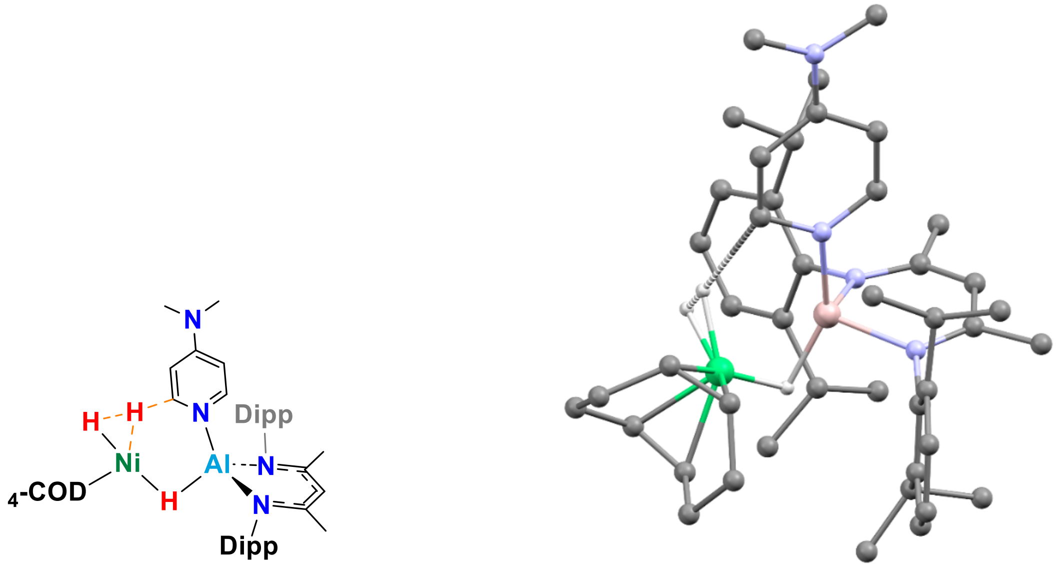

Figure S49 Left: line drawing and right: computed geometry of **TS-1c**. Most hydrogens are hidden for clarity. Computed distances: Ni---H: 1.523 Å, H---H: 0.862 Å, C---H: 2.481 Å.

The possibility of the reductive elimination of dihydrogen preceding the C–H activation was also considered. These pathways correspond to the intermediacy of formally Ni^{0}---Al^{I} species. Two such options were investigated. Firstly, C–H activation may take place after the coupling but before the dissociation of H_2. This is pathway gave the highest energy transition state we computed (**TS-1d**, $\Delta G^{\ddagger}_{298K}$ = +51.6 kcal mol^{-1}).

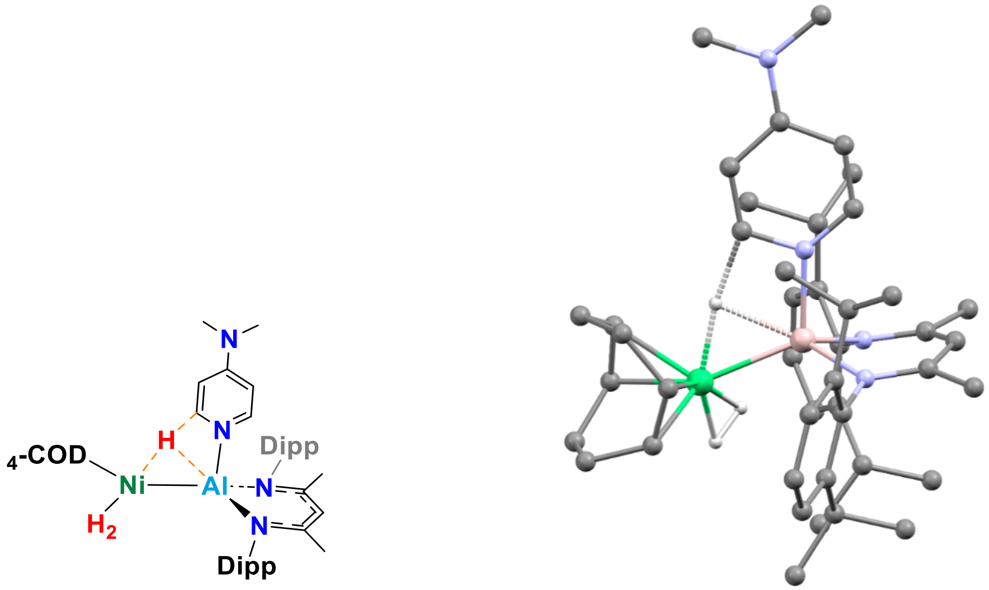

Figure S50 Left: line drawing and right: computed geometry of **TS-1d**. Most hydrogens are hidden for clarity. Computed distances: Ni---H: 1.715 Å, Al---H: 1.975 Å, C---H: 1.680 Å.

Alternatively, C–H activation through an η^2-agostic complex intermediate can also take place after the dissociation of H_2 (**TS-1e**, $\Delta G^{\ddagger}_{298K}$ = +32.3 kcal mol^{-1}).

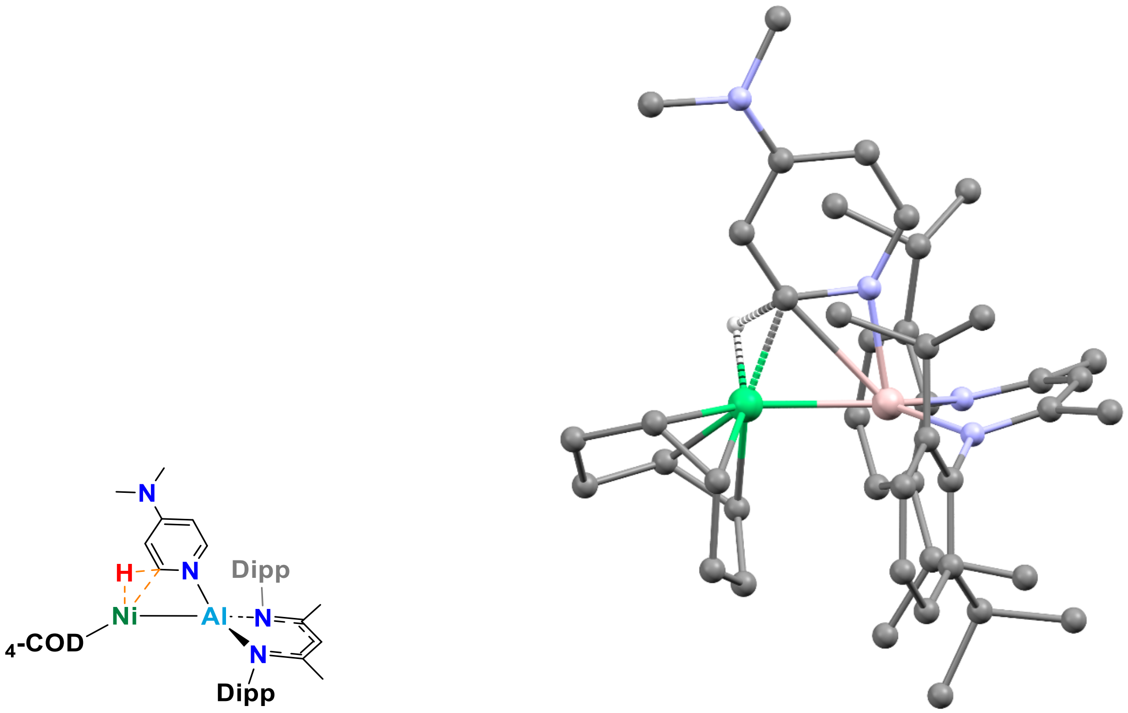

Figure S51 Left: line drawing and right: computed geometry of **TS-1e**. Most hydrogens are hidden for clarity. Computed distances: Ni---H: 1.507 Å, C---H: 1.533 Å, Ni---C: 1.914 Å.

This was also found to be higher in energy on the triplet PES (^{3}**TS-1e**, $\Delta G^{\ddagger}_{298K}$ = +45.5 kcal mol^{-1}).

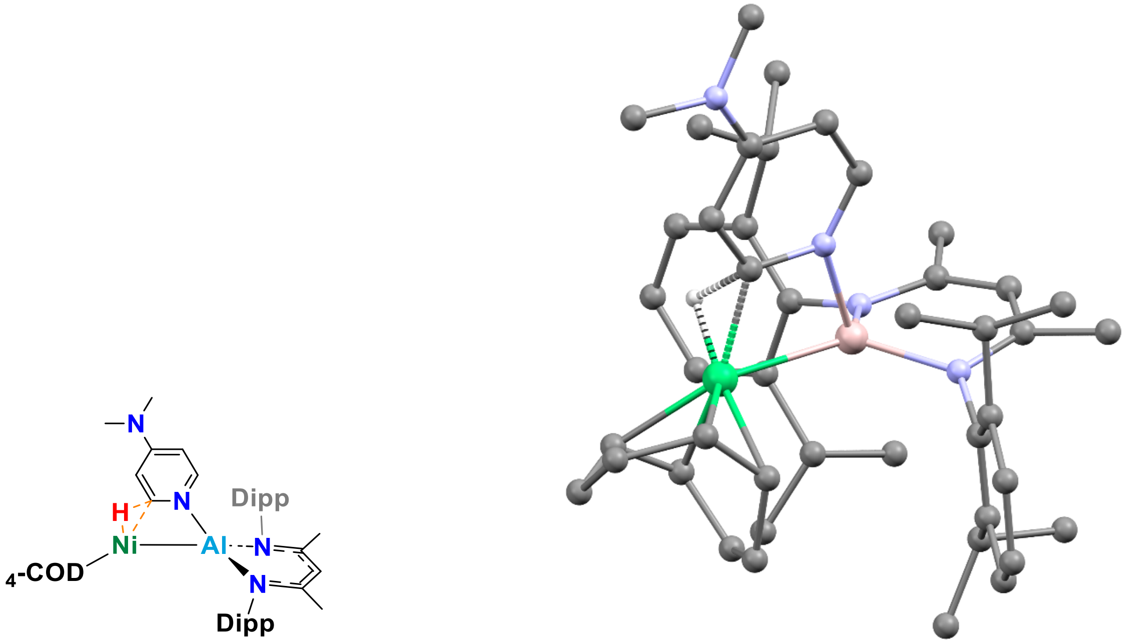

Figure S52 Left: line drawing and right: computed geometry of [3]**TS-1e**. Most hydrogens are hidden for clarity. Computed distances: Ni---H: 1.487 Å, C---H: 1.519 Å, Ni---C: 1.915 Å.

It is worth noting that while the alternative pathways were not computed with a PCy_3 ligand in place of COD, it is unlikely that more accessible PCy_3-ligated analogues exist. Specifically, both for the sequence of **2·DMAP**, **Int-1**, **Int-2**, **Int-3** and for **Int-6**, **Int-7**, **Int-8**, **3**, the thermodynamic stability follows the trend of η^4-COD < PCy_3 < η^2-COD ≈ PCy_3-η^2-COD. Accordingly, **TS-1** is favoured to **TS-1a**, and it is unlikely that PCy_3-analogues of **TS-1b-e** would be competitive.

S7.4 Wavefunction analysis

S7.4.1 Atom labelling

Throughout this section the following atom labelling was used. H^A and H^B originate from **1**, and we consider the reductive elimination of H^BH^C.

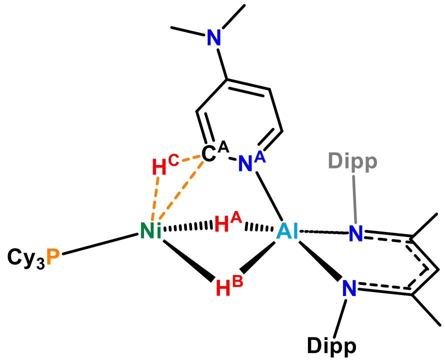

Figure S53 Atom labelling for the structure of **TS-1**.

S7.4.2 FOD analysis

To qualitatively assess the (local) multireference character in these complexes Fractional Occupation number weighted Density (FOD) analysis was performed.[37] Plots of ρ^{FOD} and N^{FOD} values are provided below for key species along the PES. These indicated the presence of significant local multireference character, originating primarily from the donation of electron density from the electron rich Ni centre to the π-system of the BDI ligand and the DMAP/pyridine substrate. This matches our results for the erratic thermochemistry calculated with (double) hybrid functionals and single reference coupled cluster methods. Following the recommendations of the Grimme group, we ultimately utilised pure *m*GGA functionals for the computation of both geometries and energies/densities (*vide supra*).

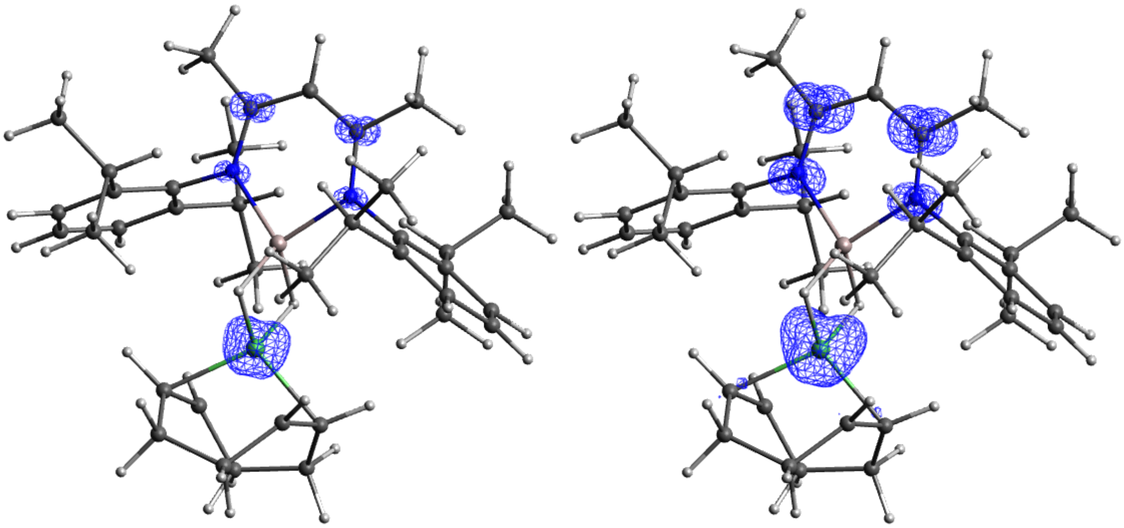

Figure S54 FOD analysis for **2**: left: isovalue = 0.005, right: isovalue = 0.002. N^{FOD} = 0.591.

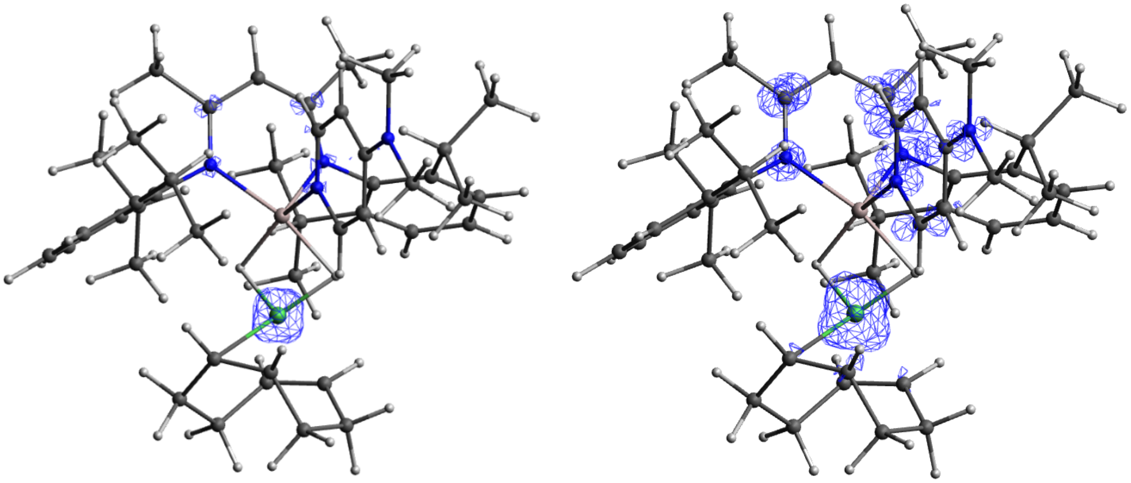

Figure S55 FOD analysis for **2·DMAP**: left: isovalue = 0.005, right: isovalue = 0.002. N^{FOD} = 0.760.

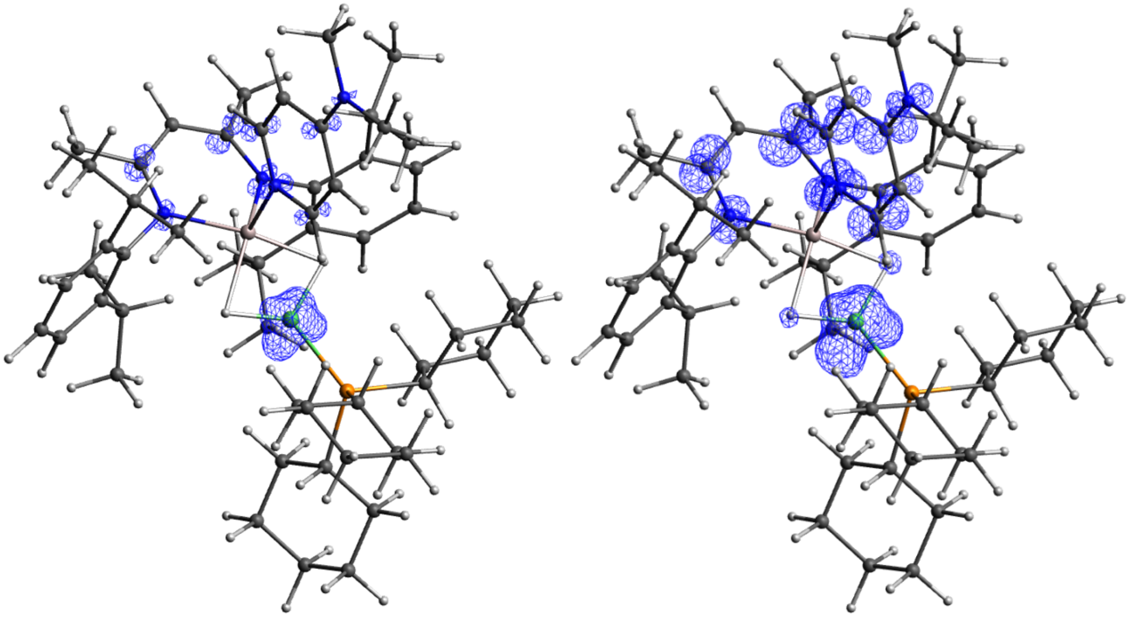

Figure S56 FOD analysis for **Int-3**: left: isovalue = 0.005, right: isovalue = 0.002. N^{FOD} = 1.040.

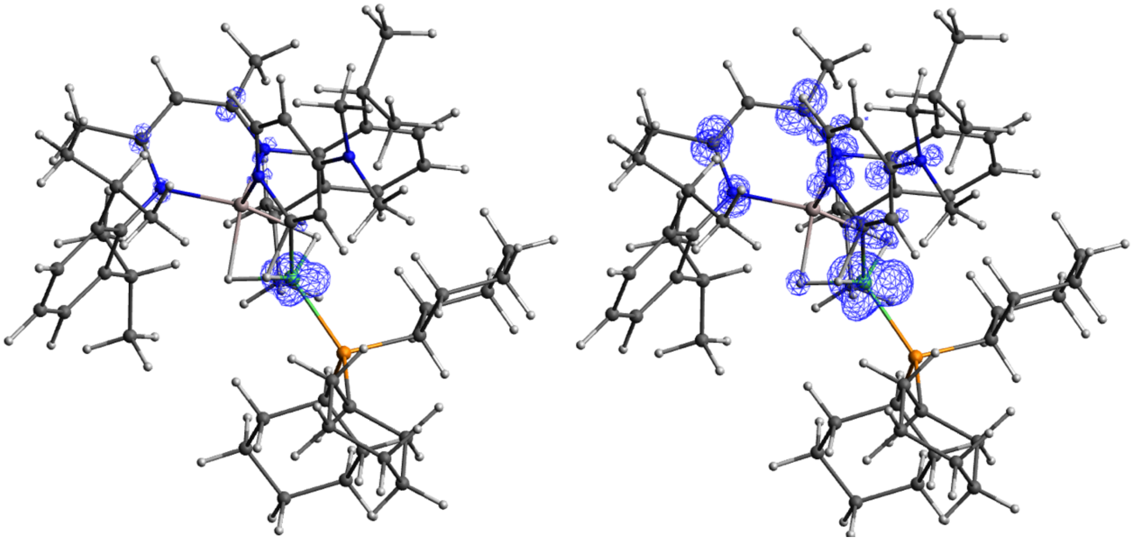

Figure S57 FOD analysis for **TS-1**: left: isovalue = 0.005, right: isovalue = 0.002. N^{FOD} = 0.732.

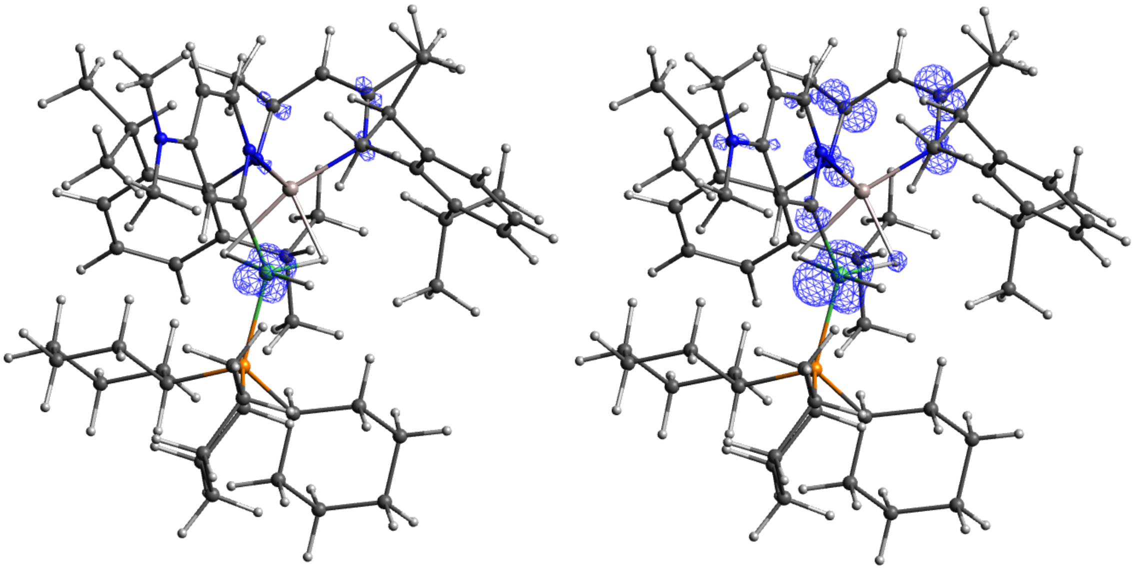

Figure S58 FOD analysis for **Int-4**: left: isovalue = 0.005, right: isovalue = 0.002. N^{FOD} = 0.576.

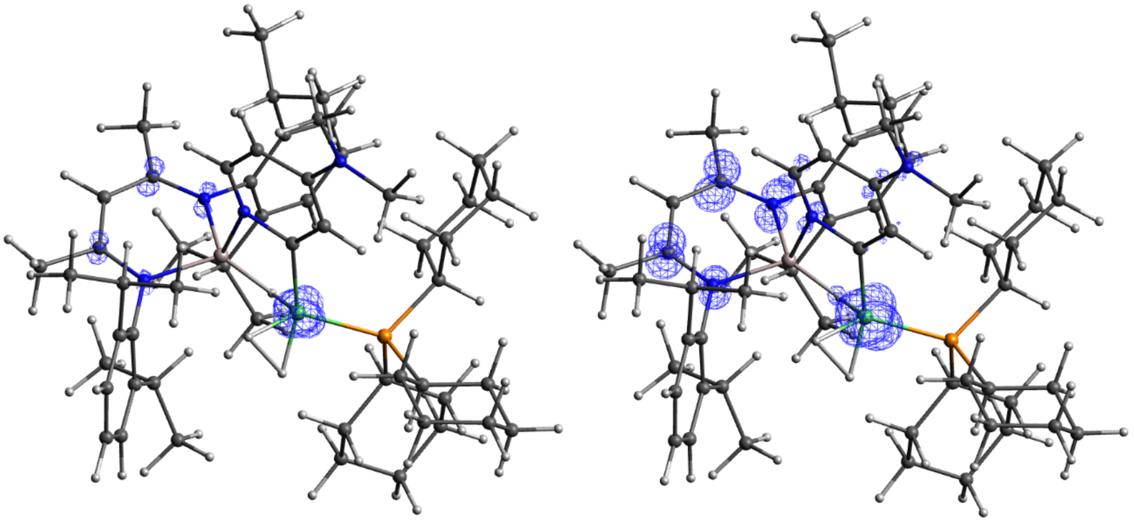

Figure S59 FOD analysis for **TS-2**: left: isovalue = 0.005, right: isovalue = 0.002. N^{FOD} = 0.644.

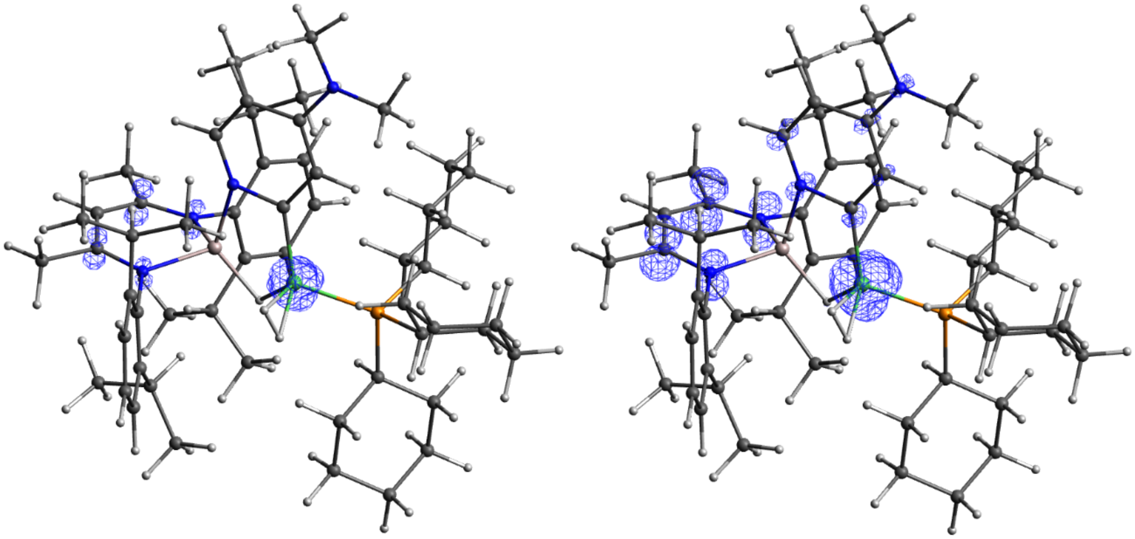

Figure S60 FOD analysis for **Int-5**: left: isovalue = 0.005, right: isovalue = 0.002. N^{FOD} = 0.731.

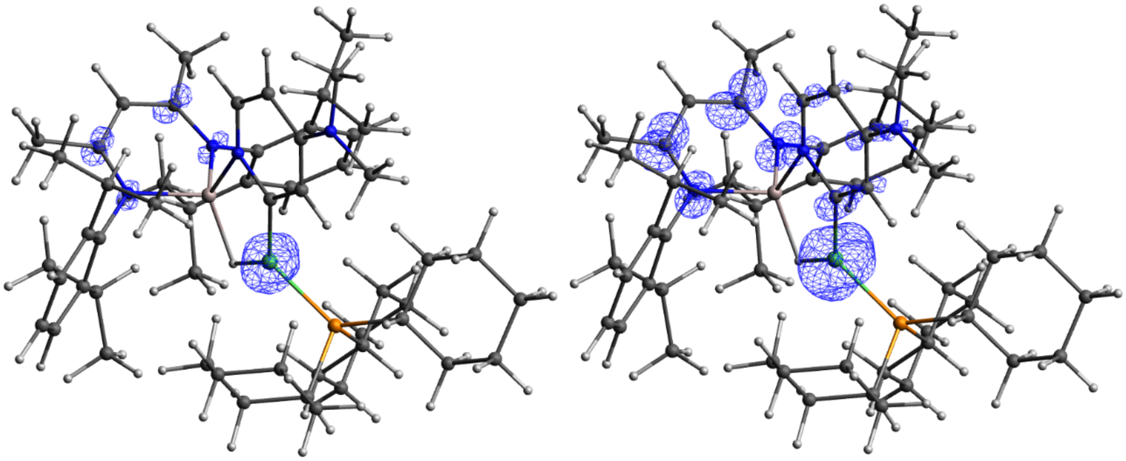

Figure S61 FOD analysis for **Int-6**: left: isovalue = 0.005, right: isovalue = 0.002. N^{FOD} = 0.879.

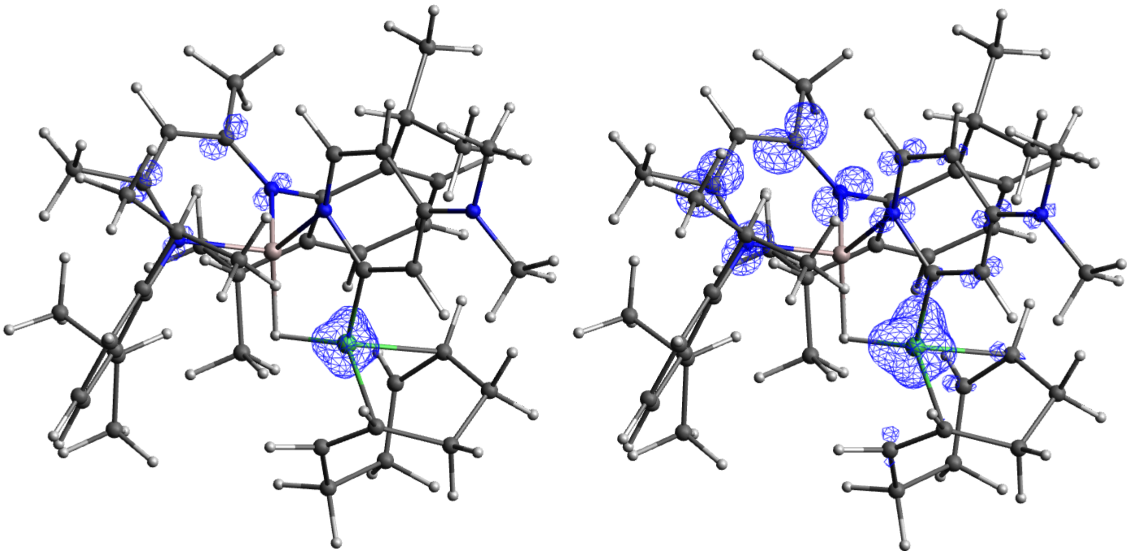

Figure S62 FOD analysis for **3**: left: isovalue = 0.005, right: isovalue = 0.002. N^{FOD} = 0.707.

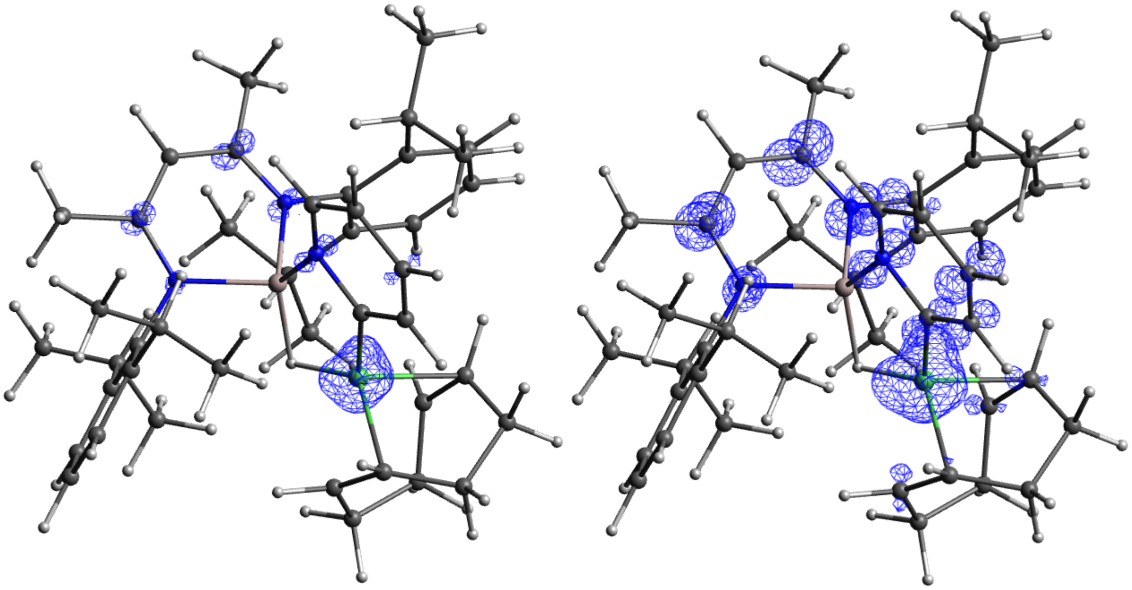

Figure S63 FOD analysis for **4**: left: isovalue = 0.005, right: isovalue = 0.002. N^{FOD} = 0.740.

S7.4.3 Population analyses

Species	**1**	[Ni(COD)$_2$]	DMAP	pyridine	**2**
NPA charge (MBIS charge)					
Ni		+0.57 (+0.48)			+0.32 (-0.06)
Al	+1.36 (+1.38)				+1.42 (+1.25)
H^A	-0.41 (-0.51)				-0.36 (-0.39)
H^B	-0.42 (-0.52)				-0.35 (-0.39)
H^C			+0.20 (+0.09)	+0.20 (+0.10)	
C^A			+0.03 (+0.21)	+0.02 (+0.18)	
N^A			-0.50 (-0.54)	-0.45 (-0.48)	
WBI (MBI)					
Ni---Al					0.26 (0.90)
Ni---H^A					0.14 (0.36)
Ni---H^B					0.14 (0.38)
Ni---H^C					
Al---H^A	0.79 (0.97)				0.75 (0.59)
Al---H^B	0.78 (0.76)				0.75 (0.58)
Al---H^C					
Ni---C^A					
Al---C^N					
Al---N^A					
C^A---N^A			1.40 (1.47)	1.43 (1.48)	
C^A---H^C			0.92 (0.96)	0.92 (0.95)	

Table S11 Numerical data from population analyses.

Species	**2·DMAP**	**Int-3**	**Int-3′**	**TS-1**	**TS-1a**
NPA charge (MBIS charge)					
Ni	+0.33 (-0.14)	-0.11 (-0.63)	+0.13 (-0.65)	+0.17 (-0.66)	+0.36 (-0.17)
Al	+1.45 (+1.17)	+1.45 (+1.27)	+1.52 (+1.45)	+1.59 (+1.46)	+1.55 (+1.42)
H^A	-0.34 (-0.37)	-0.43 (-0.34)	-0.38 (-0.30)	-0.34 (-0.26)	-0.35 (-0.34)
H^B	-0.35 (-0.37)	-0.39 (-0.38)	-0.43 (-0.37)	-0.42 (-0.35)	-0.35 (-0.35)
H^C	+0.25 (+0.13)	+0.25 (+0.15)	+0.17 (+0.14)	+0.07 (+0.05)	+0.08 (+0.01)
C^A	+0.08 (+0.20)	+0.08 (+0.19)	-0.03 (+0.16)	-0.02 (+0.17)	-0.01 (+0.10)
N^A	-0.63 (-0.47)	-0.65 (-0.48)	-0.73 (-0.55)	-0.76 (-0.55)	-0.75 (-0.54)
WBI (MBI)					
Ni---Al	0.27 (0.55)	0.28 (0.17)	0.15 (0.42)	0.13 (0.35)	0.13 (0.45)
Ni---H^A	0.18 (0.71)	0.16 (0.97)	0.17 (0.74)	0.19 (0.71)	0.17 (0.53)
Ni---H^B	0.18 (0.56)	0.23 (0.99)	0.13 (0.68)	0.14 (0.67)	0.16 (0.67)
Ni---H^C	0.00 (<0.10)	0.01 (<0.10)	0.09 (0.46)	0.23 (0.71)	0.24 (0.55)
Al---H^A	0.44 (0.21)	0.45 (<0.10)	0.39 (0.26)	0.35 (0.22)	0.42 (0.39)
Al---H^B	0.46 (0.25)	0.44 (0.17)	0.52 (0.44)	0.50 (0.45)	0.45 (0.41)
Al---H^C	0.01 (<0.10)	0.01 (<0.10)	0.02 (<0.10)	0.03 (<0.10)	0.08 (<0.10)
Ni---C^A	0.00 (<0.10)	0.01 (<0.10)	0.27 (0.66)	0.31 (0.81)	0.31 (0.80)
Al---C^N	0.01 (<0.10)	0.02 (<0.10)	0.06 (<0.10)	0.09 (<0.10)	0.08 (<0.10)
Al---N^A	0.25 (0.49)	0.24 (0.37)	0.26 (0.49)	0.25 (0.51)	0.27 (0.51)
C^A---N^A	1.30 (1.30)	1.28 (1.30)	1.15 (1.19)	1.16 (1.28)	1.17 (1.28)
C^A---H^C	0.89 (0.91)	0.88 (0.92)	0.76 (0.61)	0.54 (0.38)	0.54 (0.39)

Table S11 continued. Numerical data from population analyses.

Species	**Int-4**	**TS-2**	**Int-5**	**TS-3**	**Int-6**
NPA charge (MBIS charge)					
Ni	+0.19 (-0.54)	+0.09 (-0.83)	+0.09 (-0.84)	+0.11 (-0.62)	+0.10 (-0.78)
Al	+1.61 (+1.43)	+1.66 (+1.39)	+1.70 (+1.38)	+1.68 (+1.42)	+1.66 (+1.40)
H^A	-0.38 (-0.33)	-0.26 (-0.17)	-0.33 (-0.20)	-0.44 (-0.36)	-0.42 (-0.34)
H^B	-0.24 (-0.20)	-0.23 (-0.35)	-0.19 (-0.11)	-0.12 (-0.09)	-0.02 (+0.04)
H^C	-0.18 (-0.13)	-0.14 (-0.18)	-0.14 (-0.10	-0.13 (-0.06)	-0.03 (+0.07)
C^A	-0.01 (+0.07)	-0.02 (+0.12)	-0.03 (+0.12)	-0.07 (+0.02)	-0.13 (-0.02)
N^A	-0.80 (-0.54)	-0.81 (-0.53)	-0.81 (-0.54)	-0.80 (-0.54)	-0.80 (-0.53)
WBI (MBI)					
Ni---Al	0.14 (0.38)	0.21 (0.55)	0.22 (0.58)	0.14 (0.40)	0.21 (0.50)
Ni---H^A	0.15 (0.66)	0.24 (0.69)	0.21 (0.60)	0.09 (0.52)	0.13 (0.60)
Ni---H^B	0.26 (0.81)	0.25 (0.63)	0.41 (0.71)	0.32 (0.55)	0.15 (0.24)
Ni---H^C	0.40 (0.85)	0.39 (0.73)	0.41 (0.67)	0.36 (0.69)	0.16 (0.22)
Al---H^A	0.51 (0.33)	0.29 (0.28)	0.36 (0.34)	0.57 (0.44)	0.54 (0.37)
Al---H^B	0.24 (0.13)	0.24 (<0.10)	0.12 (0.12)	0.00 (0.23)	0.04 (<0.10)
Al---H^C	0.05 (<0.10)	0.07 (<0.10)	0.07 (<0.10)	0.00 (<0.10)	0.02 (<0.10)
Ni---C^A	0.34 (0.80)	0.29 (0.63)	0.29 (0.78)	0.31 (0.72)	0.33 (0.72)
Al---C^N	0.14 (0.13)	0.14 (0.16)	0.13 (0.11)	0.14 (<0.10)	0.20 (<0.10)
Al---N^A	0.25 (0.52)	0.23 (0.53)	0.23 (0.57)	0.24 (0.61)	0.24 (0.68)
C^A---N^A	1.20 (1.26)	1.19 (1.31)	0.19 (1.23)	1.19 (1.19)	1.20 (1.15)
C^A---H^C	0.11 (<0.10)	0.18 (<0.10)	0.10 (<0.10)	0.06 (<0.10)	0.04 (<0.10)

Table S11 continued. Numerical data from population analyses.

Species	**Int-7**	**3**	**4**
NPA charge (MBIS charge)			
Ni	+0.06 (-0.59)	+0.38 (-0.18)	+0.39 (-0.19)
Al	+1.65 (+1.33)	+1.70 (+1.34)	+1.72 (+1.33)
H^A	-0.41 (-0.36)	-0.36 (-0.37)	-0.36 (-0.36)
H^B			
H^C			
C^A	-0.14 (-0.07)	-0.10 (-0.16)	-0.11 (-0.16)
N^A	-0.79 (-0.50)	-0.79 (-0.51)	-0.76 (-0.46)
WBI (MBI)			
Ni---Al	0.27 (0.41)	0.20 (0.70)	0.20 (0.73)
Ni---H^A	0.18 (0.66)	0.16 (0.41)	0.16 (0.49)
Ni---H^B			
Ni---H^C			
Al---H^A	0.50 (0.38)	0.50 (0.50)	0.49 (0.47)
Al---H^B			
Al---H^C			
Ni---C^A	0.38 (0.61)	0.33 (0.48)	0.35 (0.65)
Al---C^N	0.20 (0.21)	0.20 (<0.10)	0.20 (<0.10)
Al---N^A	0.24 (0.69)	0.25 (0.64)	0.24 (0.62)
C^A---N^A	1.20 (1.25)	1.20 (1.21)	1.24 (1.20)
C^A---H^C			

Table S11 continued. Numerical data from population analyses.

Species	**2**	**2·DMAP**
Löwdin		
Ni	[Ar] 3d(9.30) 4s(0.20) 4p(1.30) 4f(0.38)	[Ar] 3d(9.21) 4s(0.21) 4p(1.15) 4f(0.38)
Al	[Ne] 3s(0.38) 3p(1.28) 3d(1.07) 4f(0.56) 5g(0.15)	[Ne] 3s(0.36) 3p(1.27) 3d(1.27) 4f)0.71) 5g(0.16)
H^A	1s(0.80) 2p(0.12) 3d(0.03)	1s0(.79) 2p(0.13) 3d(0.04)
H^B	1s(0.80) 2p(0.12) 3d(0.03)	1s(0.78) 2p(0.14) 3d(0.05)
Mulliken		
Ni	[Ar] 3d(8.80) 4s(0.43) 4p(0.42) 4f(0.03)	[Ar] 3d(8.60) 4s(0.80) 4p(0.39) 4f(0.03)
Al	[Ne] 3s(1.07) 3p(1.34) 3d(0.43) 4f(0.06)	[Ne] 3s(0.79) 3p(1.21) 3d(0.52) 4f(0.07)
H^A	1s(1.04) 2p(0.04)	1s(1.15) 2p(0.04)
H^B	1s(0.97) 2p(0.04)	1s(1.32) 2p(0.04)
NPA		
Ni	[Ar] 4s(0.28) 3d(9.35)	[Ar] 4s(0.30) 3d(9.32)
Al	[Ne] 3s(0.62) 3p(0.92)	[Ne] 3s(0.58) 3(p0.91)
H^A	1s(1.34)	1s(1.35)
H^B	1s(1.34)	1s(1.75)

Table S12 Electron configurations based on Löwdin, Mulliken, and Natural population analyses.

S7.4.4 QTAIM analysis

Species	**2**	**2·DMAP**
**ϱ(r)**		
Ni---H^A	0.089	0.099
Ni---H^B	0.089	0.105
Al---H^A	0.068	0.060
Al---H^B	0.068	0.058
**∇^2ϱ(r)**		
Ni---H^A	0.223	0.227
Ni---H^B	0.222	0.236
Al---H^A	0.172	0.125
Al---H^B	0.172	0.112
**ellipticity**		
Ni---H^A	0.467	0.254
Ni---H^B	0.471	0.237
Al---H^A	0.383	1.383
Al---H^B	0.383	2.211
**sign(λ)*ϱ(r) [DFT density]**		
Ni---H^A	-0.089	-0.099
Ni---H^B	-0.089	-0.105
Al---H^A	-0.068	-0.060
Al---H^B	-0.068	-0.058
**sign(λ)*ϱ(r) [promolecular density]**		
Ni---H^A	-0.073	-0.080
Ni---H^B	-0.073	-0.084
Al---H^A	-0.097	-0.086
Al---H^B	-0.097	-0.083

Table S13 Numerical QTAIM for the M---H BCPs in **2** and **2·DMAP**.

S7.4.5 ETS-NOCV analysis

ETS-NOCV analysis was employed to elucidate the bonding within the bimetallic core in **2** and **2·DMAP**.

Deformation density plots were generated using an isovalue = 0.01. Plots of the individual NOCVs used an isovalue = 0.1. Due to the limitations of the implementation in ORCA 5.0.4, only closed shell fragmentations were considered. Orbital contributions were obtained from a Löwdin population analysis.

S7.4.5.1 2

S7.4.5.1.1 Fragmentation

Two fragmentations were considered. In fragmentation a, we consider the binding of **1** to Ni^{0}(COD), whereas fragmentation b is comprised of (^{Dipp}BDI)Al(I) and H_2Ni^{II}(COD). Based on the stark difference in ΔE_{orb} (Figure S64) only fragmentation a is discussed in detail. The symmetry of individual interactions is given with respect to the approximate C_{2v} point group.

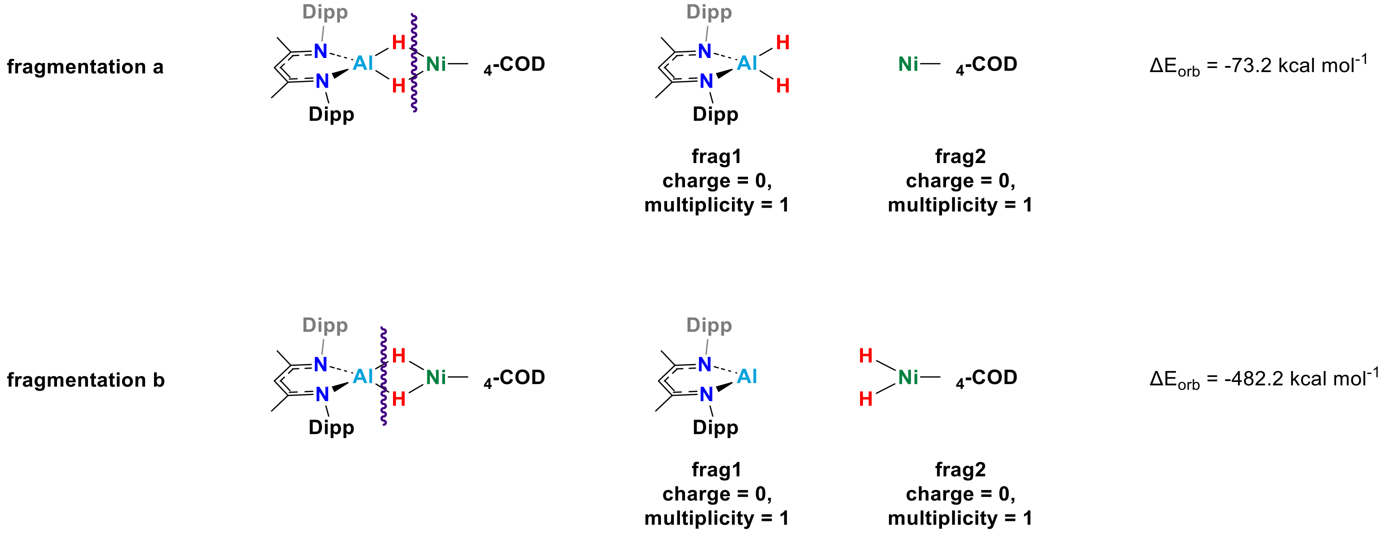

Figure S64 Fragmentation patters for the ETS-NOCV analysis of **2**.

S7.4.5.1.2 Data from fragmentation a

**Energy, symmetry, composition**	**Deformation density**	**Donor NOCV**	**Acceptor NOCV**
**$\Delta\rho_1$** = -17.7 kcal mol^{-1} (24.1 %)<br>**Symmetry:** B_2<br>**Donor**:<br>**frag1:** 22.3 % Al (16.9 % p_z), 14.4 % H_A (10.3 % s), 14.8 % H_B (10.3 % s)<br>**frag2:** 21.3 % Ni (16.0 % d_{yz})<br>**Acceptor**:<br>**frag1:** 9.5 % H_A (8.2 % s), 9.5 % H_B (8.2 % s), 12.8 % Al (7.1 % d_{yz})<br>**frag2:** 31.0 % Ni (17.6 % p_z, 12.4 % d_{yz})	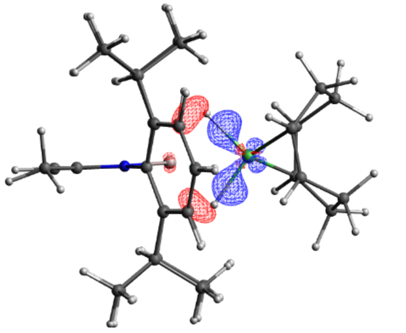<br>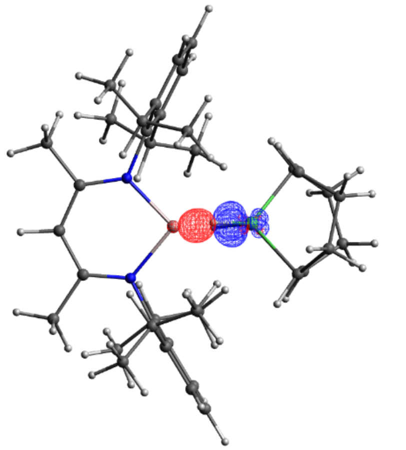	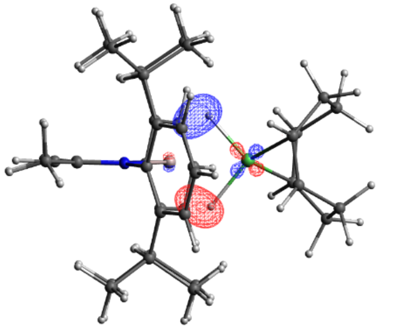<br>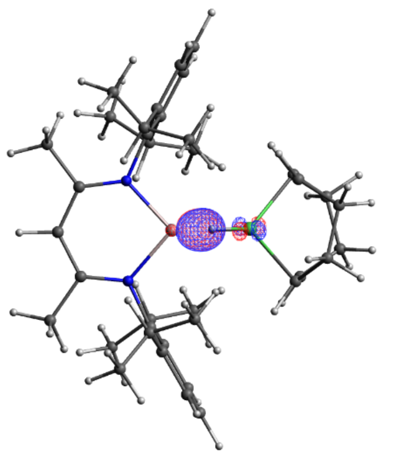	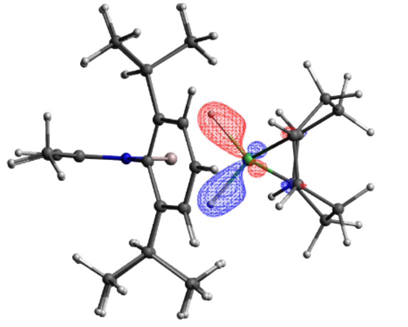<br>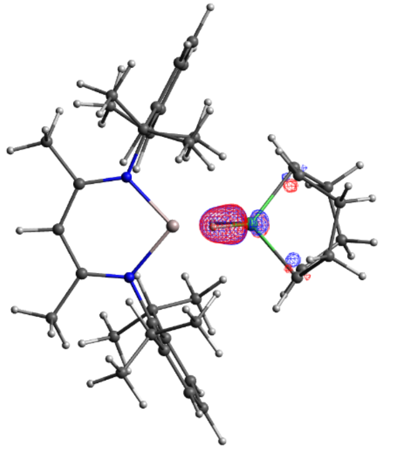

Table S14 ETS-NOCV analysis for **2** using fragmentation a. ΔE_{orb} = -73.2 kcal mol^{-1}.

**$\Delta\rho_2$** = -15.9 kcal mol^{-1} (21.7 %)<br>**Symmetry:** B_1<br>**Donor**:<br>**frag1:** 12.7 % Al (6.1 % p_x, 4.5 % d_{xy})<br>**frag2:** 59.2 Ni (56.3 % d_{xy})<br>**Acceptor**:<br>**frag1:** 5.1 % H_A, 5.0 % H_B (8.2 % s), 36.9 Al (16.8 % p_x, 19.4 % d_{xy})<br>**frag2:** 31.8 % Ni (21.0 % d_{xy}, 7.0 % p_x)	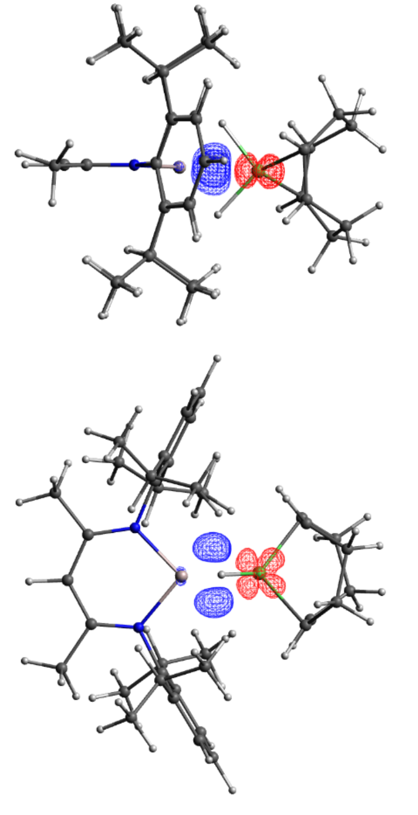	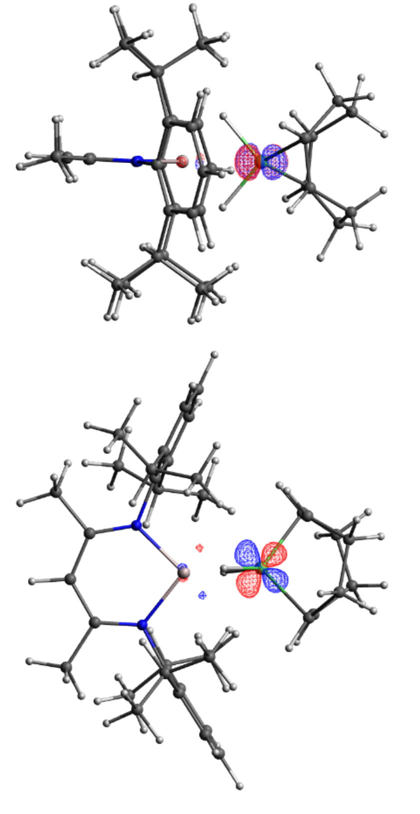	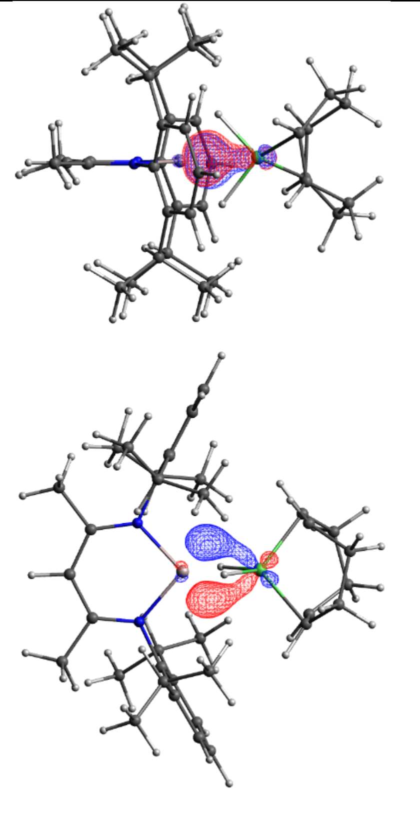

Table S14 continued. ETS-NOCV analysis for **2** using fragmentation a. ΔE_{orb} = -73.2 kcal mol^{-1}.

**$\Delta\rho_3$** = -16.5 kcal mol^{-1} (22.5 %)<br>**Symmetry:** A_1<br>**Donor**:<br>**frag1:** 13.2 % H_A (10.0 % s), 13.8 % H_A (10.5 % s), 11.3 % Al (5.3 % d_{z2})<br>**frag2:** 53.2 % Ni (43.5 % d_{z2})<br>**Acceptor**:<br>**frag1:** 8.9 % H_A, 8.8 % H_A, 22.4 % Al (6.0 % s, 7.3 % p_y)<br>**frag2:** 42.9 % Ni (26.8 % d_{z2})	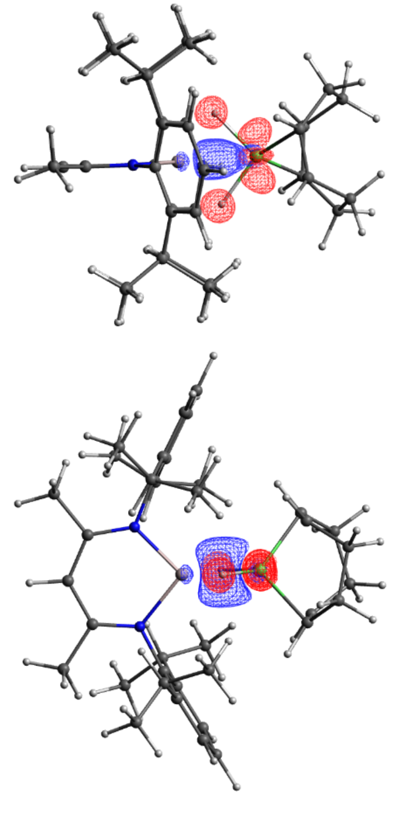	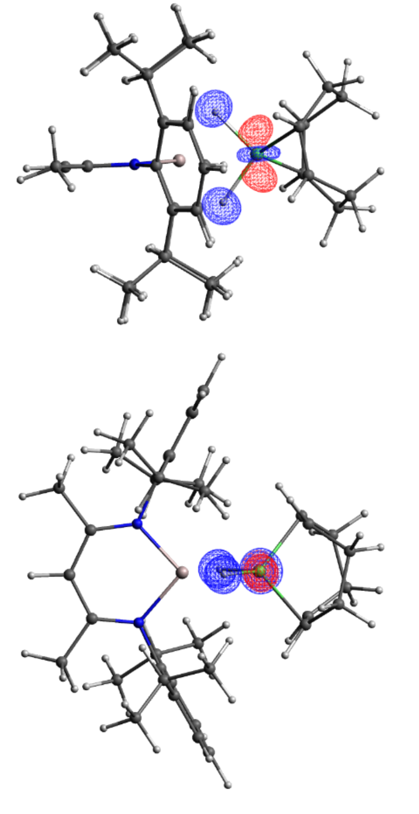	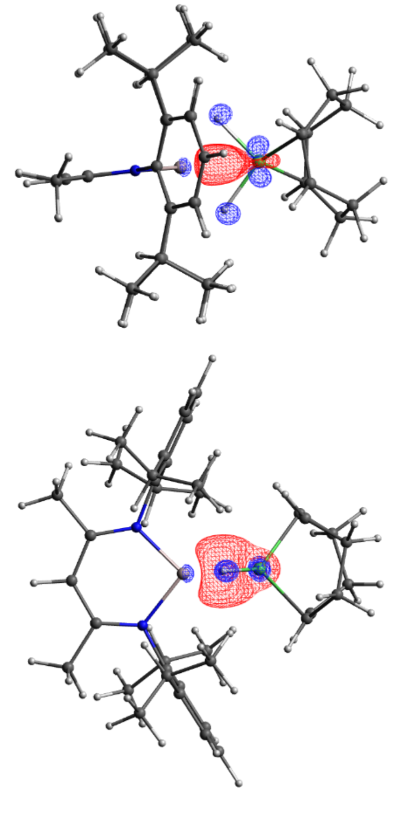

Table S14 continued. ETS-NOCV analysis for **2** using fragmentation a. ΔE_{orb} = -73.2 kcal mol^{-1}.

**$\Delta\rho_4$** = -8.6 kcal mol^{-1} (11.7 %)<br>**Symmetry:** A_1<br>**Donor**:<br>**frag1:** 5.4 % H_A (4.4 % s), 5.3 % H_A (4.3 % s), 18.5 % Al (6.1 % s, 5.9 % d_{z2})<br>**frag2:** 34.6 % Ni (25.1 % d_{x2-y2})<br>**Acceptor**:<br>**frag1:** 13.9 % H_A (10.3 % s), 13.7 % H_A (10.1 % s), 23.8 % Al (6.9 % p_y, 8.4 % d_{x2-y2})<br>**frag2:** 35.7 % Ni (23.9 % d_{x2-y2})	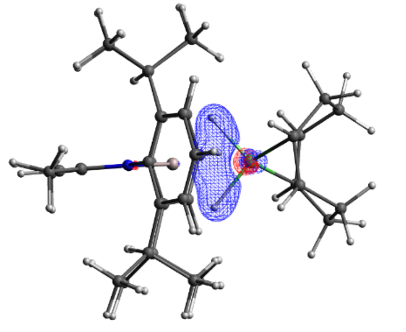<br>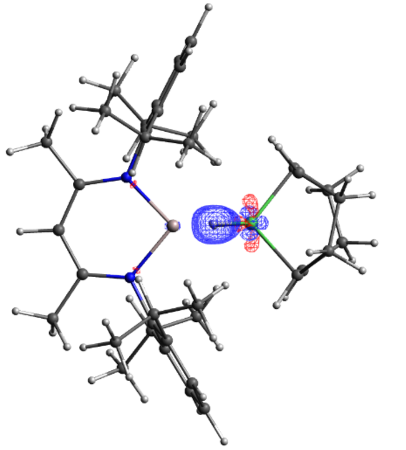	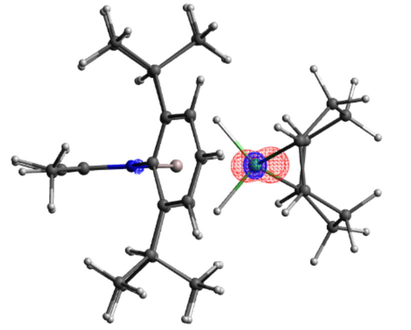<br>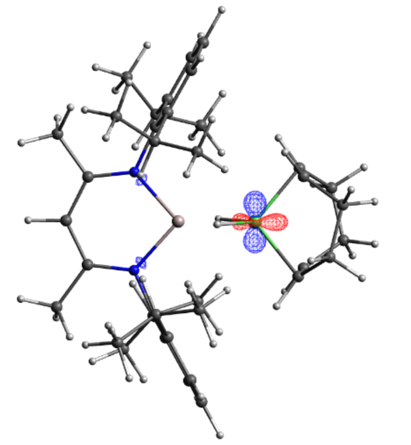	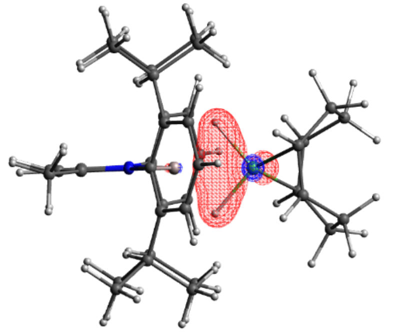<br>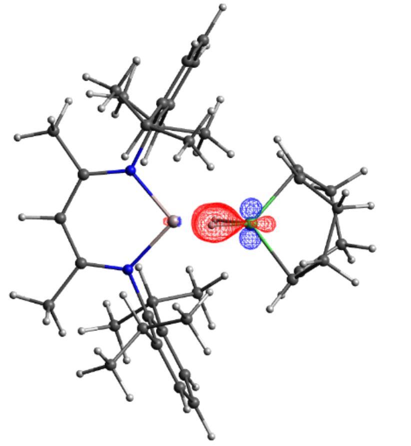

Table S14 continued. ETS-NOCV analysis for **2** using fragmentation a. ΔE_{orb} = -73.2 kcal mol^{-1}.

Δ_{Q5} = -6.1 kcal mol^{-1} (8.3 %)<br>**Symmetry:** B_2<br>**Donor**:<br>**frag1:** 7.4 % H_A (6.0 % s), 6.3 % H_A (5.4 % s), 7.2 % Al<br>**frag2:** 55.4 % Ni (50.8 % d_{yz})<br>**Acceptor**:<br>**frag1:** 8.9 % H_A (6.7 % s), 9.6 % H_A (7.5 % s), 12.3 % Al (5.5 % d_{yz})<br>**frag2:** 32.8 % Ni (25.4 % d_{yz})	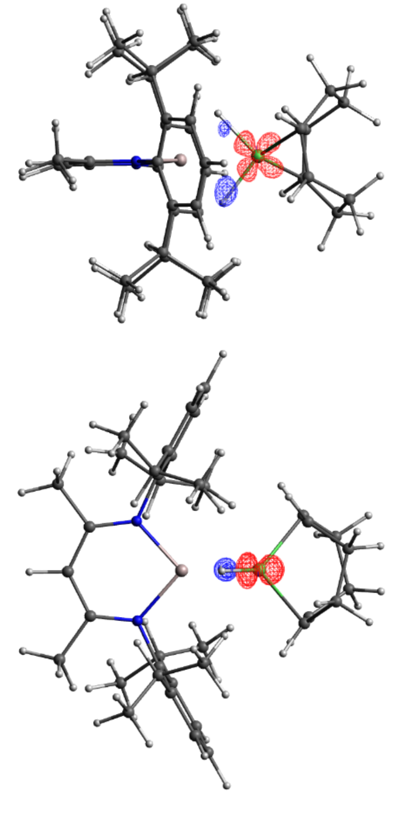	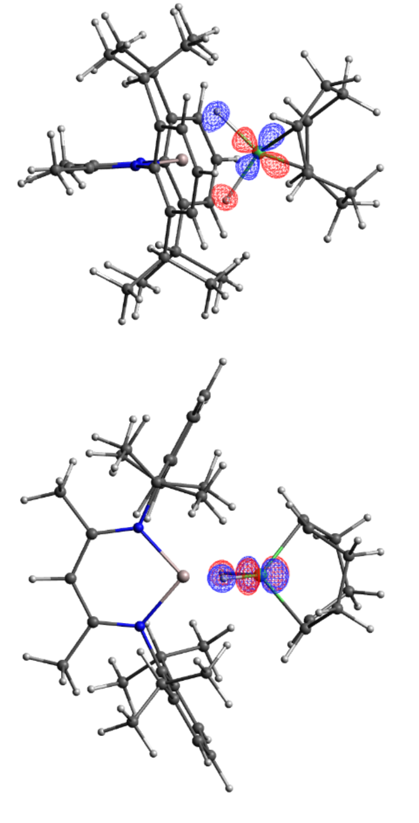	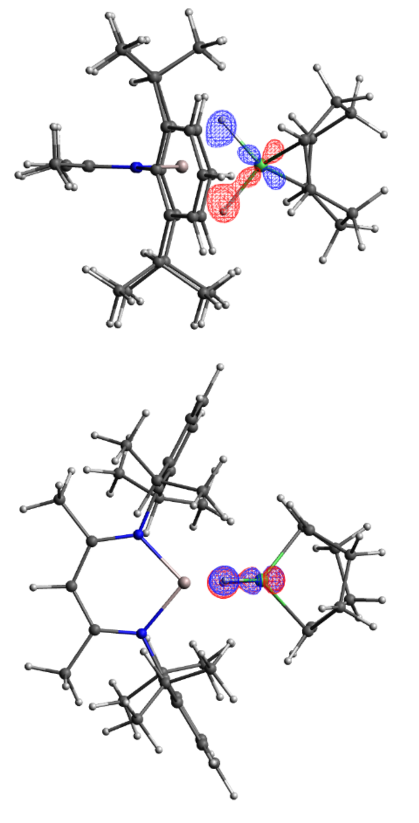

Table S14 continued. ETS-NOCV analysis for 2 using fragmentation a. ΔE_{orb} = -73.2 kcal mol-1.

Δ_{Q6} = -6.1 kcal mol^{-1} (8.3 %)<br>**Symmetry:** A_1<br>**Donor**:<br>**frag1:** 6.9 % H_A, 7.7 % H_A, 22.6 % Al (9.5 % s, 7.7 % p_y)<br>**frag2:** 49.7 % Ni (25.8 % d_{yx2-y2}, 14.3 % d_{z2})<br>**Acceptor**:<br>**frag1:** 10.0 % H_A (6.0 % s), 10.1 % H_A (5.5 % s), 9.6 % Al<br>**frag2:** 43.0 % Ni (10.8 % d_{x2-y2}, 7.7 % s, 6.5 % d_{z2}, 6.4 % p_y 5.0 % d_{xy})	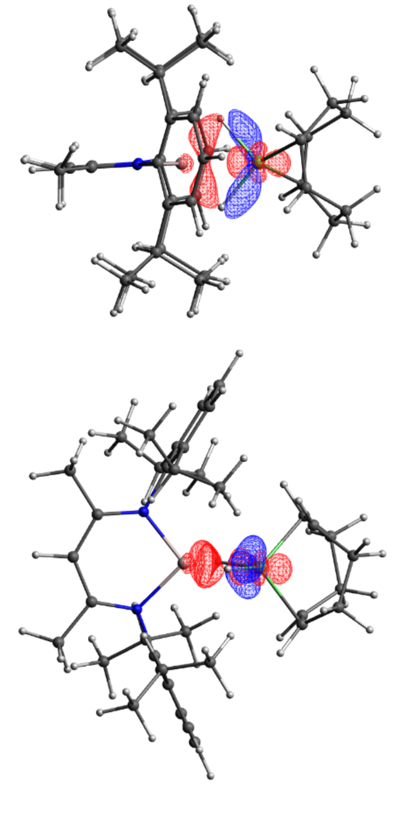	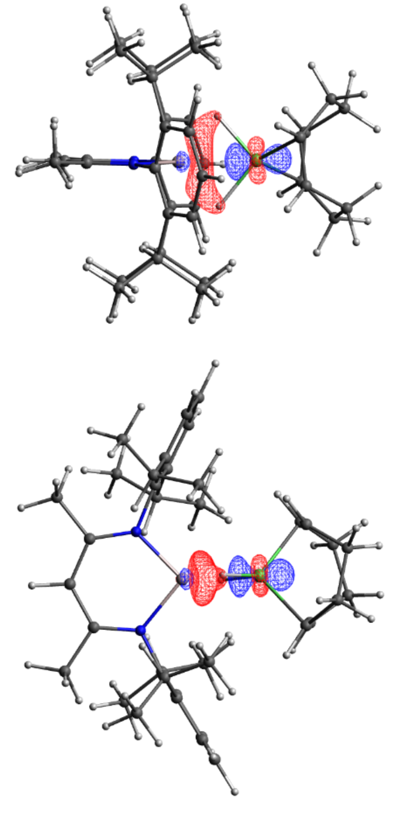	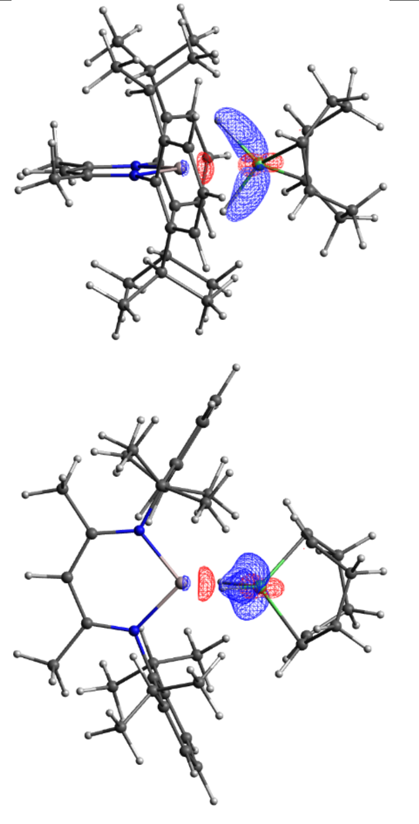

Table S14 continued. ETS-NOCV analysis for **2** using fragmentation a. ΔE_{orb} = -73.2 kcal mol^{-1}.

*S7.4.5.2 **2·DMAP***

S7.4.5.2.1 Fragmentation

Two fragmentations were considered. In fragmentation a, we consider the binding of **1·DMAP** to Ni^0(COD), whereas fragmentation b is comprised of (^{Dipp}BDI)Al(I) DMAP and H_2Ni^{II}(COD). Based on the stark difference in ΔE_{orb} (Figure S65) only fragmentation a is discussed in detail. The symmetry of the individual interactions is given with respect to the approximate C_s symmetry.

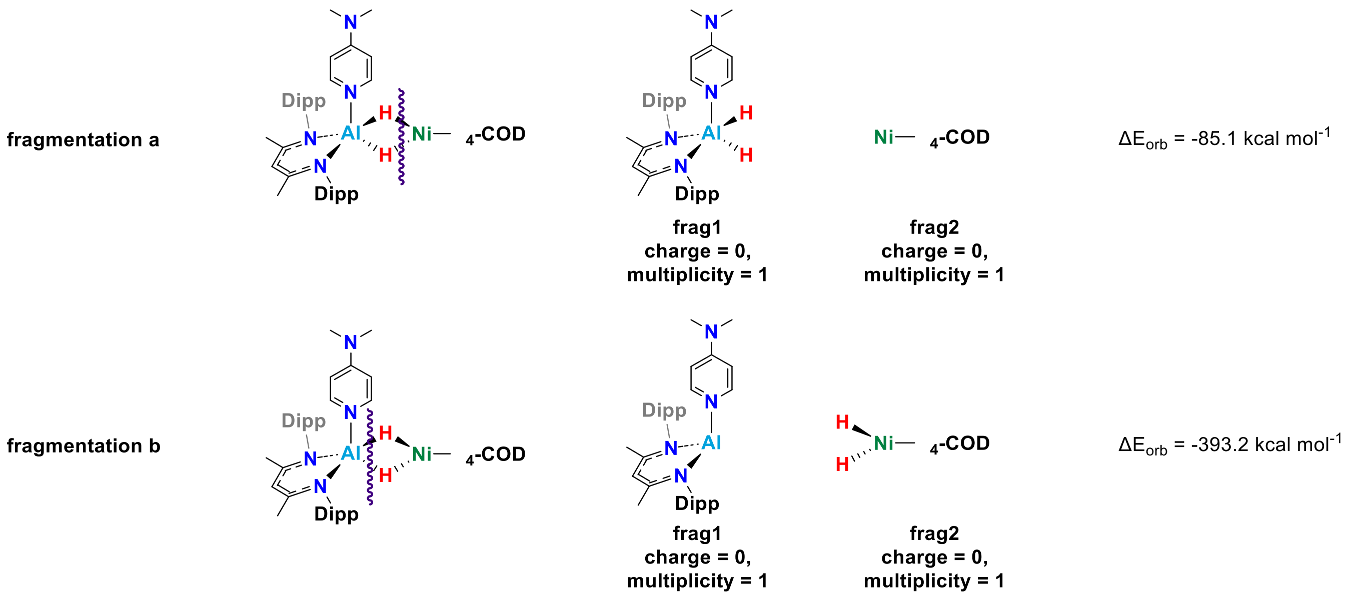

Figure S65 Fragmentation patters for the ETS-NOCV analysis of **2·DMAP**.

S7.4.5.2.2 Data from fragmentation a

**Energy, symmetry, composition**	**Deformation density**	**Donor NOCV**	**Acceptor NOCV**
**$\Delta\varrho_1$** = -25.2 kcal mol^{-1} (29.6 %)<br>**Symmetry:** *A''*<br>**Donor**:<br>**frag1:** 18.8 % Al (5.6 % p_x, 4.7 % d_{x2-y2}, 4.4 % d_{xy}), 14.8 % H_A (11.1 % s),12.5 % H_B (9.6 % s)<br>**frag2:** 25.3 % Ni (9.7 % d_{xz})<br>**Acceptor**:<br>**frag1:** 10.0 % Al, 7.5 % H_A (6.0 % s), 8.8 % H_B (7.8 % s)<br>**frag2:** 28.3 % Ni (14.1 % px)	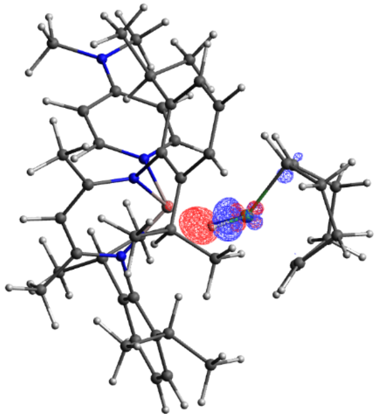<br>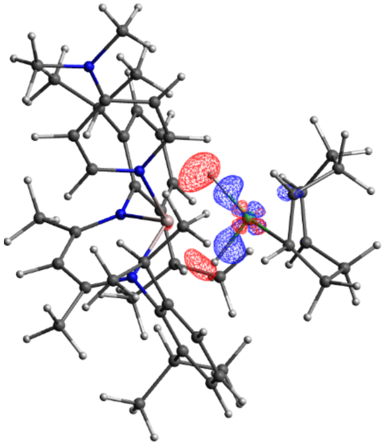	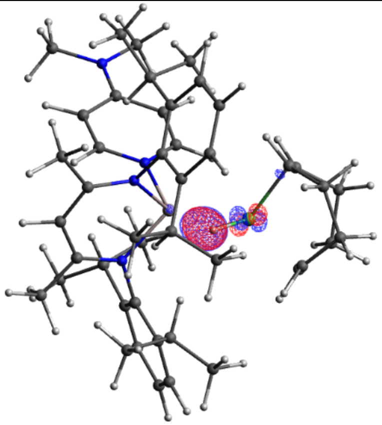<br>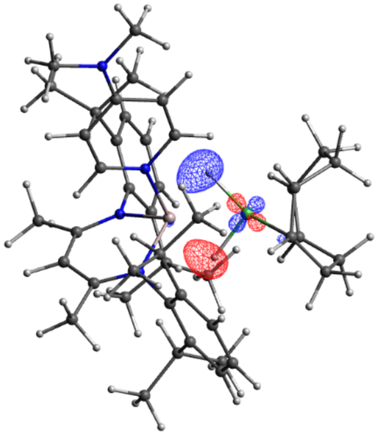	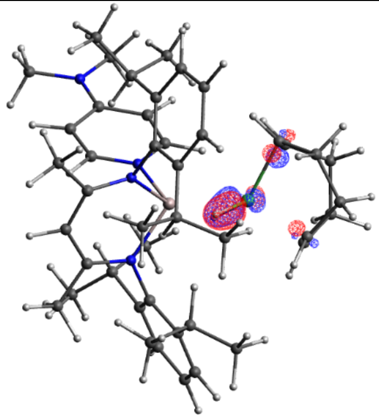<br>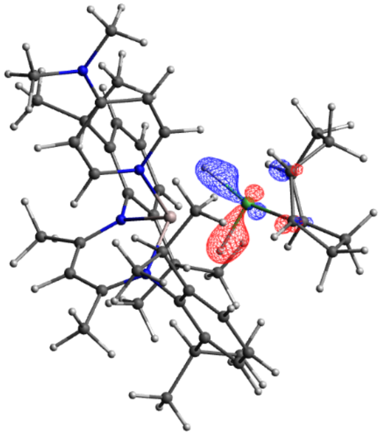

Table S15 ETS-NOCV analysis for **2·DMAP** using fragmentation a. ΔE_{orb} = -85.1 kcal mol^{-1}.

<table>
<tr>
<td rowspan="2">$\Delta\rho_2$ = -25.4 kcal mol^{-1} (29.8 %)
Symmetry: A′
Donor:
frag1: 8.4 % Al, 1.9 % H_A, 6.2 % H_B (4.4 % s)
frag2: 60.8 % Ni (30.3 % d_{xy}, 13.3 % $3d_{z2}$, 12.8 % d_{x2-y2})
Acceptor:
frag1: 6.4 % H_A (3.1 % s), 4.4 % H_B (2.7 % p_x), 31.4 % Al (12.9 % p_z, 10.6 % d_{z2})
frag2: 34.0 % Ni (10.1 % d_{xy}, 7.1 % $4p_y$)</td>
<td>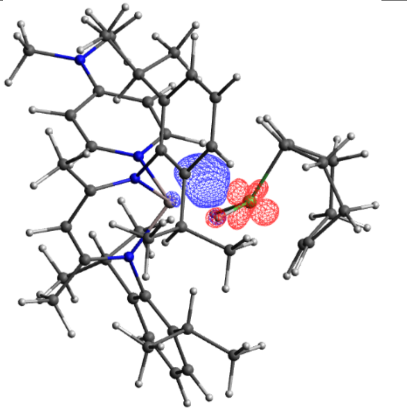</td>
<td>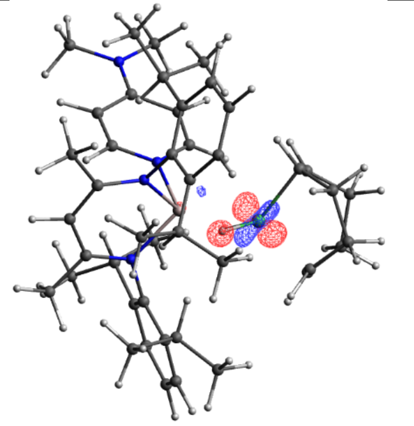</td>
<td>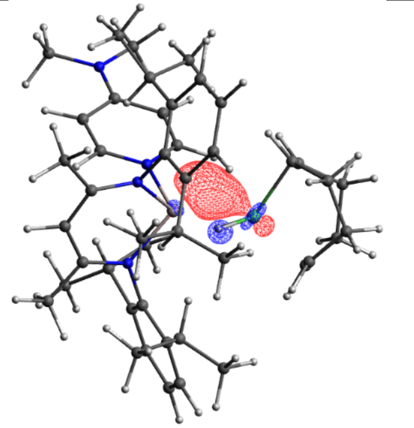</td>
</tr>
<tr>
<td>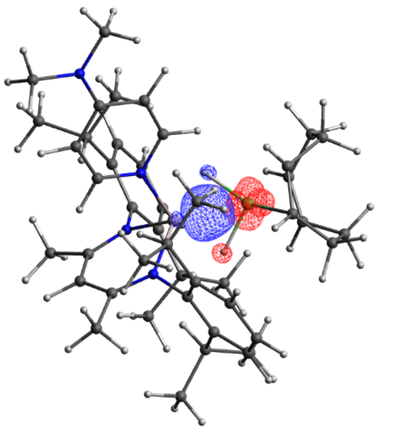</td>
<td>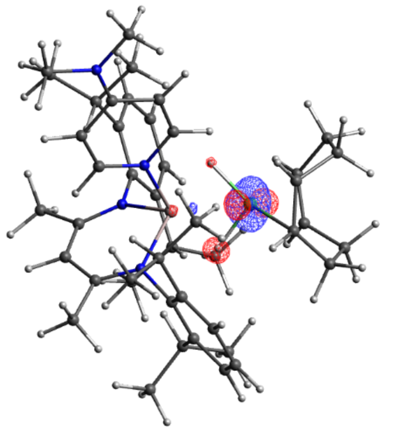</td>
<td>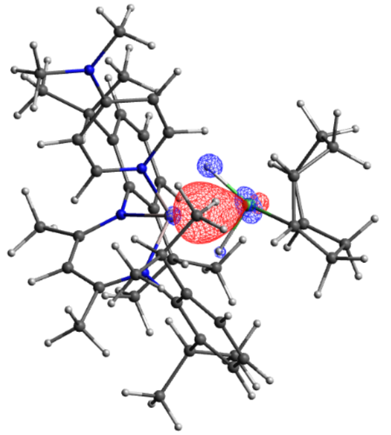</td>
</tr>
</table>

Table S15 ETS-NOCV analysis for **2·DMAP** using fragmentation a. ΔE_{orb} = -85.1 kcal mol-1.

**Δϱ₃** = -11.2 kcal mol^{-1} (13.2 %)<br>**Symmetry:** *A′*<br>**Donor**:<br>**frag1:** 13.8 % H_A (9.2 % s), 11.6 % H_A (8.1 % s), 16.1 % Al<br>**frag2:** 33.6 % Ni (17.2 % d_{z2})<br>**Acceptor**:<br>**frag1:** 9.3 % H_A (5.8 % p_x), 7.7 % H_A, 21.1 % Al (6.9 % p_y)<br>**frag2:** 34.9 % Ni (10.6 % p_z, 8.0 % d_{z2})	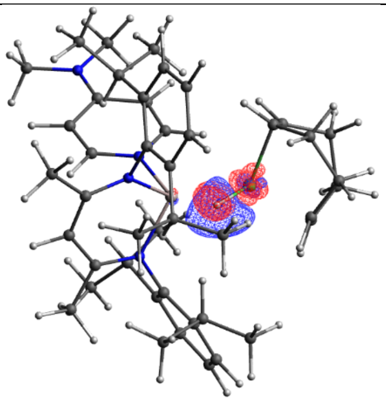<br>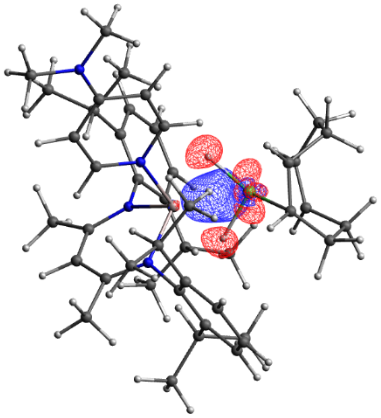	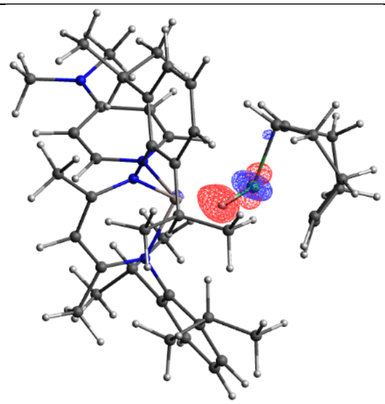<br>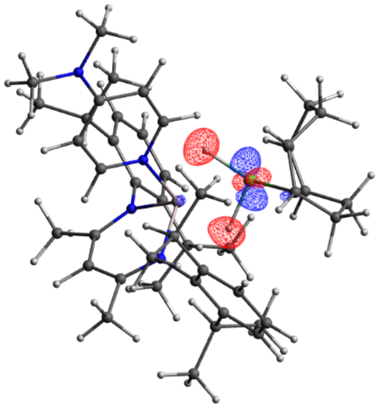	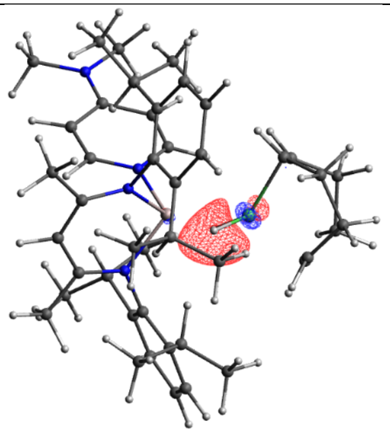<br>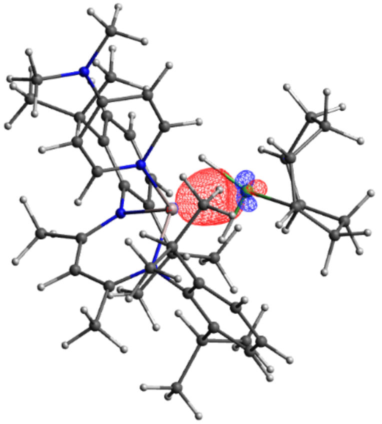

Table S15 ETS-NOCV analysis for **2·DMAP** using fragmentation a. ΔE_{orb} = -85.1 kcal mol^{-1}.

**$\Delta\rho_4$** = -7.3 kcal mol^{-1} (8.6 %)<br>**Symmetry:** *A′*<br>**Donor**:<br>**frag1:** 4.9 % H_A (3.5 % s), 6.2 % H_A (4.9 % s), 18.5 % Al (6.3 % s, 4.3 % p_y)<br>**frag2:** 33.6 % Ni (10.7 % d_{z2}, 8.0 % d_{xy})<br>**Acceptor**:<br>**frag1:** 14.6 % H_A (11.4 % 1), 22.0 % H_A (18.4 % s), 16.6 % Al (5.9 % d_{yz})<br>**frag2:** 33.1% Ni (7.0 % d_{z2}, 4.9 % s)	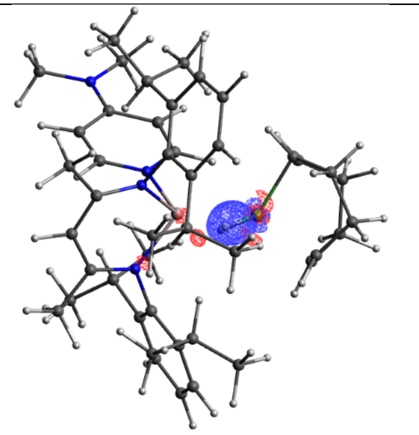<br>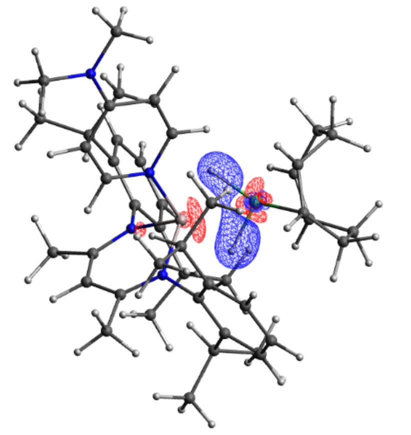	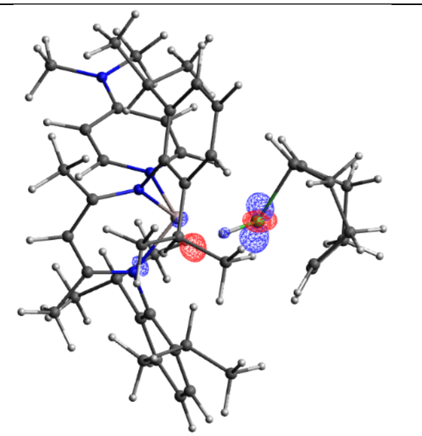<br>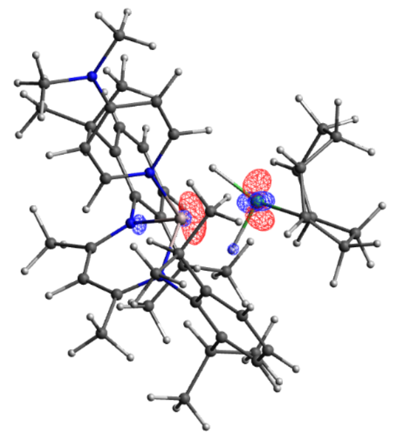	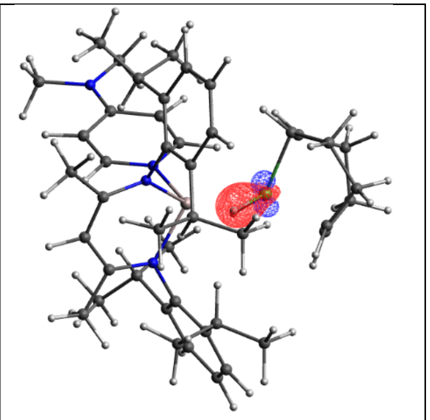<br>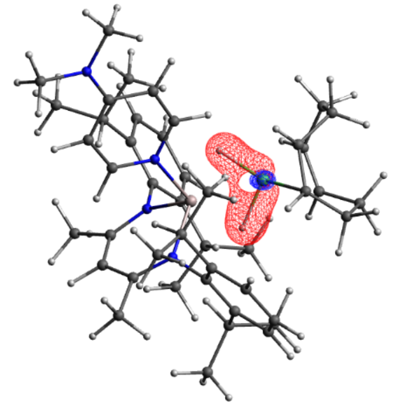

Table S15 ETS-NOCV analysis for **2·DMAP** using fragmentation a. ΔE_{orb} = -85.1 kcal mol^{-1}.

Δ_{Q5} = -6.7 kcal mol^{-1} (7.9 %)<br>**Symmetry:** *B''*<br>**Donor**:<br>**frag1:** 10.4 % H_A (8.4 % s), 1.6 % H_A (1.4 % s), 5.0 % Al<br>**frag2:** 55.2 % Ni (37.3 % d_{yz}, 14.0 % d_{x2-y2})<br>**Acceptor**:<br>**frag1:** 15.6 % H_A (12.2 % s), 5.7 % H_B (4.6 % s), 8.2 % Al<br>**frag2:** 37.9 % Ni (24.5 % d_{xz})	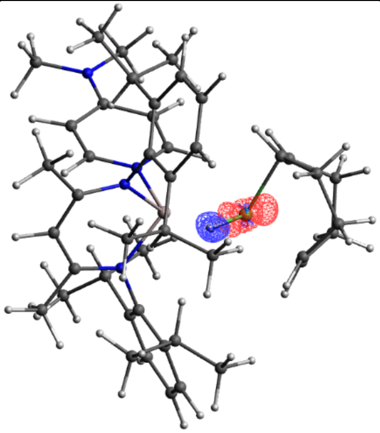<br>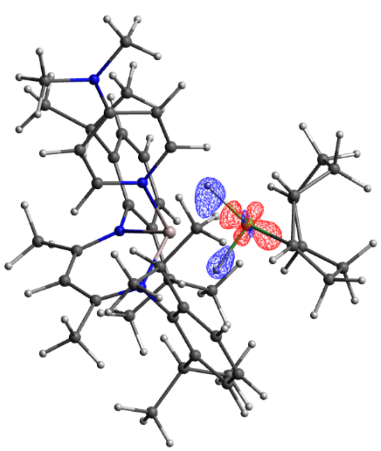	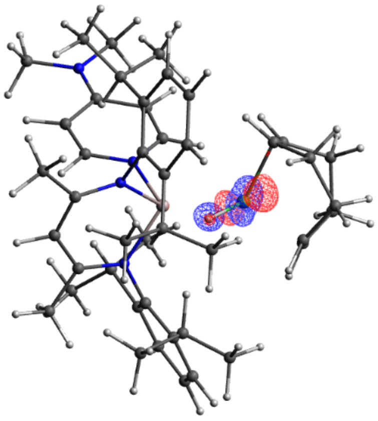<br>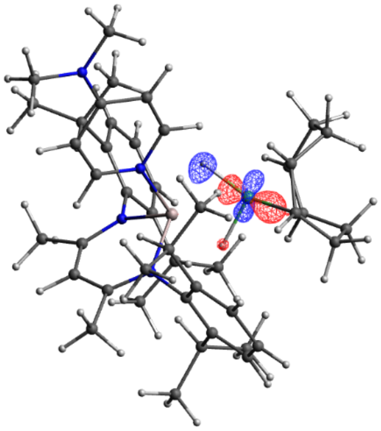	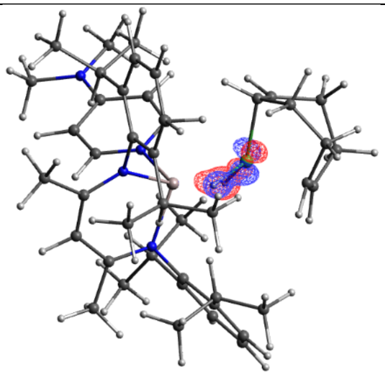<br>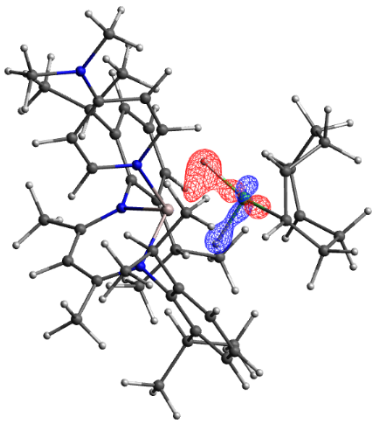

Table S15 ETS-NOCV analysis for **2·DMAP** using fragmentation a. ΔE_{orb} = -85.1 kcal mol^{-1}.

Δ_{Q6} = -6.1 kcal mol^{-1} (7.2 %)<br>**Symmetry:** *A''*<br>**Donor**:<br>**frag1:** 1.3 % H_A, 7.5 % H_A (3.4 % s), 11.6 % Al (3.3 % s, 3.9 % p_y)<br>**frag2:** 61.0 % Ni (37.3 % $3d_{yz}$, 14.0 % d_{z2-y2})<br>**Acceptor**:<br>**frag1:** 5.8 % H_A (2.9 % s), 9.9 % H_A (3.0 % s, 4.0 % p_x), 12.3 % Al (2.9 % s, 3.3 % p_y)<br>**frag2:** 43.3 % Ni (23.5 % d_{xy}, 6.8 % d_{x2-y2})	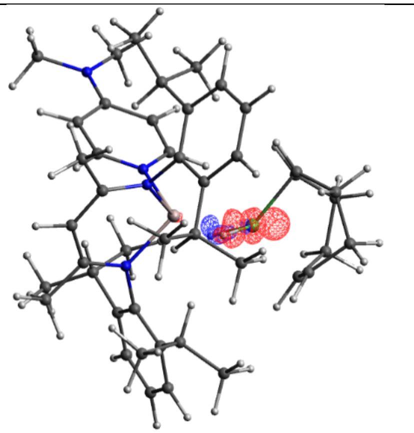<br>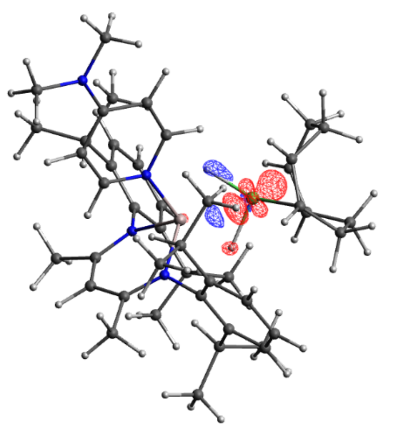	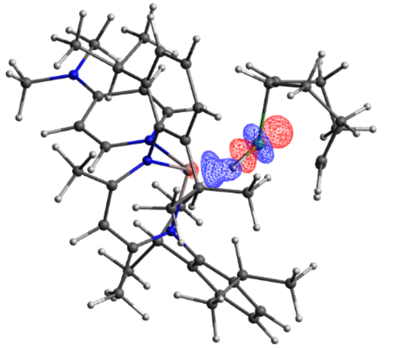<br>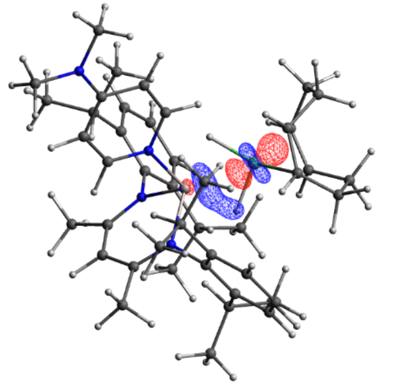	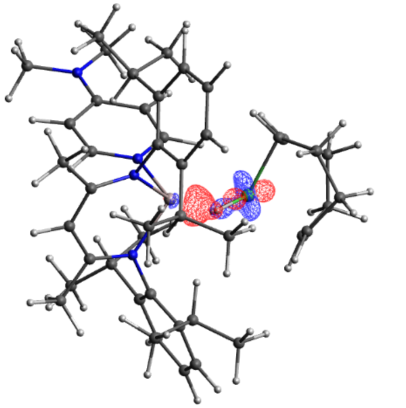<br>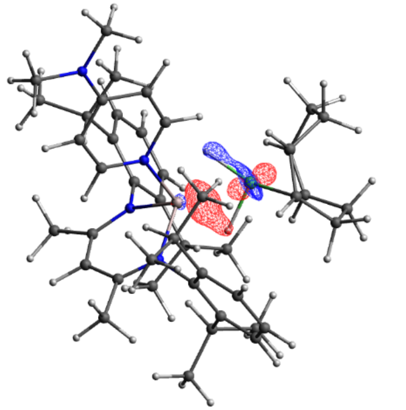

Table S15 ETS-NOCV analysis for **2·DMAP** using fragmentation a. ΔE_{orb} = -85.1 kcal mol^{-1}.

S7.4.6 IBO analysis

IBO	Isosurface
**Al–N^{BDI} σ**<br>55.6 % N^{BDI} (12.6 % s, 25.4 % p_x, 16.9 % p_y)<br>21.3 % Al (3.5 % s, 5.4 % p_x, 4.7 % p_y, 4.7 % d_{xy})	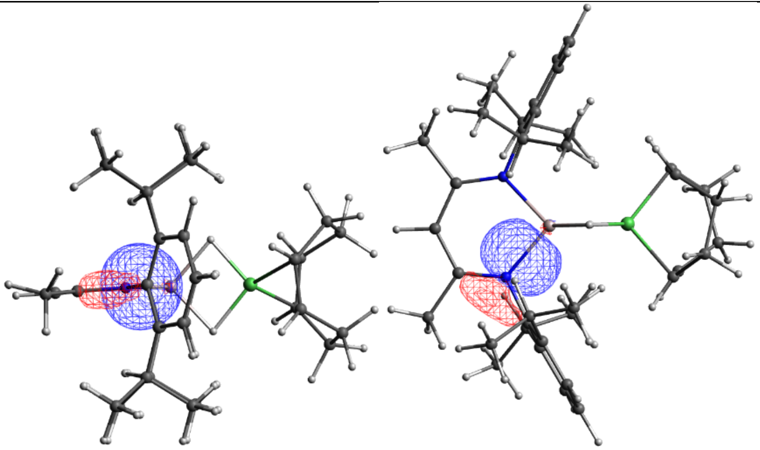
**Al–N^{BDI} σ**<br>55.7 N^{BDI} (12.6 % s, 24.9 % p_x, 17.5 % p_y)<br>22.0 % Al (3.5 % s, 5.2 % p_x, 4.8 % p_y, 4.7 % d_{xy})	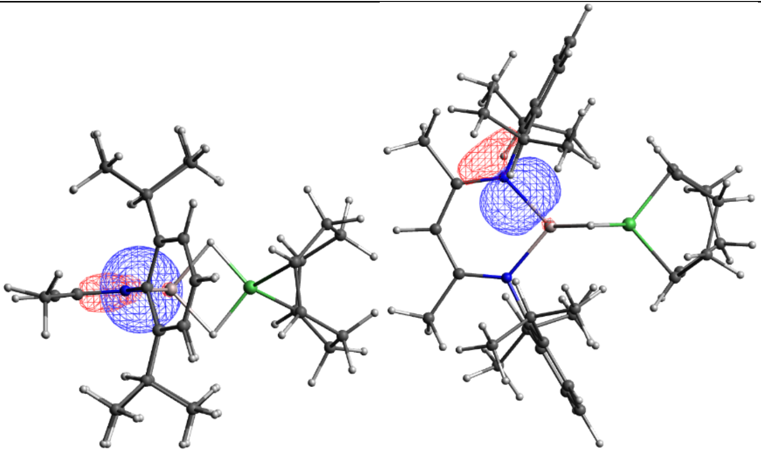
**Al–H^A–Ni σ**<br>37.9 % H^A (37.4 % s)<br>20 % Al (8.0 % s, 5.3 % p_y, 4.4 % d_{yz})<br>23.3 % Ni (4.3 % s, 7.0 % p_z5.9 % p_y, 3.9 % d_{yz})	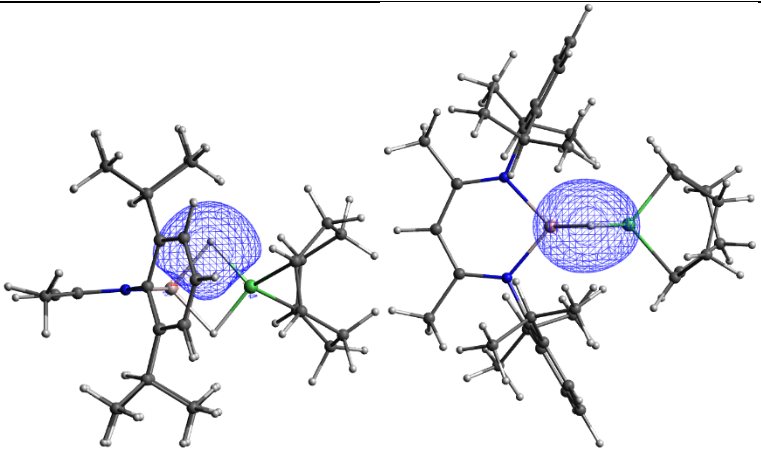

Table S16 IBO analysis of **2**. Isovalue = 0.05.

**Al–H^{B}–Ni σ**<br>38.1 % H^{B} (37.6 % s)<br>25.9 % Al (6.2 % s, 8.1 % p$_{z}$, 5.1 % p$_{y}$, 4.3 % d$_{yz}$)	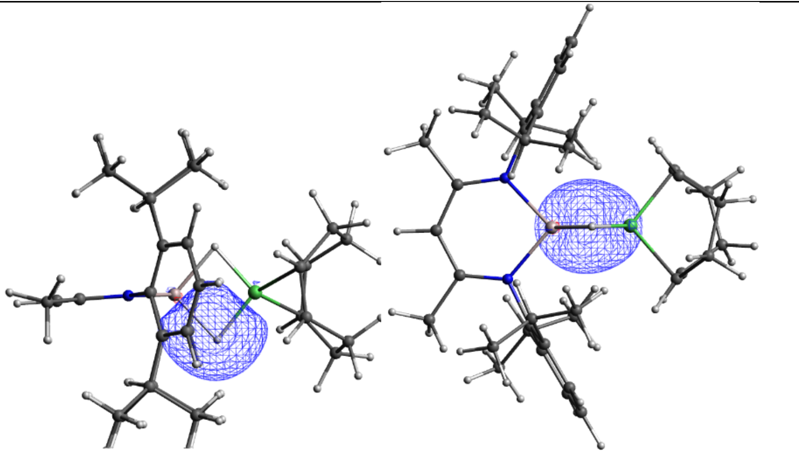
**Ni d$_{x2-y2}$**<br>91.9 % Ni (84.3 % d$_{x2-y2}$, 5.2 % d$_{xy}$)	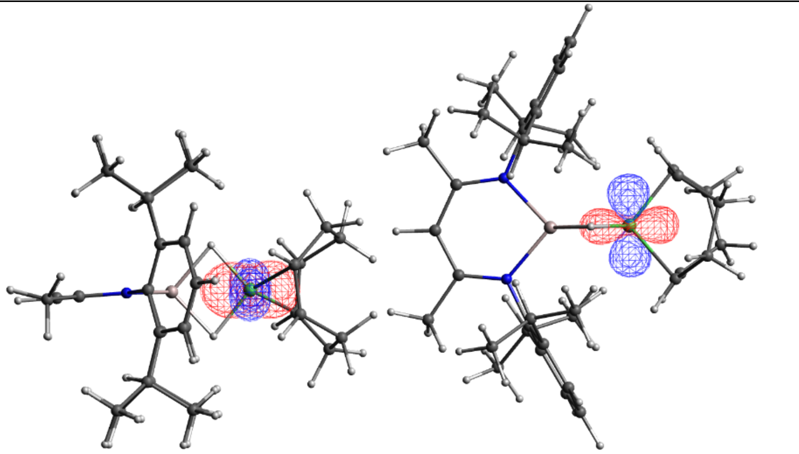
**Ni d$_{xy}$**<br>85.3 % Ni (82.6 % d$_{xy}$)	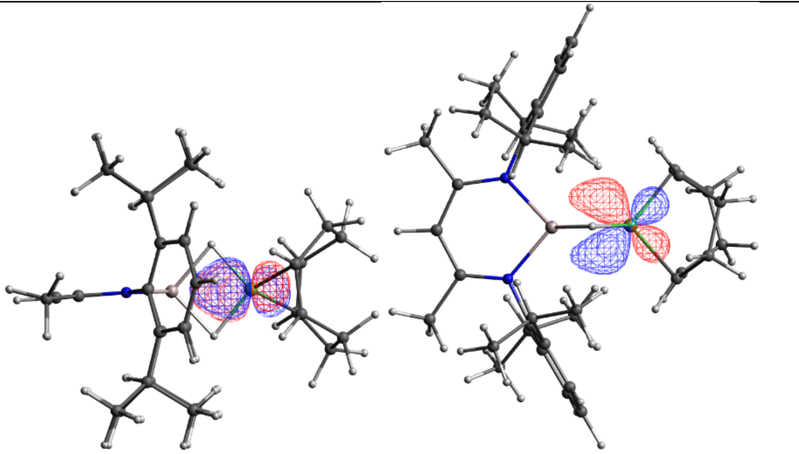

Table S16 continued. IBO analysis of **2**. Isovalue = 0.05.

**Ni d$_{z2}$**<br><br>90.5 % Ni (87.1 % d$_{z2}$)	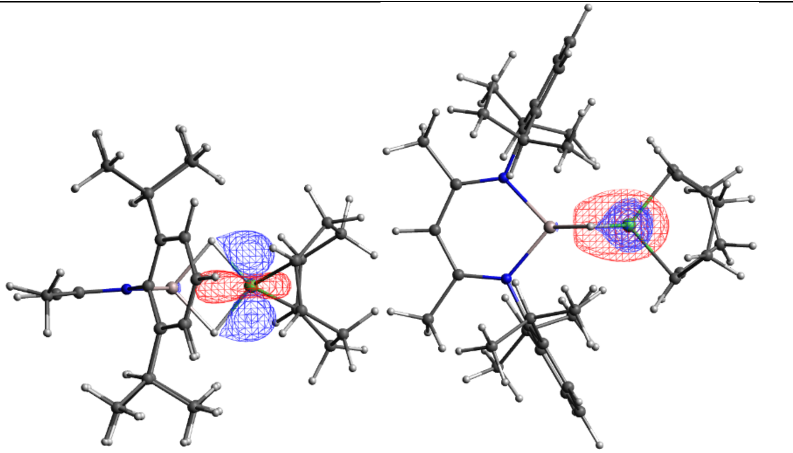
**Ni d$_{xz}$ – C$_{alkene}$**<br><br>82.7 % Ni (77.3 % d$_{xz}$)<br><br>10.8 % $\sum$C$_{alkene}$	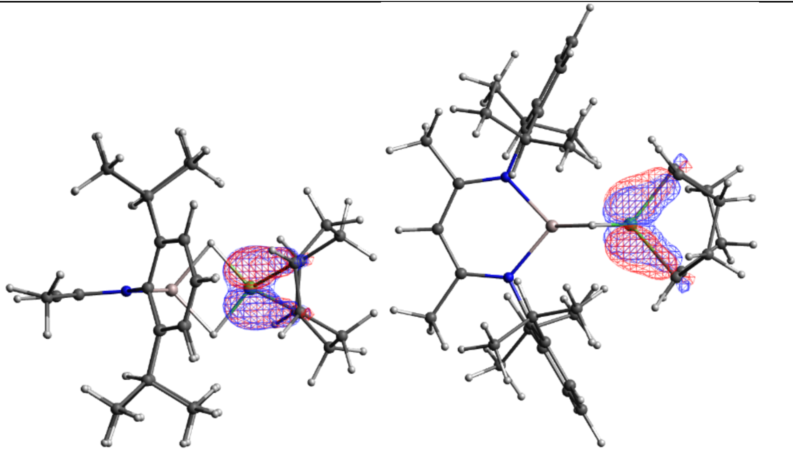
**Ni d$_{yz}$ – C$_{alkene}$**<br><br>76.3 % Ni (74.1 % d$_{yz}$)<br><br>12.8 % $\sum$C$_{alkene}$	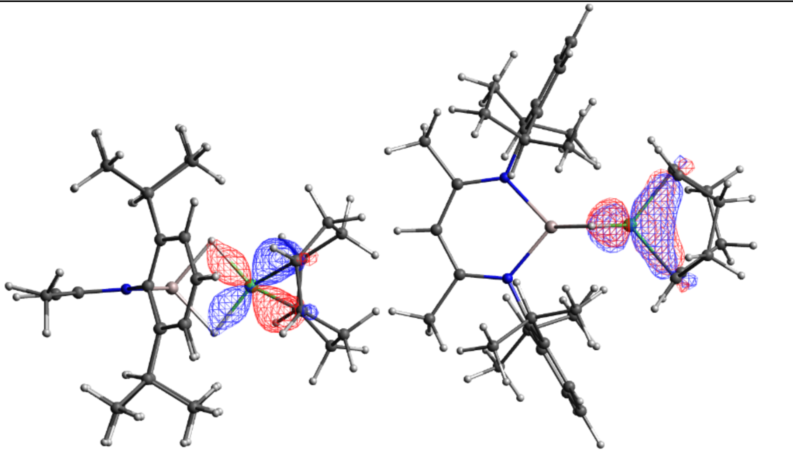

Table S16 continued. IBO analysis of **2**. Isovalue = 0.05.

**IBO**	**Isosurface**
**Al–N^{BDI} σ**<br>55.8 % N^{BDI} (13.8 % s, 41.1 % p_z)<br>20.6 % Al (3.0 % s, 9.2 % p_z, 5.8 % d_{z2})	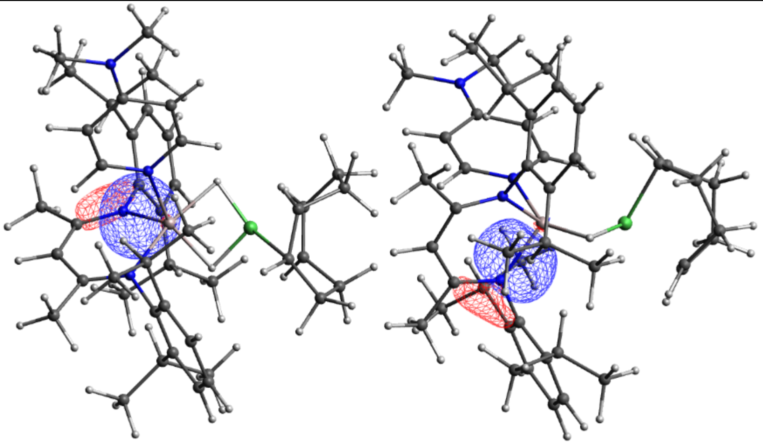
**Al–N^{BDI} σ**<br>55.7 N^{BDI} (12.6 % s, 24.9 % p_x, 17.5 % p_y)<br>20.1 % Al (2.8 % s, 7.4 % p_y, 2.4 % d_{x2y2}, 2.4 % d_{xy})	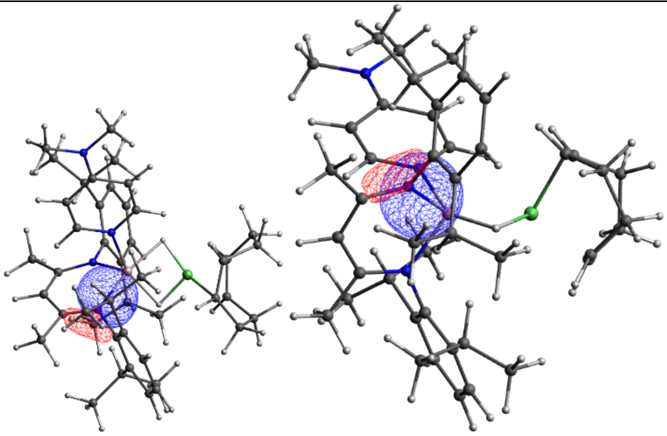
**Al–N^{DMAP} σ**<br>55.7 N^{DMAP} (12.6 % s, 24.9 % p_x, 17.5 % p_y)<br>19.1 % Al (2.3 % s, 6.7 % p_x, 3.5 % d_{x2y2})	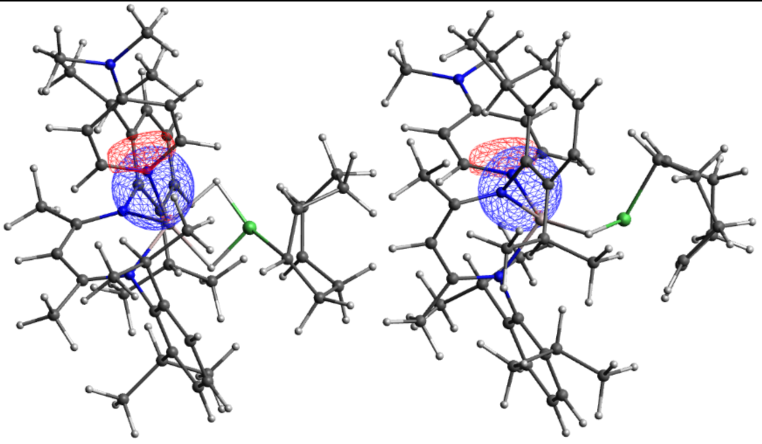

Table S17 IBO analysis of **2·DMAP**. Isovalue = 0.05.

**Al–H^{A}–Ni σ**<br><br>36.1 % H^{A} (35.8 % s)<br><br>22.9 % Al (4.7 % s, 6.8 % p_x)<br><br>23.2 % Ni (4.5 % s, 4.5 % p_x, 7.6 % py)	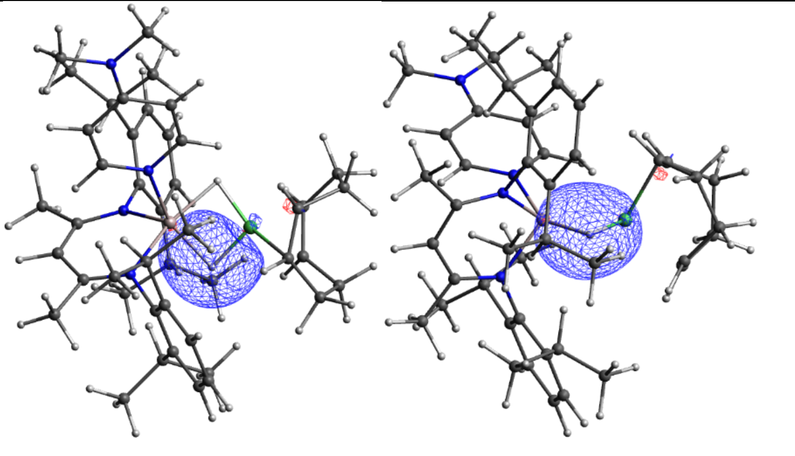
**Al–H^{B}–Ni σ**<br><br>36.8 % H^{A} (36.6 % s)<br><br>23.3 % Al (4.4 % s, 8.8 % p_y, 3.7 % d_{x2-y2})<br><br>23.5 % Ni (4.4 % s, 6.4% p_z, 7.6 % p_x, 3.2 % d_{xz})	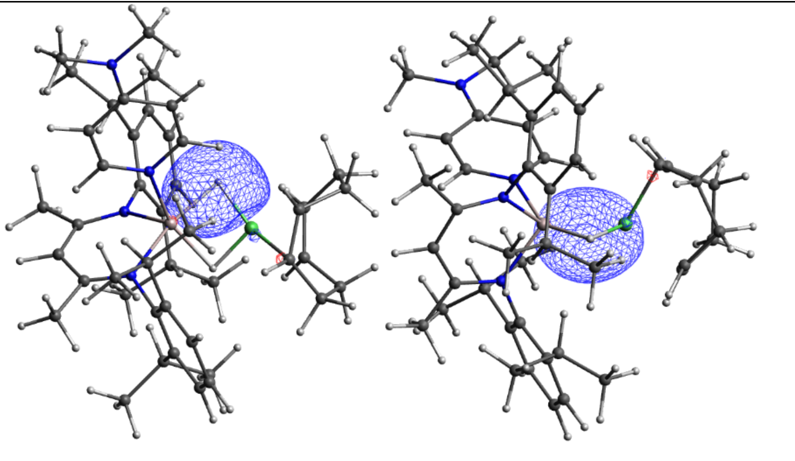
**Ni d_{yz}**<br><br>82.3 % Ni (49.7 % d_{yz}, 29.7 % d_{xz}, 6.8 % d_{xy}, 4.2 % d_{x2-y2})<br><br>6.4 % Al (2.3 % p_z, 1.9 % d_{z2})	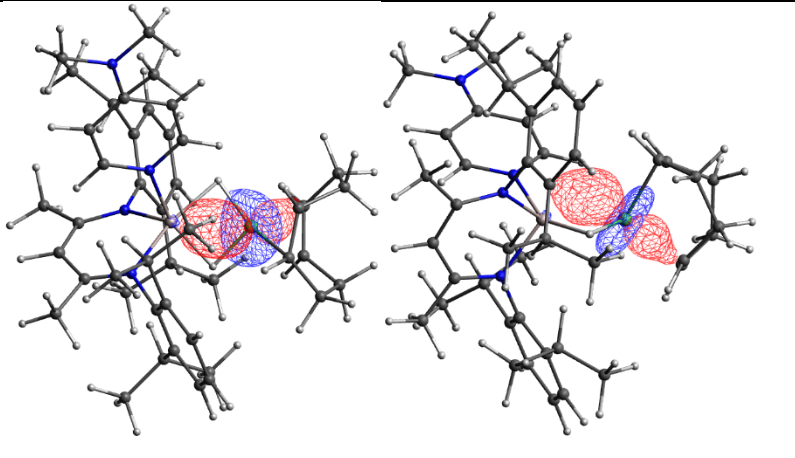

Table S17 continued. IBO analysis of **2·DMAP**. Isovalue = 0.05.

**Ni d_{xz} – C_{alkene}**<br><br>74.0 % N^{BDI} (43.9 % d_{xz}, 13.8 % d_{xy}, 8.8 % d_{yz}, 4.2 % d_{z2})<br><br>9.3 % $\sum C_{alkene}$	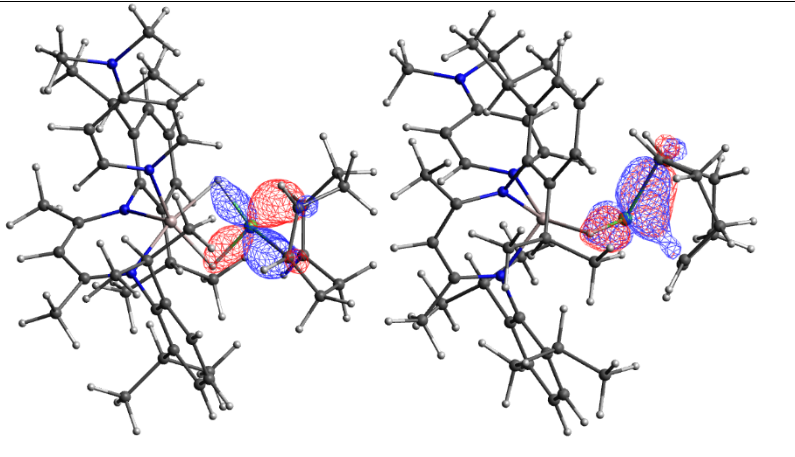
**Ni d_{z2}**<br><br>90.5 % Ni (51.3 % d_{z2}, 13.9 % d_{xy}, 10.1 % d_{yz}, 10.2 % d_{x2-y2}, 4.6 % d_{xz})	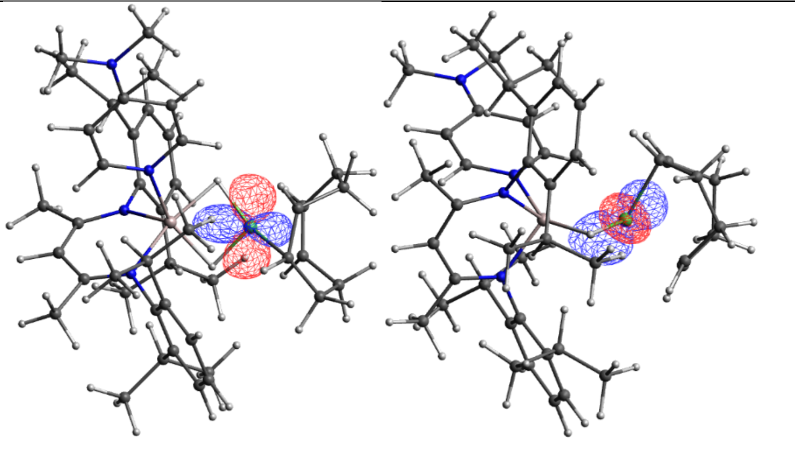
**Ni d_{yz}**<br><br>92.0 % Ni (49.7 % d_{yz}, 29.7 % d_{xz}, 6.8 % d_{xy}, 4.2 % d_{x2-y2})	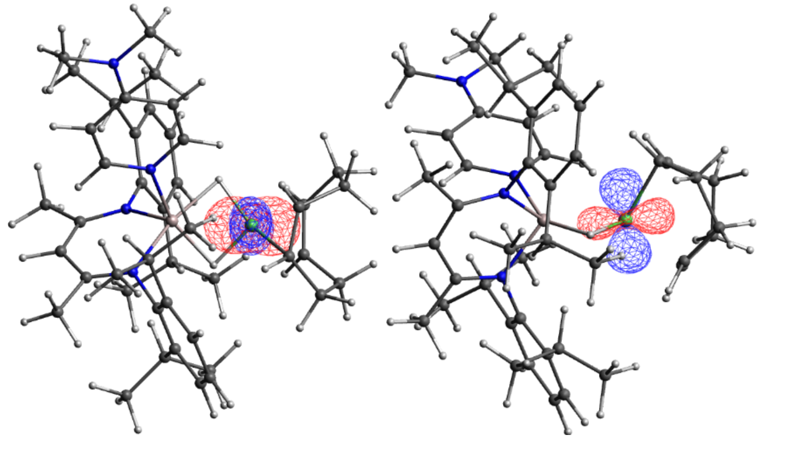

Table S17 continued. IBO analysis of **2·DMAP**. Isovalue = 0.05.

**Ni d** <br>83.3 % Ni (29.4 % d_{xy}, 26.1 % d_{x2-y2}, 17.4 % d_{yz}, 7.9 % d_{z2}, 2.4 % d_{xz})	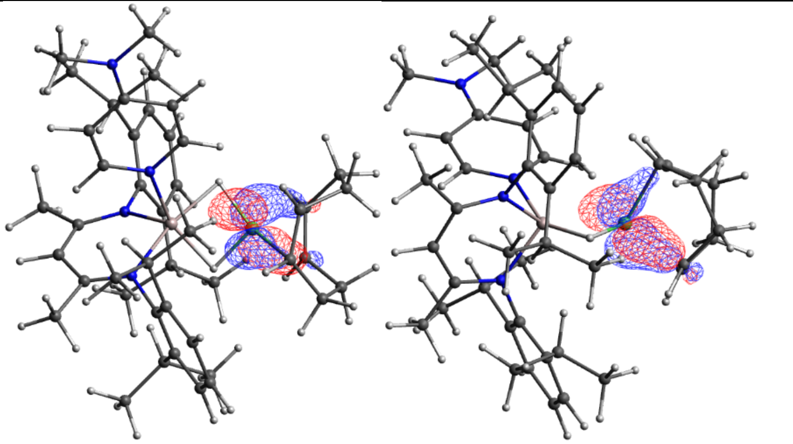

Table S17 continued. IBO analysis of **2·DMAP**. Isovalue = 0.05.

S7.4.7 ELF analysis of **2**

Other than the metal core density, ELF analysis identified four basins that contribute to the bonding in the heterometallic core. 2x trisynaptic basins are associated with the 3-centre-2-electron bonding through the two hydrides (containing 2.20 and 2.21 e^-, respectively), and 2x disynaptic basins perpendicular to the plane of the hydrides (containing 0.25 and 0.26 e^-, respectively), which describe the direct metal-metal bonding.

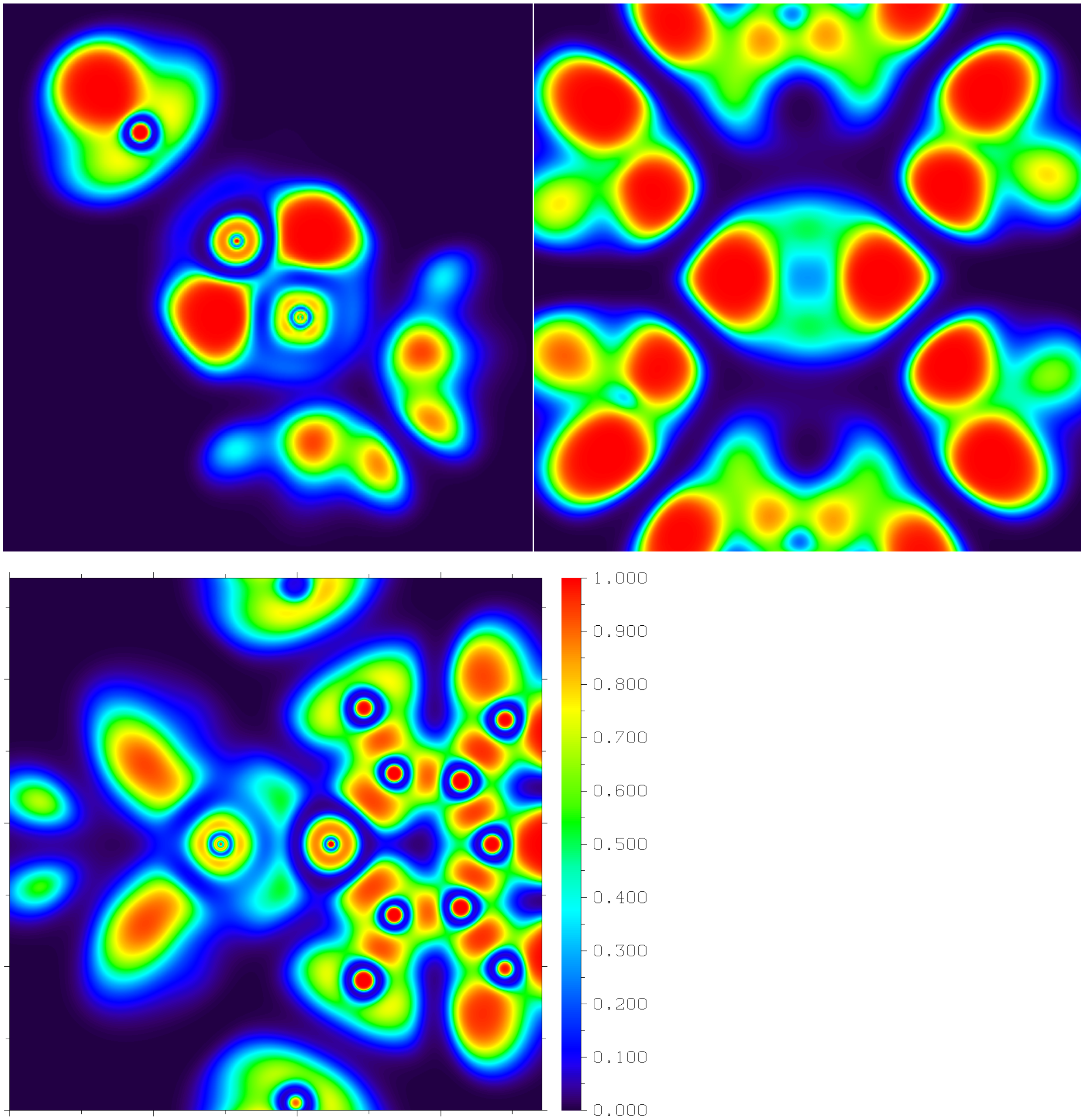

Figure S66 Visualisation of the ELF in **2**. Top left: in the Ni–H–Al plane, top right: in the H–H plane perpendicular to the Ni–H–Al plane, bottom left: in the Ni–Al plane, perpendicular to the Ni–H–Al plane.

S8 References

[1] H. Videa, A. J. Martinez-Martinez, "Contacted" vs "separated" aluminyls: Dynamic alkali metal effects in H_2 activation **2022**, DOI 10.26434/chemrxiv-2022-wjs4l-v2.

[2] F. P. Gabbaï, P. J. Chirik, D. E. Fogg, K. Meyer, D. J. Mindiola, L. L. Schafer, S.-L. You, An Editorial About Elemental Analysis *Organometallics* **2016**, *35*, 3255–3256.

[3] R. E. H. Kuveke, L. Barwise, Y. van Ingen, K. Vashisth, N. Roberts, S. S. Chitnis, J. L. Dutton, C. D. Martin, R. L. Melen, An International Study Evaluating Elemental Analysis *ACS Cent. Sci.* **2022**, *8*, 855–863.

[4] E. D. Entz, J. E. A. Russell, L. V. Hooker, S. R. Neufeldt, Small Phosphine Ligands Enable Selective Oxidative Addition of Ar–O over Ar–Cl Bonds at Nickel(0) *J. Am. Chem. Soc.* **2020**, *142*, 15454–15463.

[5] M. E. Greaves, E. L. B. Johnson Humphrey, D. J. Nelson, Reactions of nickel(0) with organochlorides, organobromides, and organoiodides: mechanisms and structure/reactivity relationships *Catal. Sci. Technol.* **2021**, *11*, 2980–2996.

[6] J. Cornella, E. Gómez-Bengoa, R. Martin, Combined Experimental and Theoretical Study on the Reductive Cleavage of Inert C–O Bonds with Silanes: Ruling out a Classical Ni(0)/Ni(II) Catalytic Couple and Evidence for Ni(I) Intermediates *J. Am. Chem. Soc.* **2013**, *135*, 1997–2009.

[7] G. M. Sheldrick, Crystal structure refinement with SHELXL *Acta Crystallogr. Sect. C Struct. Chem.* **2015**, *71*, 3–8.

[8] O. V. Dolomanov, L. J. Bourhis, R. J. Gildea, J. A. K. Howard, H. Puschmann, OLEX2 : a complete structure solution, refinement and analysis program *J. Appl. Crystallogr.* **2009**, *42*, 339–341.

[9] G. M. Sheldrick, SHELXT – Integrated space-group and crystal-structure determination *Acta Crystallogr. Sect. A Found. Adv.* **2015**, *71*, 3–8.

[10] C. Neese, F.; Wennmohs, F.; Becker, U.; Riplinger, F. Neese, F. Wennmohs, U. Becker, C. Riplinger, The ORCA Quantum Chemistry Program Package. *J. Chem. Phys.* **2020**, *152*, 224108.

[11] F. Neese, Software Update: The ORCA Program System—Version 6.0 *Wiley Interdiscip. Rev. Comput. Mol. Sci.* **2025**, *15*, e70019.

[12] S. Grimme, F. Bohle, A. Hansen, P. Pracht, S. Spicher, M. Stahn, Efficient Quantum Chemical Calculation of Structure Ensembles and Free Energies for Nonrigid Molecules *J. Phys. Chem. A* **2021**, *125*, 4039–4054.

[13] J. Sun, R. C. Remsing, Y. Zhang, Z. Sun, A. Ruzsinszky, H. Peng, Z. Yang, A. Paul, U. Waghmare, X. Wu, M. L. Klein, J. P. Perdew, SCAN: An Efficient Density Functional Yielding Accurate Structures and Energies of Diversely-Bonded Materials **2015**.

[14] A. P. Bartók, J. R. Yates, Regularized SCAN functional *J. Chem. Phys.* **2019**, *150*, 161101.

[15] J. W. Furness, A. D. Kaplan, J. Ning, J. P. Perdew, J. Sun, Accurate and Numerically Efficient r^{2}SCAN Meta-Generalized Gradient Approximation *J. Phys. Chem. Lett.* **2020**, *11*, 8208–8215.

[16] J. W. Furness, A. D. Kaplan, J. Ning, J. P. Perdew, J. Sun, Correction to "Accurate and Numerically Efficient r^{2}SCAN Meta-Generalized Gradient Approximation" *J. Phys. Chem. Lett.* **2020**, *11*, 9248–9248.

[17] J. W. Furness, A. D. Kaplan, J. Ning, J. P. Perdew, J. Sun, Construction of meta-GGA functionals through restoration of exact constraint adherence to regularized SCAN functionals *J. Chem. Phys.* **2022**, *156*, 34109.

[18] F. Weigend, R. Ahlrichs, Balanced basis sets of split valence, triple zeta valence and quadruple zeta valence quality for H to Rn: Design and assessment of accuracy *Phys. Chem. Chem. Phys.* **2005**, *7*, 3297.

[19] E. Caldeweyher, S. Ehlert, A. Hansen, H. Neugebauer, S. Spicher, C. Bannwarth, S. Grimme, A generally applicable atomic-charge dependent London dispersion correction *J. Chem. Phys.* **2019**, *150*, 154122.

[20] H. Kruse, S. Grimme, A geometrical correction for the inter- and intra-molecular basis set superposition error in Hartree-Fock and density functional theory calculations for large systems *J. Chem. Phys.* **2012**, *136*, 154101.

[21] F. Neese, An improvement of the resolution of the identity approximation for the formation of the Coulomb matrix *J. Comput. Chem.* **2003**, *24*, 1740–1747.

[22] F. Neese, F. Wennmohs, A. Hansen, U. Becker, Efficient, approximate and parallel Hartree–Fock and hybrid DFT calculations. A 'chain-of-spheres' algorithm for the Hartree–Fock exchange *Chem. Phys.* **2009**, *356*, 98–109.

[23] B. Helmich-Paris, B. de Souza, F. Neese, R. Izsák, An improved chain of spheres for exchange algorithm *J. Chem. Phys.* **2021**, *155*, 104109.

[24] F. Weigend, Accurate Coulomb-fitting basis sets for H to Rn *Phys. Chem. Chem. Phys.* **2006**, *8*, 1057–1065.

[25] K. Ishida, K. Morokuma, A. Komornicki, The intrinsic reaction coordinate. An ab initio calculation for HNC→HCN and H−+CH4→CH4+H− *J. Chem. Phys.* **1977**, *66*, 2153–2156.

[26] J. Tao, J. P. Perdew, V. N. Staroverov, G. E. Scuseria, Climbing the Density Functional Ladder: Nonempirical Meta–Generalized Gradient Approximation Designed for Molecules and Solids *Phys. Rev. Lett.* **2003**, *91*, 146401.

[27] A. V. Marenich, C. J. Cramer, D. G. Truhlar, Universal Solvation Model Based on Solute Electron Density and on a Continuum Model of the Solvent Defined by the Bulk Dielectric Constant and Atomic Surface Tensions *J. Phys. Chem. B* **2009**, *113*, 6378–6396.

[28] S. Grimme, Supramolecular Binding Thermodynamics by Dispersion-Corrected Density Functional Theory *Chem. Eur. J.* **2012**, *18*, 9955–9964.

[29] B. de Souza, GOAT: A Global Optimization Algorithm for Molecules and Atomic Clusters *Angew. Chem. Int. Ed.* **2025**, e202500393.

[30] C. Bannwarth, S. Ehlert, S. Grimme, GFN2-xTB—An Accurate and Broadly Parametrized Self-Consistent Tight-Binding Quantum Chemical Method with Multipole Electrostatics and Density-Dependent Dispersion Contributions *J. Chem. Theory Comput.* **2019**, *15*, 1652–1671.

[31] S. Ehlert, M. Stahn, S. Spicher, S. Grimme, Robust and Efficient Implicit Solvation Model for Fast Semiempirical Methods *J. Chem. Theory Comput.* **2021**, *17*, 4250–4261.

[32] S. Spicher, S. Grimme, Robust Atomistic Modeling of Materials, Organometallic, and Biochemical Systems *Angew. Chem. Int. Ed.* **2020**, *59*, 15665–15673.

[33] and F. W. E. D. Glendening, J, K. Badenhoop, A. E. Reed, J. E. Carpenter, J. A. Bohmann, C. M. Morales, P. Karafiloglou, C. R. Landis, NBO 7.0 **2018**.

[34] T. A. Keith, AIMAll **2013**, AIMAll (version 17.01.25).

[35] T. Lu, F. Chen, Multiwfn: A multifunctional wavefunction analyzer *J. Comput. Chem.* **2012**, *33*, 580–592.

[36] T. Lu, A comprehensive electron wavefunction analysis toolbox for chemists, Multiwfn *J. Chem. Phys.* **2024**, *161*, 82503.

[37] S. Grimme, A. Hansen, A Practicable Real-Space Measure and Visualization of Static Electron-Correlation Effects *Angew. Chem. Int. Ed.* **2015**, *54*, 12308–12313.

[38] M. P. Mitoraj, A. Michalak, T. Ziegler, A Combined Charge and Energy Decomposition Scheme for Bond Analysis *J. Chem. Theory Comput.* **2009**, *5*, 962–975.

[39] F. Neese, Software update: The ORCA program system—Version 5.0 *WIREs Comput. Mol. Sci.* **2022**, *12*, e1606.

[40] T. Verstraelen, S. Vandenbrande, F. Heidar-Zadeh, L. Vanduyfhuys, V. Van Speybroeck, M. Waroquier, P. W. Ayers, Minimal Basis Iterative Stockholder: Atoms in Molecules for Force-Field Development *J. Chem. Theory Comput.* **2016**, *12*, 3894–3912.

[41] G. Knizia, Intrinsic Atomic Orbitals: An Unbiased Bridge between Quantum Theory and Chemical Concepts *J. Chem. Theory Comput.* **2013**, *9*, 4834–4843.

[42] N. Gorgas, A. J. P. White, M. R. Crimmin, Cooperative C–H Bond Activation by a Low-Spin d^6 Iron–Aluminum Complex *J. Am. Chem. Soc.* **2022**, *144*, 8770–8777.
